# Diversification of clearwing butterflies with the rise of the Andes

**DOI:** 10.1111/jbi.12611

**Published:** 2015-09-24

**Authors:** Donna Lisa De‐Silva, Marianne Elias, Keith Willmott, James Mallet, Julia J. Day

**Affiliations:** ^1^ Institut de Systématique, Évolution, BiodiversitéISYEB – UMR 7205 – CNRSMNHNUPMCEPHEMuséum National d'Histoire NaturelleSorbonne Universités57 rue Cuvier, CP50ParisF‐75005France; ^2^ Department of Genetics, Evolution and EnvironmentUniversity College LondonDarwin BuildingGower StreetLondonWC1E 6BTUK; ^3^ McGuire Center for LepidopteraFlorida Museum of Natural HistoryUniversity of FloridaP.O. Box 112710GainesvilleFL32611‐2710USA; ^4^ Department of Organismic and Evolutionary BiologyHarvard University Biology Laboratories16 Divinity AvenueCambridgeMA02138USA

**Keywords:** Andes, biogeography, diversification, *Hyposcada*, Ithomiini butterflies, *Megoleria*, Neotropics, *Oleria*, *Ollantaya*

## Abstract

**Aim:**

Despite the greatest butterfly diversity on Earth occurring in the Neotropical Andes and Amazonia, there is still keen debate about the origins of this exceptional biota. A densely sampled calibrated phylogeny for a widespread butterfly subtribe, Oleriina (Nymphalidae: Ithomiini) was used to estimate the origin, colonization history and diversification of this species‐rich group.

**Location:**

Neotropics.

**Methods:**

Ancestral elevation and biogeographical ranges were reconstructed using data generated from detailed range maps and applying the dispersal‐extinction‐cladogenesis model using stratified palaeogeographical time slice matrices. The pattern of diversification through time was examined by comparing constant and variable rate models. We also tested the hypothesis that a change in elevation is associated with speciation.

**Results:**

The Oleriina likely originated in the Andes in the Early to Middle Miocene and rapidly diversified to include four genera all of which also originated in the Andes. These clades, together with four species groups, experienced varying spatial and temporal patterns of diversification. An overall early burst and decreasing diversification rate is identified, and this pattern is reflected for most subclades.

**Main conclusions:**

Changes in the palaeogeological landscape, particularly the prolonged uplift of the Andes, had a profound impact on the diversification of the subtribe. The Oleriina mostly remained within the Andes and vicariant speciation resulted in some instances. Dynamic dispersal occurred with the disappearance of geological barriers such as the Acre System and the subtribe exploited newly available habitats. Our results confirm the role of the Andean uplift in the evolution of Neotropical biodiversity.

## Introduction

Like all other species‐rich groups of organisms, the taxonomic diversity of butterflies is highest in the Neotropical region and is estimated at 7700 species (Lamas, 2004). The majority of these species are found in the tropical Andes of Colombia, Ecuador, Peru and the western Amazonian lowlands, but the origin and diversification patterns of most groups remain poorly understood. The complex geological history of the Neotropics, together with palaeoclimatic changes, have created a biogeographically diverse region with a mosaic of ecosystems and habitats (Gentry, 1982; Turchetto‐Zolet *et al*., 2013). Undoubtedly, this history had a profound influence on the origin of much of the region's endemic biodiversity and shaped diversification processes (Hoorn *et al*., 2010; Blandin & Purser, 2013; Rull, 2013).

Several major palaeogeographical events potentially played a prominent role in the evolution of Amazonian biodiversity (Hoorn *et al*., 2010): the discontinuous uplift of the tropical Andes, which began with sustained uplift of the Central Andes from the Late Oligocene ~26 million years ago (Ma; Sébrier *et al*., 1988; Ehlers & Poulsen, 2009), although increasing evidence suggests more rapid growth from the Late Miocene (12 Ma; Kennan *et al*., 1997; Garzione *et al*., 2008, 2014; Whipple & Gasparini, 2014); the orogenesis of the northern Colombian Andes began with the Cordillera Occidental and culminated with the emergence of the Cordillera Oriental (15 to 3 Ma; Mora *et al*., 2008); the formation of the ‘Pebas System’, a vast network of shallow lakes and wetlands, from the Late Oligocene to the Early Miocene (*c. *24 to 11.3 Ma) (Wesselingh *et al*., 2002); the formation of the fluvial ‘Acre’ System (*c*. 11.3 to 7 Ma), which later became the eastward flowing palaeo‐Amazon (*c*. 8 to 5 Ma), after intense Andean uplift created a continuous barrier and transformed Amazonian drainage patterns (Mora *et al*., 2010).

Studies of Neotropical diversification have suggested that the lowlands have been an important source of biodiversity, with diversification driven by riverine barriers (Ayres & Clutton‐Brock, 1992; Rosser *et al*., 2012), marine incursions (Hoorn, 2006) and environmental heterogeneity (Tuomisto *et al*., 1995). However, mounting evidence suggests that the Andean orogeny and geologically dynamic areas, in general, (Hoorn *et al*., 2013) have also promoted diversification through allopatric speciation while also presenting new opportunities for ecological adaptation (Gentry, 1982; Kattan *et al*., 2004; Elias *et al*., 2009; Santos *et al*., 2009).

A limited number of Neotropical butterfly studies have indicated that the Andes were an important source for new species. *Hypanartia* (Nymphalidae: Nymphalinae) diversified entirely within the Andes with speciation occurring without significant elevational changes (Willmott *et al*., 2001). Similarly, Andean *Lymanopoda* (Nymphalidae: Satyrinae) diversified within elevational bands, radiating horizontally throughout the Andes with occasional speciation across elevational boundaries (Casner & Pyrcz, 2010). The ithomiine genera, *Napeogenes* and *Ithomia*, originated at middle elevations in the Andes probably through ecological adaptation, although vicariance caused by Andean uplift was also detected (Elias *et al*., 2009). The diversification of *Taygetis* (Nymphalidae: Satyrinae) in the Late Miocene to Pliocene was coincident with the central Andean uplift and the disappearance of geographical barriers such as Lake Pebas (Matos‐Maraví *et al*., 2013). The Andes also played an important role in the radiation of heliconiine butterflies (Nymphalidae: Heliconiinae) and the diversification of the most species‐rich genera is coincident with uplift of the Andes (Kozak *et al*., 2015). Nevertheless, the highest community species richness in most groups is found in the Amazon Basin, where, for example, a large proportion of heliconiine subspecies diversity occurs (Rosser *et al*., 2012).

To provide further insights into how geological events have shaped Neotropical butterfly diversification processes, we studied the evolutionary history of the diverse butterfly subtribe Oleriina (Nymphalidae: Ithomiini). The Ithomiini represent an ideal group for this purpose because they are widely distributed throughout the Neotropics and are found at all elevations within the Andean mountain range up to around 3000 m. The overall diversity and distribution of the tribe is reflected in the subtribe Oleriina, containing the most species‐rich ithomiine genus, *Oleria* (48 species), as well as three genera, *Hyposcada*,* Megoleria* and *Ollantaya*, that are relatively species‐poor in comparison. The subtribe therefore offers a valuable system to investigate diversification processes in ithomiine butterflies and may aid our understanding of the diversification of Neotropical butterflies as a whole.

Using a densely sampled (86%), calibrated species‐level phylogeny combined with detailed biogeographical and elevation range data, we specifically address the following questions: (1) When and where did the Oleriina originate? (2) What was the biogeographical pattern of colonization of the Neotropics? (3) Did geological events, particularly the uplift of the Andes, influence the timing and pattern of diversification of the focal group?

## Materials and Methods

### Phylogenetic analysis of the Oleriina and timing of diversification

We generated a calibrated species‐level phylogeny using an uncorrelated lognormal relaxed clock implemented in beast 1.7.2 (Drummond *et al*., 2012) and implementing secondary calibrations based on Wahlberg *et al*. (2009) (see Appendix S1 in Supporting Information).

### Rates of diversification

To visualize the tempo of diversification, lineage‐through‐time (LTT) plots for 1000 sampled trees from the posterior distribution were generated in ape 3.0‐9 (Paradis *et al*., 2004) in R (R Core Team, 2015). To test whether diversification rates have changed over time the gamma (γ) statistic was calculated (Pybus & Harvey, 2000) for different taxonomic groups using ape 3.0‐9. Positive and negative values of γ indicate an increasing and decreasing diversification rate towards the present respectively. The Monte Carlo constant rates test (Pybus & Harvey, 2000) was used to determine if the decreasing diversification rate indicated by the gamma parameter is significant given the number of missing taxa in our dataset. laser 2.3 (Rabosky, 2006) was used to test specific models of diversification through time (see Table 1). Fit of constant and variable rate models were compared using the Akaike information criterion (AIC; see Table 1).

**Table 1:** Rates of diversification test results using rate‐constant (pure‐birth, birth‐death) and rate‐variable [density‐dependent logistic (DDL) and density‐dependent exponential (DDX), indicative of adaptive radiation; Yule two‐rate models; time‐varying speciation and constant extinction (SPVAR), time‐varying extinction and constant speciation (EXVAR), varying speciation and extinction through time (BOTHVAR; Rabosky & Lovette, 2008)] diversification models. In each case, the best model/s is/are indicated in bold. Log‐likelihood (AIC) and the difference in AIC with the best model (∆AIC) are shown for each clade examined. *R* ^1^ and *R* ^2^ indicate initial and, when applicable, final net diversification rates respectively; st = the time of rate shift in the Yule‐2‐rate model; *a* = extinction fraction *E/S*,* k* = the k‐parameter from the DDL model, and *x* = the *x*‐parameter from the DDX model

Clade	Model	*R* ^1^	*R* ^2^	*a*	*k*/*x*	st	log‐lkh	AIC	∆AIC
Oleriina	Pure‐birth	0.174					17.42	−32.84	11.71
	Birth‐death	0.174		0			17.42	−30.84	13.71
	**DDL**	**0.390**	** **	** **	**63.35**	** **	**24.16**	−**44.32**	**0.23**
	DDX	0.662			0.41		20.67	−37.33	7.21
	**Yule 2‐rate**	**0.230**	**0.045**			**1.75**	**25.27**	−**44.54**	**0**
	SPVAR						20.75	−35.51	9.04
	EXVAR						17.38	−28.77	15.77
	BOTHVAR						20.77	−33.53	11.01
***Hyposcada***	**Pure‐birth**	**0.101**					−**9.88**	**21.75**	**0**
	Birth‐death	0.101		0			−9.88	23.75	2
	**DDL**	**0.273**			**7.42**		**−9.29**	**22.58**	**0.82**
	**DDX**	**0.253**			**0.66**		**−9.66**	**23.33**	**1.57**
	Yule 2‐rate	0.081	0.121			3.96	−9.78	25.56	3.81
	SPVAR						−9.88	25.75	4
	EXVAR						−9.88	25.75	4
	BOTHVAR						−9.88	27.75	6
***Oleria***	Pure‐birth	0.205					10.66	−19.33	19.34
	Birth‐death	0.205		0			10.66	−17.33	21.34
	**DDL**	**0.626**			**44.18**		**21.33**	−**38.66**	**0**
	DDX	2.136			0.76		18.01	−32.02	6.64
	Yule 2‐rate	0.513	0.118			4.54	20.93	−35.85	2.81
	SPVAR						19.09	−32.17	6.49
	EXVAR						10.64	−15.27	23.39
	BOTHVAR						19.09	−30.17	8.49
***makrena*** **species group**	Pure‐birth	0.191					−8.76	19.52	16.42
	Birth‐death	0.191		0			−8.76	21.52	18.42
	**DDL**	**0.926**	** **	** **	**18.58**	** **	**0.31**	**3.38**	**0.27**
	DDX	5.475			1.38		−1.79	7.58	4.48
	**Yule 2‐rate**	**0.898**	**0.090**			**4.99**	**1.45**	**3.10**	**0**
	SPVAR						−1.60	9.21	6.1
	EXVAR						−8.76	23.52	20.42
	BOTHVAR						−1.62	11.24	8.14
***onega*** **species group**	Pure‐birth	0.190					−9.38	20.75	7.03
	Birth‐death	0.190		0			−9.38	22.75	9.03
	**DDL**	**0.696**	** **	** **	**15.01**	** **	−**5.16**	**14.32**	**0.6**
	DDX	1.291			0.90		−7.08	18.17	4.45
	**Yule 2‐rate**	**0.457**	**0.065**			**3.55**	−**3.86**	**13.72**	**0**
	SPVAR						−7.31	20.62	6.9
	EXVAR						−9.38	24.77	11.05
	BOTHVAR						−7.31	22.62	8.9
***amalda*** **species group**	**Pure‐birth**	**0.214**					−**6.13**	**14.25**	**0**
	Birth‐death	0.214		0			−6.13	16.25	2
	**DDL**	**0.544**			**7.62**		**−5.62**	**15.25**	**0.99**
	**DDX**	**0.398**			**0.45**		**−6.00**	**16.00**	**1.75**
	Yule 2‐rate	0.290	0.154			2.12	−5.88	17.75	3.5
	SPVAR						−6.13	18.25	4
	EXVAR						−6.13	18.25	4
	BOTHVAR						−6.13	20.25	6

### Ancestral elevation range

To test the hypothesis that a change in elevation is associated with speciation, we used bayestraits 1.0 (Pagel *et al*., 2004). BayesMultiState, which reconstructs the evolution of a finite number of discrete states, was implemented to compare a scenario of gradual evolution [where the branch scaling parameter kappa (κ) is equal to one], with a scenario of punctuational evolution (κ is equal to zero), and a scenario where κ was estimated by maximum likelihood (ML). Under a model of gradual evolution the probability of a change in elevation is in direct proportion to the branch length, whereas under punctuational evolution a change in elevation is associated with speciation.

Information on the elevation range of Oleriina was obtained for each species (see Appendix S1). The ancestral elevation range of each species was reconstructed using ML and maximum parsimony (MP) using bayestraits 1.0 (Pagel *et al*., 2004) and mesquite 2.75 (Maddison & Maddison, 2011) respectively. Each species was categorized according to its known elevation range as either low (0–1000 m), mid (750–1700 m), high (>1400 m) or a combination of these ranges (see Appendix S1). Using bayestraits 1.0 (Pagel *et al*., 2004) we first tested ancestral elevation as a discrete trait using BayesMultiState. Ancestral states were reconstructed by fixing the root of each clade at low, mid and high elevation and calculating the AIC in each case to determine if one of the alternative states was significantly more likely. The results obtained for κ were used to reconstruct the ancestral elevation range. Second, BayesContinuous was used to test ancestral elevation as a continuous trait by examining the minimum and maximum elevation range for each species. A random‐walk model of evolution was implemented and the branch scaling parameters κ, delta (δ) and lambda (λ) were estimated. The δ parameter determines if the rate of trait evolution has accelerated or slowed over time. A δ value of less than one is a signature of rapid early diversification followed by slower rates of change and suggests adaptive radiation, whereas a δ value of greater than one indicates a signature of accelerating evolution with time. The parameter λ reveals the phylogenetic signal for a given phylogeny and trait. A λ value of one is consistent with the Brownian motion or constant‐variance model of evolution, whereas a value of zero indicates that species are evolving independently. The log‐likelihood was calculated in each case and the associated AIC determined.

### Ancestral geographical distribution

**Figure 1:**
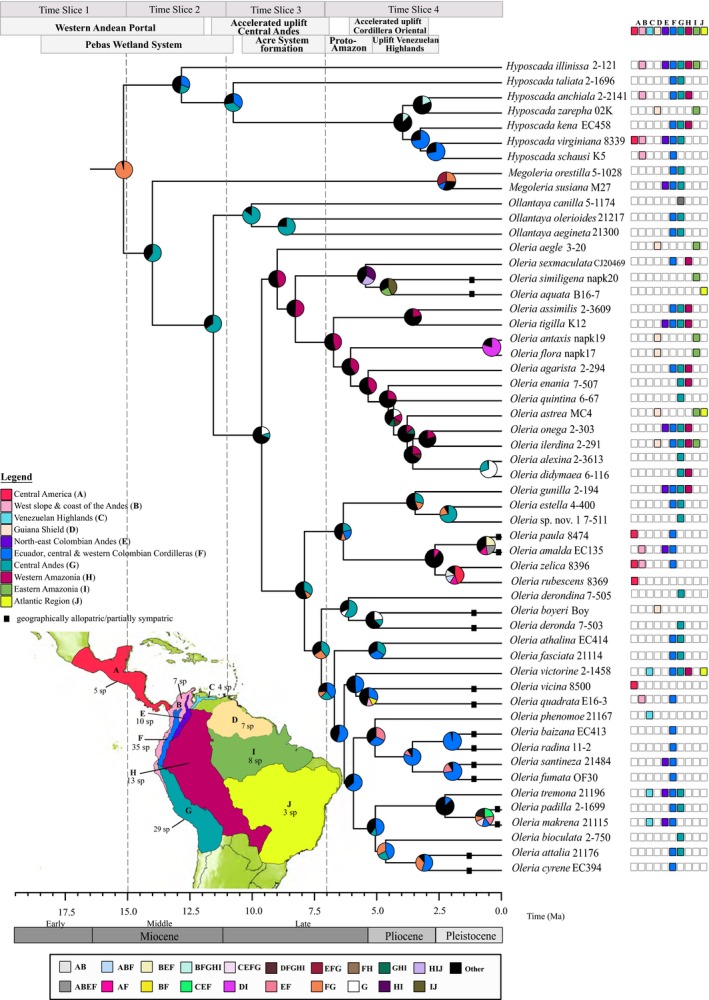
Palaeogeographical model used in the dispersal‐extinction‐cladogenesis analyses of biogeographical events. The four time slices used in the analyses and dispersal rates for each biogeographical area are shown. Maps are modified from Hoorn & Wesseling (2010); Condamine *et al*. (2013); Matos‐Maraví *et al*. (2013). Maps show dispersal and diversification of each Oleriina genus. Dispersal rates highlighted indicate a constraint on dispersal. (A) Central America; (B) western slopes and lowlands of the Northern Andes including Colombia, Ecuador and north‐west Peru; (C) Venezuelan Highlands including the Cordillera de Mérida and Cordillera de la Costa; (D) Guiana Shield; (E) north‐east Colombian Andes; (F) Northern Andes including Ecuador and the central & western Colombian Cordilleras; (G) Central Andes; (H) western Amazonia; (I) central and eastern Amazonia; (J) Atlantic region including the Atlantic Forest and Cerrado.

**Figure 2:**
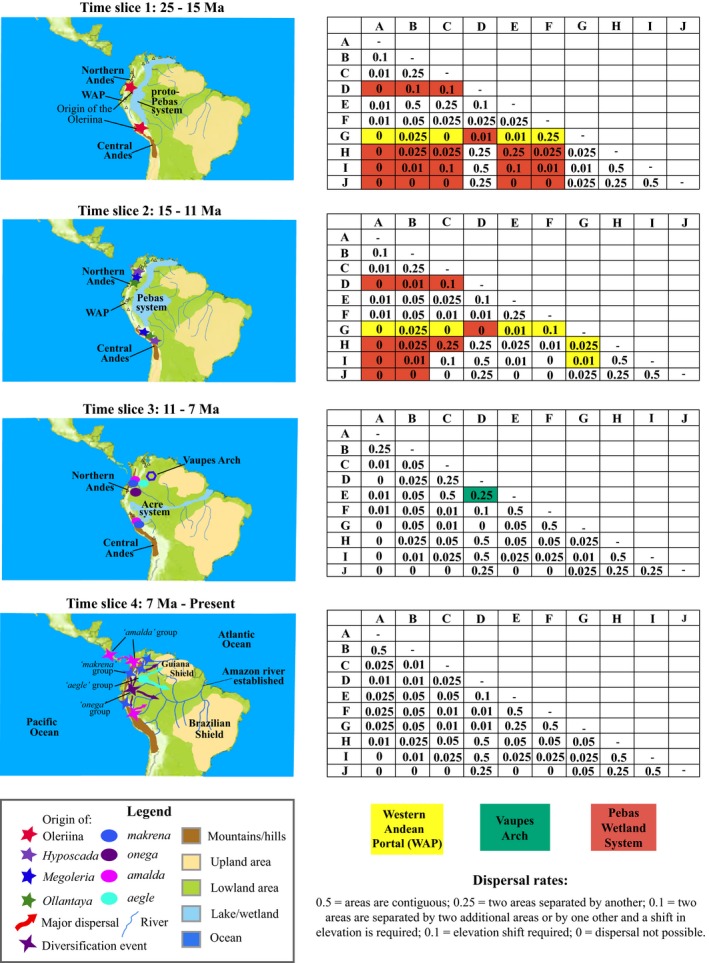
Bayesian dated maximum clade credibility tree for the Oleriina based on an uncorrelated lognormal relaxed clock detailing biogeographical reconstructions of ancestral geographical ranges inferred from dispersal‐extinction‐cladogenesis implemented in lagrange. The evolution of the Oleriina was divided into four time slices corresponding to notable palaeogeographical events. Coloured squares to the left of the map correspond to the geographical areas indicated. Coloured squares to the right of the tree indicate the geographical range of each extant species and those at the bottom of the tree indicate combined ranges. Pie charts represent the relative probabilities of ancestral ranges. Ancestral area probabilities < 0.1 were combined (black sections of the pie charts). The number of extant species for each geographical region is indicated in the map.

The distribution of the Oleriina (see Appendix S2) was delimited on the basis of geological history, range data and previous studies (Santos *et al*., 2009; Blandin & Purser, 2013) into ten areas (see Figs 1, 2). The geographical range evolution of the Oleriina was reconstructed using the ML dispersal‐extinction‐cladogenesis (DEC) model in lagrange (Ree & Smith, 2008). We used the dated phylogeny generated in beast and extant species distributional data to infer ancestral locations. The maximum number of ancestral areas was set to six, reflecting the maximum number of areas occupied by extant species. Each species was coded as present or absent for each geographical region. Our analyses considered the main palaeogeographical events that have occurred during the past 25 Myr (Fig. 2). This time span, covering the evolution of the Oleriina, was stratified into four time slices each reflecting temporal palaeogeographical constraints. We followed Ree & Smith (2008) and for each time slice a matrix was constructed to scale the probability of dispersal between 0 and 1 according to geographical area connectivity through time (Fig. 2). Dispersals that involved a change in elevation were therefore multiplied by 0.01 to reflect the low probability of movement (Matos‐Maraví *et al*., 2013). Additional analyses tested the root area of the subtribe by constraining the root to be single areas and combinations of multiple areas. Likelihoods of models under different constraints were compared. A log‐likelihood difference of two units was considered significant.

## Results

### Oleriina phylogeny and timing of diversification

**Figure 3:**
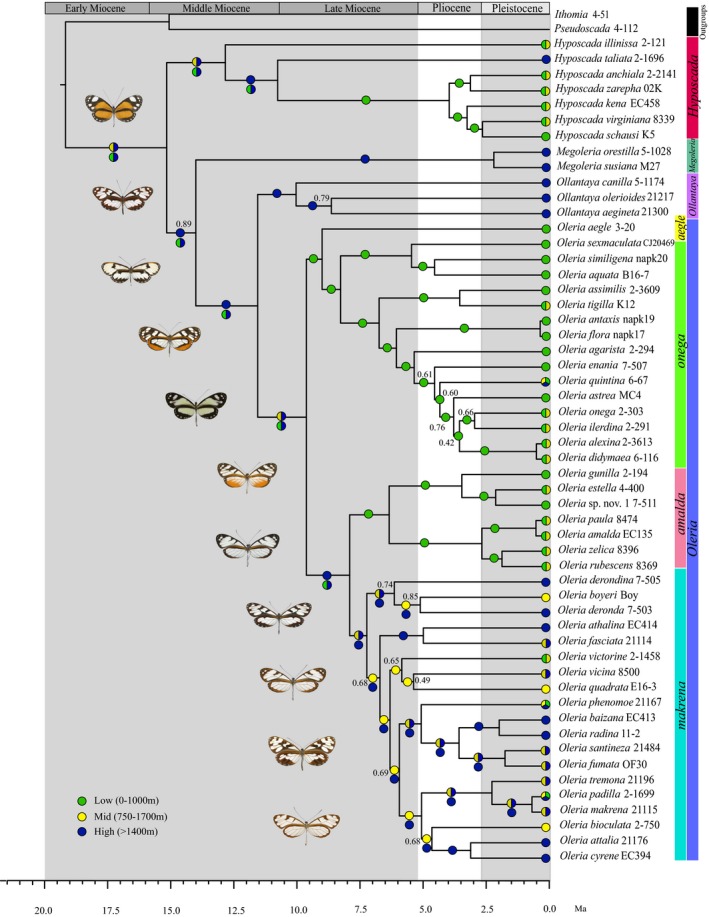
Bayesian dated maximum clade credibility tree for the Oleriina based on an uncorrelated log normal relaxed clock and detailing current and ancestral elevation ranges. Current elevation ranges are shown at the tips of the trees. ML inferred ancestral elevation ranges are shown for each node on the branch and MP inferred ancestral elevation ranges are shown below the branch where results differ. Bayesian posterior probabilities lower than 0.90 are indicated. Genera and *Oleria* species groups are shown to the right of the tree and photographs of Oleriina species are to the left. Elevation range categories are indicated in the legend.

Our analyses indicate that the Oleriina originated *c. *15.2 Ma [95% highest posterior density (HPD): 25.6–13.2 Ma] and that the diversification of all extant genera followed in the Middle to Late Miocene. The *Oleria* species groups, *onega* and *makrena*, diversified mainly during the Late Miocene and Pliocene (95% HPD: 8.0–3.0 Ma), with the *makrena* group diversifying further and more rapidly during the Pleistocene (95% HPD: 3.0–0.5 Ma). The Pleistocene was also important in the diversification of the *Oleria amalda* species group (Figs 1, 3).

### Diversification through time

**Figure 4:**
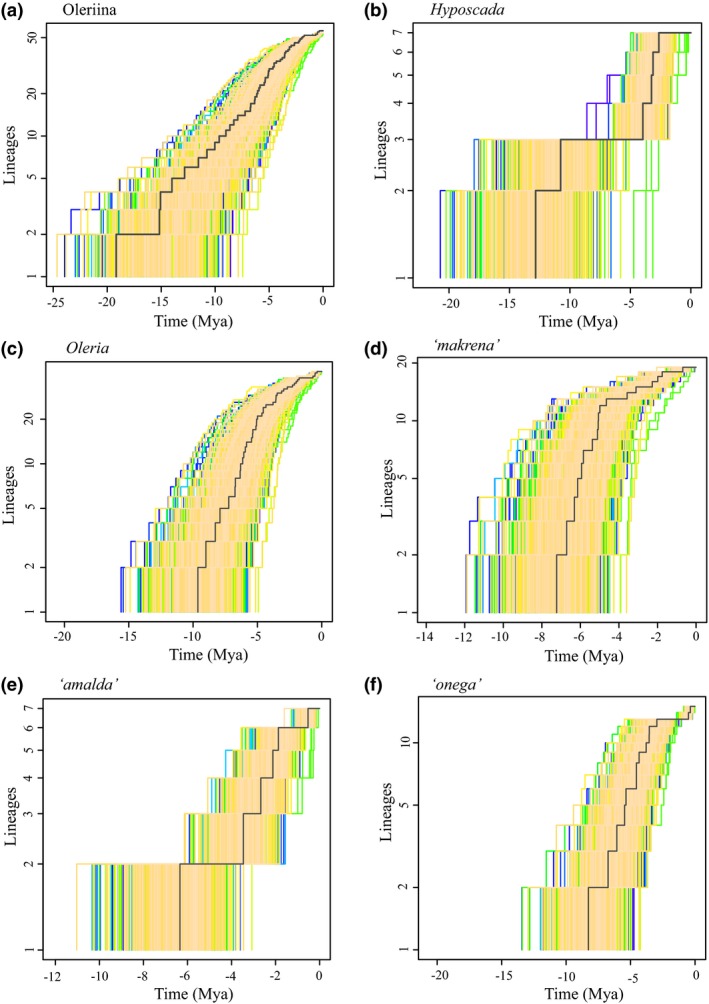
Lineage‐through‐time (LTT) plots based on 1000 sampled Bayesian trees for (a) Oleriina, (b) *Hyposcada*, (c) *Oleria*, (d) *makrena* species group, (e) *amalda* species group, (f) *onega* species group.

Lineage‐through‐time plots (Fig. 4) and the gamma statistic indicate an overall decreasing diversification rate for the Oleriina (γ = −3.38, *P *=* *0.001 assuming nine missing species). These results are reflected by the gamma statistic for *Oleria* (γ = −4.22, *P *=* *0.001, six missing species) as well as internal clades: *makrena* species group (γ = −3.57, *P *=* *0.001, three missing species); *onega* species group (γ = −2.31, *P *=* *0.01, three species missing). In turn, a constant diversification rate could not be rejected for the *amalda* species group (γ = −0.19, *P *=* *0.42) or for *Hyposcada* (γ = −0.27, *P *=* *0.28, two species missing). Gamma statistic results are in general supported by diversification models, in which rate‐variable models (density‐dependent logistic and/or Yule‐2‐rate) were preferred over rate‐constant models for Oleriina, *Oleria*,* makrena* and *onega* groups. Conversely, for *Hyposcada* and the *amalda* species group, a rate‐constant pure‐birth model was better supported although density‐dependent models also had high support. (Table 1).

### Evolution of elevation range

ML estimation of the scaling parameter κ was unable to reliably determine if evolution of ancestral elevation range as a discrete trait proceeded on a punctuational basis (κ = 0) or on a gradual basis (κ = 1) (ΔAIC_κ = 0 vs. κ = 1_ = 1.33). However, when excluding the species‐poor basal genus, *Hyposcada*, changes in elevation were found to be associated with speciation (ΔAIC_κ = 0 vs. κ = 1_ = 2.35). Estimating κ for the whole subtribe also confirmed this result. We therefore set κ to 0 for the ML reconstruction of elevation range.

Reconstruction of ancestral elevation was largely congruent between ML and MP analyses, except for basal nodes connecting the different genera, and some internal nodes in the makrena clade (Fig. 3). The Oleriina originated at mid or high elevations (Fig. 3, Table 2). This was followed by two radiations, one into mid/high elevations resulted in the genus *Hyposcada*. The second radiation led to the origin and diversification of *Megoleria*,* Ollantaya* and *Oleria*. A high elevation origin was well supported for *Megoleria* (Table 2). A second clade likely evolved at high elevation forming *Ollantaya* and *Oleria*. A high elevation origin was strongly supported for *Ollantaya,* whereas for *Oleria,* a mid‐ or high‐elevation origin was equally likely (Table 2). Within *Oleria*, two clades (the *amalda* group and the *onega + aegle* groups) colonized and mostly remained at low elevation with a few instances of movement to include mid elevation and one to high elevation. The fourth species group, *makrena*, originated at mid‐high elevations and diversified rapidly in montane areas.

**Table 2:** Tests of ancestral states of altitudinal ranges (scored as a discrete trait) in different clades. Low (0–1000 m), Mid (750–1700 m), High (> 1400 m). Log‐likelihood, AIC and the difference in AIC (∆AIC) are shown. In each case, the best model/s (∆AIC) is/are indicated in bold

Clade	Model	Log lkd	Parameters	AIC	∆AIC
**Oleriina**	**High**	−**21.09**	**4**	**50.17**	**0**
	Low	−23.19	4	54.38	4.21
	**Mid**	−**21.50**	**4**	**51.00**	**0.83**
***Hyposcada***	**High**	−**21.15**	**4**	**50.30**	**0**
	Low	−23.19	4	54.38	4.08
	**Mid**	−**21.18**	**4**	**50.36**	**0.06**
***Ollantaya***	**High**	−**20.69**	**4**	**49.37**	**0**
	Low	−25.67	4	59.33	9.96
	Mid	−22.05	4	52.09	7.24
***Oleria***	**High**	−**20.66**	**4**	**49.33**	**0**
	Low	−22.16	4	52.32	2.99
	**Mid**	−**20.99**	**4**	**49.98**	**0.65**
***Megoleria***	**High**	−**20.69**	**4**	**49.39**	**0**
	Low	−25.74	4	59.47	10.09
	Mid	−22.05	4	52.10	2.71

When elevation is scored as a continuous trait, for minimum elevation range and the κ and λ scaling parameters, the best model of evolution is a model without branch length transformation, meaning that evolution of elevation range is gradual and Brownian‐motion‐like (Table 3). For the δ scaling parameter, models δ = 1 and δ = 0.68 cannot be distinguished. Therefore, minimum elevation range either evolves at a constant rate, or at a slightly decelerating rate. For maximum elevation range, all values of the branch scaling parameters are equal or close to one, indicating a constant, Brownian‐motion‐like evolution.

**Table 3:** Maximum likelihood estimation of ancestral elevation range as a continuous trait for minimum and maximum elevation ranges. kappa (κ) = 0 (punctuational evolution), (κ) = 1 (gradual evolution); delta (δ) < 1 (early rapid evolution followed by slow down), (δ) > 1 (accelerating evolution with time); lambda (λ) = 1 (Brownian motion evolution), (λ) = 0 (Independent evolution). In each case, the best model is indicated in bold

Model	Log lkd	Parameters	AIC	∆AIC	Scaling parameter
Minimum elevation range					
** (κ) = 1**	−**400.71**	**2**	**805.41**	**0**	**1**
(κ) = 0	−406.55	2	817.09	11.68	0
(κ) = estimated	−400.69	3	807.37	2	0.95
** (δ) = 1**	−**400.71**	**2**	**805.41**	**0**	**1**
(δ) = 0	−451.62	2	907.24	101.83	0
** (δ) = estimated**	−**400.52**	**3**	**807.04**	**1.63**	**0.68**
** (λ) = 1**	−**400.71**	**2**	**805.41**	**0**	**1**
(λ) = 0	−424.35	2	852.70	47.29	0
(λ) = estimated	−400.71	3	807.41	2	1
Maximum elevation range					
** (κ) = 1**	−**407.27**	**2**	**818.55**	**0**	**1**
(κ) = 0	−410.83	2	825.65	7.10	0
** (κ) = estimated**	−**407.06**	**3**	**820.12**	**1.57**	**0.84**
** (δ) = 1**	−**407.27**	**2**	**818.55**	**0**	**1**
(δ) = 0	−440	2	884	65.45	0
(δ) = estimated	−407.27	3	820.54	2	1.04
** (λ) = 1**	−**407.27**	**2**	**818.55**	**0**	**1**
(λ) = 0	−427.66	2	859.33	40.78	0
** (λ) = estimated**	−**406.86**	**3**	**819.71**	**1.17**	**0.98**

### Ancestral geographical range

The ML DEC analyses recovered the Central Andes (G) (log *L* – 236.6) and the area spanning the Ecuadorian Andean ranges and Colombian central and western cordilleras (hereafter, Ecuador‐Colombia, Area F) (log *L* – 238.6) or both areas (log *L* – 237.2) as the most likely ancestral areas of the Oleriina (Fig. 1). Alternative root areas were not supported (more than 2 log‐likelihood units difference). Enforcing the root to include multiple areas recovered all combinations of two, three, four or five areas containing F and/or G as statistically supported (within 2 log‐likelihood units difference). Our analyses recovered a similar pattern when the root area was not enforced (Fig. 1).

Our results indicate that the Oleriina most likely diverged in situ forming all four genera (Figs 1, 2). *Hyposcada* dispersed widely with the exception of the Venezuelan Highlands and Atlantic region. The high elevation genera *Megoleria* and *Ollantaya* both remained in Ecuador‐Colombia (Area F) but only *Megoleria* reached the north‐east Colombian Andes. The *Oleria onega* and *aegle* groups diverged in western Amazonia, while, conversely, the *amalda* and *makrena* species groups are suggested to have evolved within the Central Andes or Ecuador‐Colombia (Area F). Within the *amalda* group there are two allopatric sub‐clades, the first is restricted to the eastern slopes of the Central Andes, north‐east Colombian Andes, Ecuador‐Colombia (Area F) and western Amazonia while the second is found on the western coast and slopes of the Andes and in Central America. The *makrena* group mostly remained within the Andean region. Notably, within this clade there are six instances of geographically allopatric or partially sympatric sister species (Fig. 1, see Appendix S1). In general, high numbers of sister species pairs are found in the same biogeographical region. The ML DEC analyses support a high rate of dispersal (0.31 per million years) and low extinction rate (0.04 per million years).

## Discussion

### Pattern and tempo of diversification

Overall, the Oleriina show a rapid early burst in diversification followed by a marked decreasing diversification rate during their history, which is also reflected in the genus *Oleria,* and the *makrena* and *onega* subclades. Decreasing diversification rates, particularly density‐dependent rates, have been interpreted as a signature of adaptive radiation (e.g. Rabosky & Lovette, 2008; Etienne *et al*., 2012; but see Pennell *et al*., 2012). A switch to the larval hostplant, Solanaceae, was likely a key event in the diversification of the ithomiine tribe as a whole, coupled with further specialization by mostly subtribal clades (Willmott & Freitas, 2006).

Oleriina, like all ithomiines, are chemically defended and co‐occurring species share warning wing colour patterns [Müllerian mimicry (Müller, 1879)]. Most ithomiine genera are diverse in wing pattern and mimicry is suggested as a driver of diversification in some clades (Jiggins *et al*., 2006; Elias *et al*., 2008). In *Heliconius* butterflies, sister species almost always differ in mimetic pattern and mimetic pattern has been shown to contribute to reproductive isolation (e.g. Merrill *et al*., 2012). In contrast to other subtribes, the Oleriina are probably the least diverse in wing pattern (Willmott & Mallet, 2004; Chazot *et al*., 2014) and, within individual communities, *Oleria* are often found to mimic one another (Elias *et al*., 2008; De‐Silva *et al*., 2010). Mimetic shifts are therefore unlikely to have enhanced diversification in the subtribe.

The rise of the Andes could also have driven adaptive radiation across the available elevational gradient, as suggested in the riodinid butterfly genus *Ithomiola* (Hall, 2005). However, in the case of Oleriina, although shifts in altitudinal ranges are associated with speciation (Table 1), they tend to be rare (Fig. 3). Moreover, tests of adaptive radiation linked to altitude were inconclusive (Table 3).

In contrast to other Oleriina clades, lineage accumulation in the *amalda* species group and *Hyposcada* is suggested to have proceeded at a near constant rate, although both clades contain a small number of species, potentially limiting the power to investigate diversification. The case of *Hyposcada* is particularly intriguing because it is an old clade that underwent a change in dietary repertoire with a switch to a new plant family, Gesneriaceae (Willmott & Freitas, 2006). Older clade age and increased ecological opportunities should both favour diversification, through time‐for‐speciation (Hutter *et al*., 2013) and adaptive radiation (Schulter, 2000; Kozak *et al*., 2015), yet this clade is particularly depauperate. Although no significant extinction was recovered at the subtribe level (again, perhaps because of a lack of power in small clades), the long branches leading to *H. illinissa* and *H. taliata*, and to the clade containing the remaining *Hyposcada*, may suggest past extinction in this genus.

### Rise of the Andes and biogeographical history of the Oleriina

Our analyses suggest that the ancestral Oleriina diverged from the rest of the Ithomiini during the Early to Middle Miocene between mid and high elevations in the Central Andes or Ecuador‐Colombia (Area F). The subtribe separated into four lineages, which, with the exception of *Megoleria*, all diverged during the Middle to Late Miocene with further diversification during the Pliocene. Diversification within the subtribe is largely consistent with key geological changes in the Neotropical landscape (Garzione *et al*., 2008) particularly from the Late Oligocene to Pliocene (~26–3 Ma) (Hoorn & Wesseling, 2010; Figs 1, 2).

*Hyposcada* diversified between mid and high elevations on the slopes of the Central Andes or Ecuador‐Colombia (Area F). The most basal members of this clade, *H. illinissa* and *H. taliata*, have much older histories than the rest of the clade, having diverged well within the Miocene. However, these species have contrasting histories, with *H. illinissa* thriving at low and mid altitudes, colonizing cis‐ and trans‐Andean habitats and western and eastern Amazonia, while *H. taliata* colonized higher elevation cis‐Andean habitats along the eastern slopes of central Peru and Ecuador. A further divergence led to the relatively rapid radiation and formation of two subclades at low altitudes during the Late Pliocene *c. *3.5 Ma, in which constituent species have a broad biogeographical distribution. The first clade contains the cis‐Andean species, *H. kena*, which is commonly found in the east Andean foothills. A further divergence at *c*. 2.5 Ma led to the widely distributed trans‐Andean, low‐mid elevation, *H. virginiana* and rarer, low elevation, *H. schausi,* which indicates that their ancestor may have crossed the Andes at the ‘Western Andean Portal’ (WAP). This corridor, which was perhaps covered by water, separated the Northern (Areas E and F) and Central Andes in northern Peru and southern Ecuador until the Middle Miocene (13–11 Ma) forming a biogeographical barrier to the dispersal of many montane species (Antonelli *et al*., 2009; Blandin & Purser, 2013). More recently, this region, which remained at a lower altitude than the neighbouring Andes, may have allowed the dispersal of some low altitude species. Furthermore, evolution of the host plant family Gesneriaceae, in particular the genera *Drymonia* and *Columnea*, which are known host plants for *H. virginiana* and other *Hyposcada* species, coincided with intense northern Andean uplift in the last 10 Ma (Perret *et al*., 2013). These plants diversified extensively, particularly at mid‐elevation in the Northern Andes and Central America. A second clade contains two geographically allopatric species, *H. anchiala* and *H. zarepha*, with the former found in the Andes and western Amazonia and the latter in the Guianas and eastern Amazonia. Their distribution is in accordance with that of Gesneriaceae, which are poorly represented in the Amazon Basin as a whole (Perret *et al*., 2013).

In contrast to *Hyposcada*,* Megoleria* originated and remained at high elevation within the Central Andes. *Megoleria* diverged from *Oleria* + *Ollantaya* at around 14.0 Ma (95% HPD: 18.7–9.4), but split into only two partially sympatric species relatively recently at 2.2 Ma (95% HPD: 4.9–0.5). *Megoleria*, similar to *Hyposcada*, feeds on the plant family Gesneriaceae (Willmott & Freitas, 2006), whose high species richness in the mountain forests of the Northern Andes should provide ample opportunity for ecological speciation driven by adaptation to alternative host plants. Yet, similar to *Hyposcada*,* Megoleria* does not seem to have taken advantage of the switch.

*Ollantaya* diversified at high elevation in the Central Andes forming *O. canilla,* and *O. olerioides* and *O. aegineta*, which separated *c*. 8.7 Ma (95% HPD: 12.2–5.7). The Central Andean endemic *O. canilla* may have been unable to cross the WAP because of the low elevation of this area at that time. The subsequent diversification of the genus following the closure of the WAP corridor led to the dispersal of *O. olerioides* and *O. aegineta* throughout the high elevation Andes possibly due to the availability of potential host plants.

*Oleria* likely originated at mid or high elevation and our findings show that four distinct clades diverged almost simultaneously within a preferred altitudinal range (De‐Silva *et al*., 2010). The most species‐rich clade, *makrena*, diverged from its sister clade, the *amalda* group at *c. 7*.9 Ma (95% HPD: 10.8–5.2) within the Central Andes. The *makrena* group radiated rapidly at mid to high elevation (> 750 m) during the Late Miocene and Pliocene between 7–3 Ma and separated into two clades. Within the first *makrena* clade, the sister species, *O. boyeri* and *O. deronda* have a disjunct distribution with the former endemic to the Guiana Shield and the latter occurring along the eastern slopes of the Central Andes (see Appendix S2), a pattern also observed in other butterfly species (Blandin & Purser, 2013). It is plausible that the demise of the Acre System from 7 to 5 Ma (Mora *et al*., 2010), the filling of the deltaic proto‐Orinoco Basin (Hoorn *et al*., 1995) and the establishment of rainforests linking western Amazonia and the Guiana Shield facilitated this eastward dispersal (Gomez *et al*., 2005). The Vaupés Arch, a palaeoarch formed by uplift of the Eastern Cordillera of the Northern Andes, may also have promoted dispersal between these regions (Hoorn *et al*., 1995).

Relationships within the second *makrena* clade were generally poorly resolved, possibly due to rapid diversification (De‐Silva *et al*., 2010), but Ecuador‐Colombia (Area F) was likely to have been important in their diversification and several distinct distribution patterns emerge. There are several examples of geographically allopatric sister species, for example, *O. vicina* and *O. quadrata* diverged *c. *5.4 Ma (95% HPD: 8.0–3.7) with the former restricted to Central America while the latter is found within the Northern Andes and along its western slopes. Although the importance of the supposed recent closure of the Panamanian Isthmus *c*. 3 Ma (Coates *et al*., 2003) in the diversification of some groups (Webb, 2006) is controversial (Baker *et al*., 2014; Montes *et al*., 2015), the landmasses were in close proximity from the Miocene and dispersal may have occurred via the Atrato Seaway (Kirby *et al*., 2008; Mullen *et al*., 2011). *O. santineza* and *O. fumata* separated *c*. 1.8 Ma (95% HPD: 2.8–0.9) and are confined to the east and west slopes of the north‐east Colombian Andes/Ecuador‐Colombia (Area F) respectively. Their divergence is coincident with the final uplift of the north‐east Colombian Andes *c*. 5–2 Ma, which was previously isolated from the proto‐Northern Andes by the Magdalena Valley (Mora *et al*., 2010). *Oleria padilla* is restricted to the eastern slopes of the Central Andes and crosses onto the western slopes of the Northern Andes in the vicinity of the WAP, while *O. makrena* dispersed along the eastern slopes of the Northern Andes.

Ecological adaptation, such as diversification on new host plants, may have been key to the formation of some sympatric sister species, including *O. athalina*/*O. fasciata* and *O. attalia/O. cyrene. Oleria victorine* has a disjunct distribution and, unlike other *Oleria*, has colonized large swathes of the Neotropics from the Venezuelan Cordilleras to the Atlantic Forest. Causes for this unusually wide distribution are unknown, but expansion in larval diet breadth could have provided opportunities for colonization of new habitats.

The less diverse *amalda* species group diversified in the Central Andes or Ecuador‐Colombia (Area F), but, in contrast to the *makrena* group, this occurred at low elevation. The *amalda* group has two allopatric subclades, an Amazonian clade that colonized the eastern slopes of the Central and Northern Andes and Amazonia, and a trans‐Andean clade that diversified along the western slopes of the Andes and Ecuador‐Colombia (Area F) before dispersal into Central America. The distribution of these clades suggests that their low altitude common ancestor crossed the Andes in the region of the WAP around 6.4 Ma (95% HPD: 8.9–4.1) before the WAP attained sufficient altitude to prevent further dispersal. This provided an alternative dispersal route and further opportunities for diversification of the group.

The *onega* group diversified in lowland forest habitats of western Amazonia mainly during the Late Miocene and Pliocene. Their diversification coincides with the disappearance of Lake Pebas and contraction of the Acre System, which are likely to have acted as dispersal barriers between the Andes and Amazonia (Wahlberg & Freitas, 2007; Antonelli *et al*., 2009; Hoorn & Wesseling, 2010). The eastward development of terra firme rainforests prompted dispersal of a relatively high number of species into eastern Amazonia, the Guiana Shield and Atlantic region (see Appendix S2). However, low diversity of the host plant, Solanaceae, within these regions (Knapp, 2002) may have precluded further diversification driven by adaptation to alternative host plants. Indeed, forest productivity is higher on nutrient‐rich western Amazonian soils, in contrast to the nutrient‐poor soils of the eastern Amazon (Hoorn *et al*., 2010). Further diversification within the monotypic *O. aegle* group in eastern Amazonia and the Guianas may also have been limited by the availability of larval host plants as has been noted with other ithomiine genera in this region (Elias *et al*., 2009). Several instances of sympatric *onega* group sister species within western Amazonia suggests that fine‐scale ecological adaptation, such as specialization on different but related host plant species (Willmott & Mallet, 2004), might have been important in the diversification of this clade.

For the Oleriina, in general, distinct Northern Andean distribution patterns have emerged from our analyses. A remarkable number of species exist within Ecuador‐Colombia (Area F), at all elevations, whereas only a few species have colonized the northeast Colombian Andes, of which only one, *Megoleria susiana,* is a strictly high elevation species; although there are a few instances of mid/high altitude species that have diversified within the last 2 Myr. Other species remain within the eastern Ecuadorian Andes or where the north‐east Colombian Andes joins the western and central Colombian Cordilleras. This pattern is consistent with the geologically recent emergence of the north‐east Colombian Cordillera and the appearance of cloud forests between 5 and 3 Ma (Kattan *et al*., 2004). There are only three instances of dispersal as far as the Venezuelan Cordilleras, consistent with their emergence as recently as 3.5 Ma (Albert *et al*., 2006). *Oleria phenomoe* is found only in this region, suggesting the extinction of it or its sister species in Northern Andean ancestral areas. Dispersal into the Atlantic region has occurred rarely, but may have been via the Mato Grosso Arch, which linked the Central Andes to the Brazilian Shield perhaps from the Late Eocene (Hoorn & Wesseling, 2010), or via continuous forest cover which previously connected eastern Amazonia and the Atlantic Forest (Costa, 2003 and references therein). These regions are now linked by interconnecting forest patches through an otherwise open landscape, which may explain the presence of *O. aquata* within the north‐eastern Caatinga and central Cerrado regions of the Brazilian Shield.

In general, changes in elevation occurred rarely in the Oleriina, as in other butterflies (Willmott *et al*., 2001; Elias *et al*., 2009; Chazot *et al*., 2014). Butterflies are likely to be limited by physiological and ecological constraints, particularly among ithomiines where mimetic species often share the same altitudinal niche (Chazot *et al*., 2014). However, exceptions are found in some groups such as the riodinid butterfly genus *Ithomiola* that radiated across an elevational gradient in the Andes (Hall, 2005).

In summary, our results reveal that the Andean orogeny instigated and had a profound influence on the diversification of the Oleriina, in agreement with the ithomiine genera *Napeogenes* and *Ithomia* (Elias *et al*., 2009), and other butterfly groups such as *Morpho* (Blandin & Purser, 2013), *Taygetis* (Matos‐Maraví *et al*., 2013) and *Lymanopoda* (Casner & Pyrcz, 2010). The evolution of the Oleriina occurred when the Central Andes had attained at least 1000‐1500 m (Garzione *et al*., 2008). It is therefore unlikely that the uplift of the Andes was a direct driver of altitudinal diversification except perhaps in those species that occur at higher elevations of more than 2000 m that were attained more recently. The Oleriina apparently dispersed throughout the Andes and into newly available cis‐ and trans‐Andean habitats when dispersal barriers such as the Acre System retreated and with the closure of the WAP. Our data support a clear role for the low lying WAP as a dispersal corridor between northern Peru and the eastern and/or western slopes of the Ecuadorian Andes. It also acted as a temporary barrier for higher elevation species. The Colombian Cordilleras Occidental and Central were probably connected to the Ecuadorian Andes a long time before the uplift of the Cordillera Oriental and this may provide an explanation for the continuity of many species ranges from the eastern Ecuadorian slopes to the Occidental and Central Cordilleras. The Andes formed a barrier causing vicariant speciation in some instances and the geologically recent uplift of the Colombian Cordillera Oriental helped to foster the rapid radiation of the *Oleria makrena* species group. Much of the Oleriina diversity remained within the Andes, but our findings suggest the Andes also acted as a source for lowland lineages.

## Biosketch

**Donna Lisa de‐Silva** is interested in understanding the patterns and processes involved in Neotropical diversification. Her work currently focuses on the ithomiine butterflies.

Author contributions: D.L.de‐S., M.E., J.M. and J.J.D. conceived the ideas; K.W. provided elevation and distribution data, D.L.de‐S. compiled and analysed the data and wrote the first draft. All co‐authors helped revise and approved the manuscript.

## Supporting information


**Appendix S1** (a) List of specimens used, GenBank accession numbers and elevation ranges for each species. (b) Museum collections from which information was obtained about elevation range and distribution. (c) Phylogenetic analysis of the Oleriina and timing of diversification – Materials and Methods, Results, References. (d) partitionfinder best substitution models.

**Appendix S2**. Distribution maps of the Oleriina species.

## References

[jbi12611-bib-0001] Albert, J.S. , Lovejoy, N.R. & Crampton, W.G.R. (2006) Miocene tectonism and the separation of the cis‐ and trans‐Andean river basins: evidence from Neotropical fishes. Journal of South American Earth Sciences, 21, 14–27.

[jbi12611-bib-0002] Antonelli, A. , Nylander, J.A.A. , Persson, C. & Sanmartín, I. (2009) Tracing the impact of the Andean uplift on Neotropical plant evolution. Proceedings of the National Academy of Sciences of the United States of America, 106, 9749–9754. 19470489 10.1073/pnas.0811421106 PMC2685738

[jbi12611-bib-0003] Ayres, J.M. & Clutton‐Brock, T.H. (1992) River boundaries and species range size in Amazonian primates. The American Naturalist, 140, 531–537. 10.1086/285427 19426056

[jbi12611-bib-0004] Baker, P.A. , Fritz, S.C. , Dick, C.W. , Eckert, A.J. , Horton, B.K. , Manzoni, S. , Ribas, C.C. , Garzione, C.N. & Battisti, D.S. (2014) The emerging field of geogenomics: constraining geological problems with genetic data. Earth‐Science Reviews, 135, 38–47.

[jbi12611-bib-0005] Blandin, P. & Purser, B. (2013) Evolution and diversification of Neotropical butterflies: insights from the biogeography and phylogeny of the genus *Morpho* Fabricius, 1807 (Nymphalidae: Morphinae), with a review of the geodynamics of South America. Tropical Lepidoptera Research, 23, 62–85.

[jbi12611-bib-0006] Casner, K.L. & Pyrcz, T.W. (2010) Patterns and timing of diversification in a tropical montane butterfly genus, *Lymanopoda* (Nymphalidae, Satyrinae). Ecography, 33, 251–259.

[jbi12611-bib-0007] Chazot, N. , Willmott, K. , Santacruz Endera, P.G. , Toporov, A. , Hill, R.I. , Jiggins, C.D. & Elias, M. (2014) Filtering by elevation and mutualistic mimicry shape the structure of Andean butterfly communities. The American Naturalist, 183, 26–39. 10.1086/674100 24334733

[jbi12611-bib-0008] Coates, A.G. , Aubry, M.P. , Berggren, W.A. , Collins, L.S. & Kunk, M. (2003) Early Neogene history of the Central American arc from Bocas del Toro, western Panama. Bulletin of the Geological Society of America, 115, 271–287.

[jbi12611-bib-0009] Condamine, F.L. , Toussaint, E.F.A. , Cotton, A.M. , Genson, G.S. , Sperling, F.A.H. & Kergoat, G.J. (2013) Fine‐scale biogeographical and temporal diversification processes of peacock swallowtails (*Papilio* subgenus *Achillides*) in the Indo‐Australian Archipelago. Cladistics, 29, 88–111. 10.1111/j.1096-0031.2012.00412.x 34814373

[jbi12611-bib-0010] Costa, L.P. (2003) The historical bridge between the Amazon and the Atlantic Forest of Brazil: a study of molecular phylogeography with small mammals. Journal of Biogeography, 30, 71–86.

[jbi12611-bib-0011] De‐Silva, D.L. , Day, J.J. , Elias, M. , Willmott, K. , Whinnett, A. & Mallet, J. (2010) Molecular phylogenetics of the neotropical butterfly subtribe Oleriina (Nymphalidae: Danainae: Ithomiini). Molecular Phylogenetics and Evolution, 55, 1032–1041. 20079859 10.1016/j.ympev.2010.01.010

[jbi12611-bib-0012] Drummond, A.J. , Suchard, M.A. , Xie, D. & Rambaut, A. (2012) Bayesian phylogenetics with BEAUti and the BEAST 1.7. Molecular Biology & Evolution, 29, 1969–1973. 22367748 10.1093/molbev/mss075 PMC3408070

[jbi12611-bib-0013] Ehlers, T.A. & Poulsen, C.J. (2009) Influence of Andean uplift on climate and paleoaltimetry estimates. Earth and Planetary Science Letters, 281, 238–248.

[jbi12611-bib-0014] Elias, M. , Gompert, Z. , Jiggins, C. & Willmott, K. (2008) Mutualistic interactions drive ecological nich convergence in a diverse butterfly community. PLoS Biology, 6, e300. 10.1371/journal.pbio.0060300 PMC2592358 19055316

[jbi12611-bib-0015] Elias, M. , Joron, M. , Willmott, K. , Silva‐Brandao, K.L. , Kaiser, V. , Arias, C.F. , Pinerez, L.M.G. , Uribe, S. , Brower, A.V.Z. , Freitas, A.V.L. & Jiggins, C.D. (2009) Out of the Andes: patterns of diversification in clearwing butterflies. Molecular Ecology, 18, 1716–1729. 19386035 10.1111/j.1365-294X.2009.04149.x

[jbi12611-bib-0016] Etienne, R.S. , Haegeman, B. , Stadler, T. , Aze, T. , Pearson, P.N. , Purvis, A. & Phillimore, A.B. (2012) Diversity‐dependence brings molecular phylogenies closer to agreement with the fossil record. Proceedings of the Royal Society B: Biological Sciences, 279, 1300–1309. 21993508 10.1098/rspb.2011.1439 PMC3282358

[jbi12611-bib-0017] Garzione, C.N. , Hoke, G.D. , Libarkin, J.C. , Withers, S. , MacFadden, B. , Eiler, J. , Ghosh, P. & Mulch, A. (2008) Rise of the Andes. Science, 320, 1304–1307. 18535236 10.1126/science.1148615

[jbi12611-bib-0018] Garzione, C.N. , Auerbach, D.J. , Jin‐Sook Smith, J. , Rosario, J.J. , Passey, B.H. , Jordan, T.E. & Eiler, J.M. (2014) Clumped isotope evidence for diachronous surface cooling of the Altiplano and pulsed surface uplift of the Central Andes. Earth and Planetary Science Letters, 393, 173–181.

[jbi12611-bib-0019] Gentry, A.H. (1982) Neotropical floristic diversity: phytogeographical connections between Central and South America, Pleistocene climatic fluctuations, or an accident of the Andean orogeny? Annals of the Missouri Botanical Garden, 69, 557–593.

[jbi12611-bib-0020] Gomez, E. , Jordan, T.E. , Allmendinger, R.W. , Cardozo, R.W. & Cardozo, N. (2005) Development of the Colombian foreland basin as a consequence of diachronous exhumation of the northern Andes. Geological Society of America Bulletin, 117, 1272–1292.

[jbi12611-bib-0021] Hall, J.P.W. (2005) Montane speciation patterns in *Ithomiola* butterflies (Lepidoptera:Riodinidae): are they consistently moving up in the world? Proceedings of the Royal Society B: Biological Sciences, 272, 2457–2466. 16271969 10.1098/rspb.2005.3254 PMC1599773

[jbi12611-bib-0022] Hoorn, C. (2006) The birth of the mighty Amazon. Scientific American, 294, 52–59. 16708488 10.1038/scientificamerican0506-52

[jbi12611-bib-0023] Hoorn C. & Wesseling P. (eds) (2010) Amazonia, landscape and species evolution: a look into the past. Wiley‐Blackwell, Chichester, UK.

[jbi12611-bib-0024] Hoorn, C. , Guerrero, J. , Sarmiento, G.A. & Lorente, M.A. (1995) Andean tectonics as a cause for changing drainage patterns in Miocene northern South America. Geology, 23, 237–240.

[jbi12611-bib-0025] Hoorn, C. , Wesseling, P. , ter Steege, H. , Bermudez, M.A. , Mora, A. , Sevink, J. , Sanmartín, I. , Sanchez‐Meseguer, A. , Anderson, C.L. , Figueiredo, J.P. , Jaramillo, C. , Riff, D. , Negri, F.R. , Hooghiemstra, H. , Lundberg, J. , Stadler, T. , Särkinen, T. & Antonelli, A. (2010) Amazonia through time: Andean uplift, climate change, landscape evolution, and biodiversity. Science, 330, 927–931. 21071659 10.1126/science.1194585

[jbi12611-bib-0026] Hoorn, C. , Mosbrugger, V. , Mulch, A. & Antonelli, A. (2013) Biodiversity from mountain building. Nature Geoscience, 6, 154.

[jbi12611-bib-0027] Hutter, C.R. , Guayasamin, J.M. & Wiens, J.J. (2013) Explaining Andean megadiversity: the evolutionary and ecological causes of glassfrog elevational richness patterns. Ecology Letters, 16, 1135–1144. 23802805 10.1111/ele.12148

[jbi12611-bib-0028] Jiggins, C.D. , Mallarino, R. , Willmott, K.R. & Bermingham, E. (2006) The phylogenetic pattern of speciation and wing pattern change in Neotropical *Ithomia* butterflies (Lepidoptera: Nymphalidae). Evolution, 60, 1454–1466. 16929662 10.1554/05-483.1

[jbi12611-bib-0029] Kattan, G.H. , Franco, P. , Rojas, V. & Morales, G. (2004) Biological diversification in a complex region: a spatial analysis of faunistic diversity and biogeography of the Andes of Colombia. Journal of Biogeography, 31, 1829–1839.

[jbi12611-bib-0030] Kennan, L. , Lamb, S.H. & Hoke, L. (1997) High‐altitude palaeosurfaces in the Bolivian Andes: evidence for late Cenozoic surface uplift. Geological Society, London, Special Publications, 120, 307–323.

[jbi12611-bib-0031] Kirby, M.X. , Jones, D.S. & MacFadden, B.J. (2008) Lower Miocene stratigraphy along the Panama Canal and its bearing on the Central American Peninsula. PLoS ONE, 3, e2791. 18665219 10.1371/journal.pone.0002791 PMC2464738

[jbi12611-bib-0032] Knapp, S. (2002) Assessing patterns of plant endemism in Neotropical uplands. Botanical Review, 68, 22–37.

[jbi12611-bib-0033] Kozak, K.M. , Wahlberg, N. , Neild, A. , Dasmahapatra, K.K. , Mallet, J. & Jiggins, C.D. (2015) Multilocus species trees show the recent adaptive radiation of the mimetic *Heliconius* butterflies. Systematic Biology, 64, 505–524. 25634098 10.1093/sysbio/syv007 PMC4395847

[jbi12611-bib-0034] Lamas, G. (2004) Ithomiinae Atlas of Neotropical Lepidoptera. Checklist: Part 4A Hesperioidea – Papilionoidea (ed. by Heppner J.B.), pp. 172–191. Association of Tropical Lepidoptera, Scientific Publishers, Gainesville, FL.

[jbi12611-bib-0035] Maddison, W. P. & Maddison, D.R. (2011) MESQUITE (a modular system for evolutionary analysis), version 2.75. Available at: http://mesquiteproject.org.

[jbi12611-bib-0036] Matos‐Maraví, P.F. , Peña, C. , Willmott, K.R. , Freitas, A.V.L. & Wahlberg, N. (2013) Systematics and evolutionary history of butterflies in the “*Taygetis* clade” (Nymphalidae: Satyrinae: Euptychiina): Towards a better understanding of Neotropical biogeography. Molecular Phylogenetics and Evolution, 66, 54–68. 23000820 10.1016/j.ympev.2012.09.005

[jbi12611-bib-0037] Merrill, R.M. , Wallbank, R.W.R. , Bull, V. , Salazar, P.C.A. , Mallet, J. , Stevens, M. & Jiggins, C.D. (2012) Disruptive ecological selection on a mating cue. Proceedings of the Royal Society B: Biological Sciences, 279, 4907–4913. 23075843 10.1098/rspb.2012.1968 PMC3497240

[jbi12611-bib-0038] Montes, C. , Cardona, A. , Jaramillo, C. , Pardo, A. , Silva, J.C. , Valencia, V. , Ayala, C. , Pérez‐Angel, L.C. , Rodriguez‐Parra, L.A. , Ramirez, V. & Niño, H. (2015) Middle Miocene closure of the Central American seaway. Science, 348, 226–229. 25859042 10.1126/science.aaa2815

[jbi12611-bib-0039] Mora, A. , Parra, M. , Strecker, M.R. , Sobel, E.R. , Hooghiemstra, H. , Torres, V. & Vallejo‐Jaramillo, J. (2008) Climatic forcing of asymmetric orogenic evolution in the Eastern Cordillera of Colombia. Bulletin of the Geological Society of America, 120, 930–949.

[jbi12611-bib-0040] Mora, A. , Baby, P. , Roddaz, M. , Parra, M. , Brusset, S. , Hermosa, W. & Espurt, N. (2010) Tectonic history of the Andes and sub‐Andean zones: implication for the development of the Amazon drainage basin Amazonia, landscape and species evolution: a look into the past (ed. by Hoorn C. and Wesselingh F.P.), pp. 38–60. Wiley‐Blackwell, Chichester.

[jbi12611-bib-0041] Mullen, S.P. , Savage, W.K. , Wahlberg, N. & Willmott, K.R. (2011) Rapid diversification and not clade age explains high diversity in Neotropical *Adelpha* butterflies. Proceedings of the Royal Society B: Biological Sciences, 278, 1777–1785. 21106589 10.1098/rspb.2010.2140 PMC3097834

[jbi12611-bib-0042] Müller, F. (1879) *Ituna* and *Thyridia*: a remarkable case of mimicry in butterflies. Transactions of the Entomological Society of London, 1879, 20–29.

[jbi12611-bib-0043] Pagel, M. , Meade, A. & Barker, D. (2004) Bayesian estimation of ancestral character states on phylogenies. Systematic Biology, 53, 673–684. 15545248 10.1080/10635150490522232

[jbi12611-bib-0044] Paradis, E. , Claude, J. & Strimmer, K. (2004) APE: analysis of phylogenetics and evolution in R language. Bioinformatics, 20, 289–290. 14734327 10.1093/bioinformatics/btg412

[jbi12611-bib-0045] Pennell, M.W. , Sarver, B.A.J. & Harmon, L.J. (2012) Trees of unusual size: biased inference of early bursts from large molecular phylogenies. PLoS Biology, 6, 483–489. 10.1371/journal.pone.0043348 PMC3434155 22957027

[jbi12611-bib-0046] Perret, M. , Chautems, A. , Araujo, A.O. & Salamin, N. (2013) Temporal and spatial origin of Gesneriaceae in the New World inferred from plastid DNA sequences. Botanical Journal of the Linnean Society, 171, 61–79.

[jbi12611-bib-0047] Pybus, O.G. & Harvey, P.H. (2000) Testing macro‐evolutionary models using incomplete molecular phylogenies. Proceedings of the Royal Society B: Biological Sciences, 267, 2267–2272. 11413642 10.1098/rspb.2000.1278 PMC1690817

[jbi12611-bib-0048] R Core Team . (2015) R: A language and environment for statistical computing, version 3.2.0. R Foundation for Statistical Computing, Vienna, Austria Available at: https://www.R-project.org.

[jbi12611-bib-0049] Rabosky, D.L. (2006) LASER: a maximum likelihood toolkit for detecting temporal shifts in diversification rates from molecular phylogenies. Evolutionary Bioinformatics, 2, 247–250. PMC2674670 19455217

[jbi12611-bib-0050] Rabosky, D.L. & Lovette, I.J. (2008) Density‐dependent diversification in North American wood warblers. Proceedings of the Royal Society B: Biological Sciences, 275, 2363–2371. 18611849 10.1098/rspb.2008.0630 PMC2603228

[jbi12611-bib-0051] Ree, R.H. & Smith, S.A. (2008) Maximum likelihood inference of geographic range evolution by dispersal, local extinction, and cladogenesis. Systematic Biology, 57, 4–14. 18253896 10.1080/10635150701883881

[jbi12611-bib-0052] Rosser, N. , Phillimore, A.B. , Huertas, B. , Willmott, K.R. & Mallet, J. (2012) Testing historical explanations for gradients in species richness in heliconiine butterflies of tropical America. Biological Journal of the Linnean Society, 105, 479–497.

[jbi12611-bib-0053] Rull, V. (2013) Palaeoclimates and Amazon biodiversity. Journal of Biogeography, 40, 1413–1414.

[jbi12611-bib-0054] Santos, J.C. , Coloma, L.A. , Summers, K. , Caldwell, J.P. , Ree, R. & Cannatella, D.C. (2009) Amazonian amphibian diversity is primarily derived from Late Miocene Andean lineages. PLoS Biology, 7, 448–461. 10.1371/journal.pbio.1000056 PMC2653552 19278298

[jbi12611-bib-0055] Schulter, D. (2000) The ecology of adaptive radiation. Oxford University Press, Oxford.

[jbi12611-bib-0056] Sébrier, M. , Mercier, J.L. , Macharé, J. , Bonnot, D. , Cabrera, J. & Blanc, J.L. (1988) The state of stress in an overriding plate situated above a flat slab: the Andes of central Peru. Tectonics, 7, 895–928.

[jbi12611-bib-0057] Tuomisto, H. , Ruokolainen, K. , Kalliola, K. , Linna, R. , Danjoy, W. & Rodriguez, Z. (1995) Dissecting Amazonian biodiversity. Science, 269, 63–66. 17787706 10.1126/science.269.5220.63

[jbi12611-bib-0058] Turchetto‐Zolet, A.C. , Pinheiro, F. , Salbueiro, F. & Palma‐Silva, C. (2013) Phylogeographical patterns shed light on evolutionary process in South America. Molecular Ecology, 22, 1193–1213. 23279129 10.1111/mec.12164

[jbi12611-bib-0059] Wahlberg, N. & Freitas, A.V.L. (2007) Colonization of and radiation in South America by butterflies in the subtribe Phyciodina (Lepidoptera: Nymphalidae). Molecular Phylogenetics and Evolution, 44, 1257–1272. 17560801 10.1016/j.ympev.2007.04.012

[jbi12611-bib-0060] Wahlberg, N. , Leneveu, J. , Kodandaramaiah, U. , Peña, C. , Nylin, S. , Freitas, A.V.L. & Brower, A.V.Z. (2009) Nymphalid butterflies diversify following near demise at the Cretaceous/Tertiary boundary. Proceedings of the Royal Society B: Biological Sciences, 276, 4295–4302. 19793750 10.1098/rspb.2009.1303 PMC2817107

[jbi12611-bib-0061] Webb, S.D. (2006) The Great American Biota Interchange: patterns and processes. Annals of the Missouri Botanical Garden, 93, 245–257.

[jbi12611-bib-0062] Wesselingh, F.P. , Räsänen, M.E. , Irion, G.E. , Vonhof, H.B. , Kaandorp, R. , Renema, W. , Romero Pittman, L. & Gingras, M. (2002) Lake Pebas: a palaeoecological reconstruction of a Miocene, long‐lived lake complex in western Amazonia. Cainozoic Research, 1, 35–81.

[jbi12611-bib-0063] Whipple, K.X. & Gasparini, N.M. (2014) Tectonic control of topography, rainfall patterns, and erosion during rapid post‐12 Ma uplift of the Bolivian Andes. Lithosphere, 6, 251–268.

[jbi12611-bib-0064] Willmott, K.R. & Freitas, A.V.L. (2006) Higher‐level phylogeny of the Ithomiinae (Lepidoptera: Nymphalidae): classification, patterns of larval hostplant colonization and diversification. Cladistics, 22, 297–368. 10.1111/j.1096-0031.2006.00108.x 34892866

[jbi12611-bib-0065] Willmott, K.R. & Mallet, J. (2004) Correlations between adult mimicry and larval hostplants in ithomiine butterflies. Proceedings of the Royal Society B: Biological Sciences, 271, S266–S269. 15503990 10.1098/rsbl.2004.0184 PMC1810062

[jbi12611-bib-0066] Willmott, K.R. , Hall, J.P.W. & Lamas, G. (2001) Systematics of *Hypanartia* (Lepidoptera: Nymphalidae: Nymphalinae), with a test for geographical speciation mechanisms in the Andes. Systematic Entomology, 26, 369–399.

