# Supplementary material for: Diversification of clearwing butterflies with the rise of the Andes
Source: J Biogeogr. 2015 Sep 24;43(1):44–58. doi: 10.1111/jbi.12611 (PMC4973677; doi:10.1111/jbi.12611)
Supplement: Supplementary file 2 — Appendix S2. Distribution maps of the Oleriina species. [file JBI-43-44-s002.pdf]

Journal of Biogeography

SUPPORTING INFORMATION

Diversification of clearwing butterflies with the rise of the Andes

Donna Lisa De-Silva, Marianne Elias, Keith Willmott, James Mallet and Julia J. Day

Appendix S2 Distribution maps of the Oleriina species. Localities were recorded from extensive examination of museum collections and records of collaborators. The distribution of the Oleriina is well known in all biogeographic areas with the exception of central Amazonia[illegible]ich forms part of western and eastern Amazonia (Areas H and I, see Fig.2.[illegible]

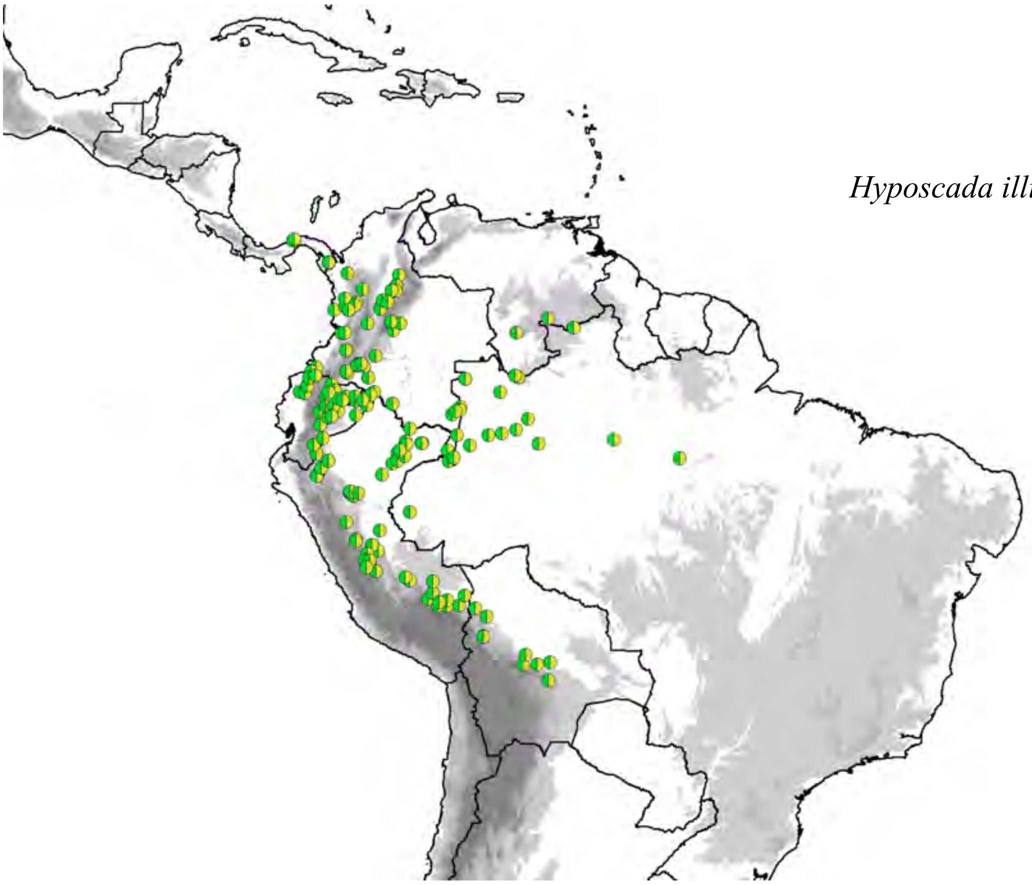

Hyposcada illinissa

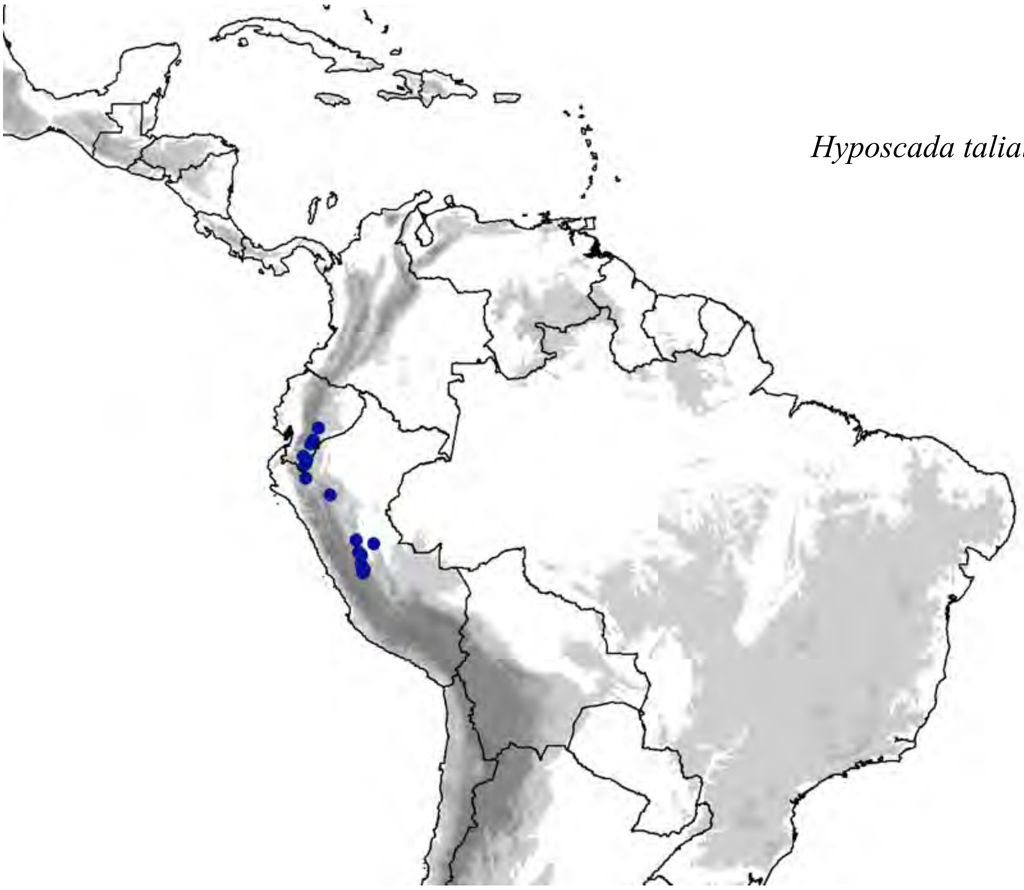

Hyposcada taliata

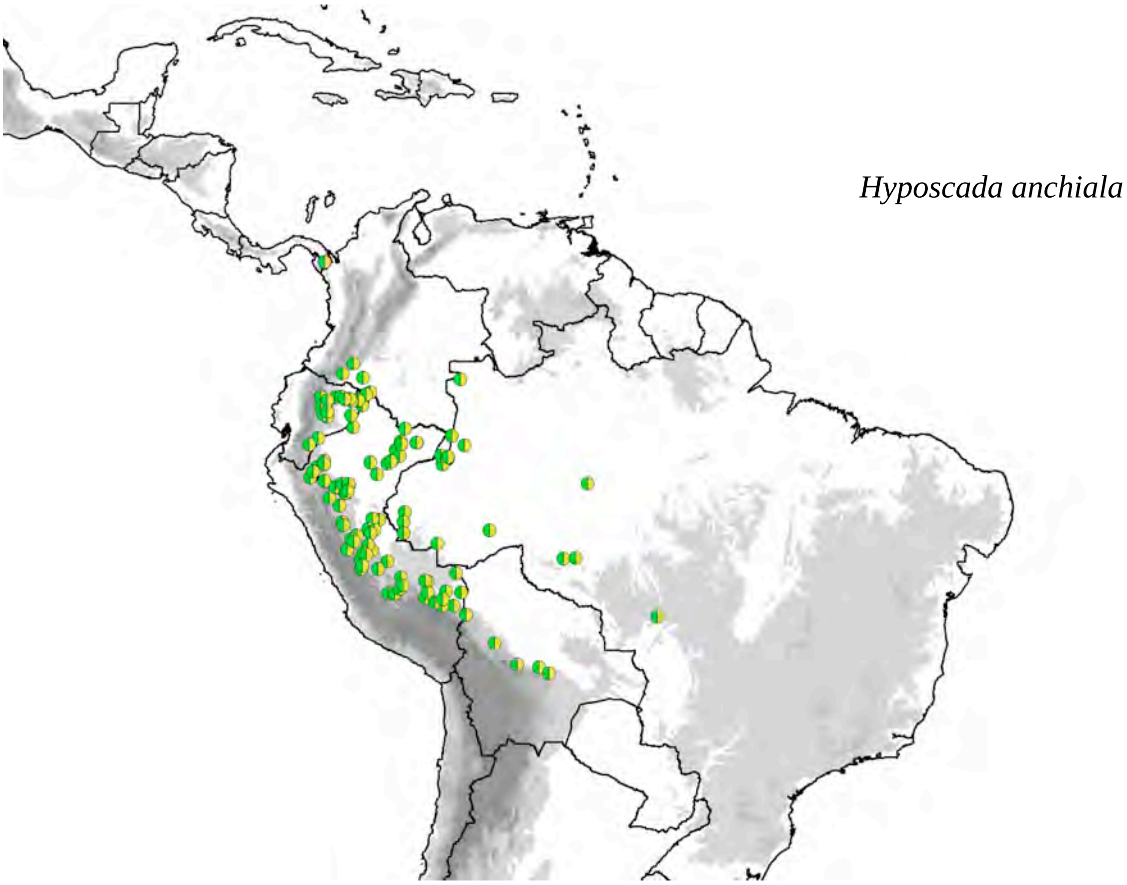
Hyposcada anchiala

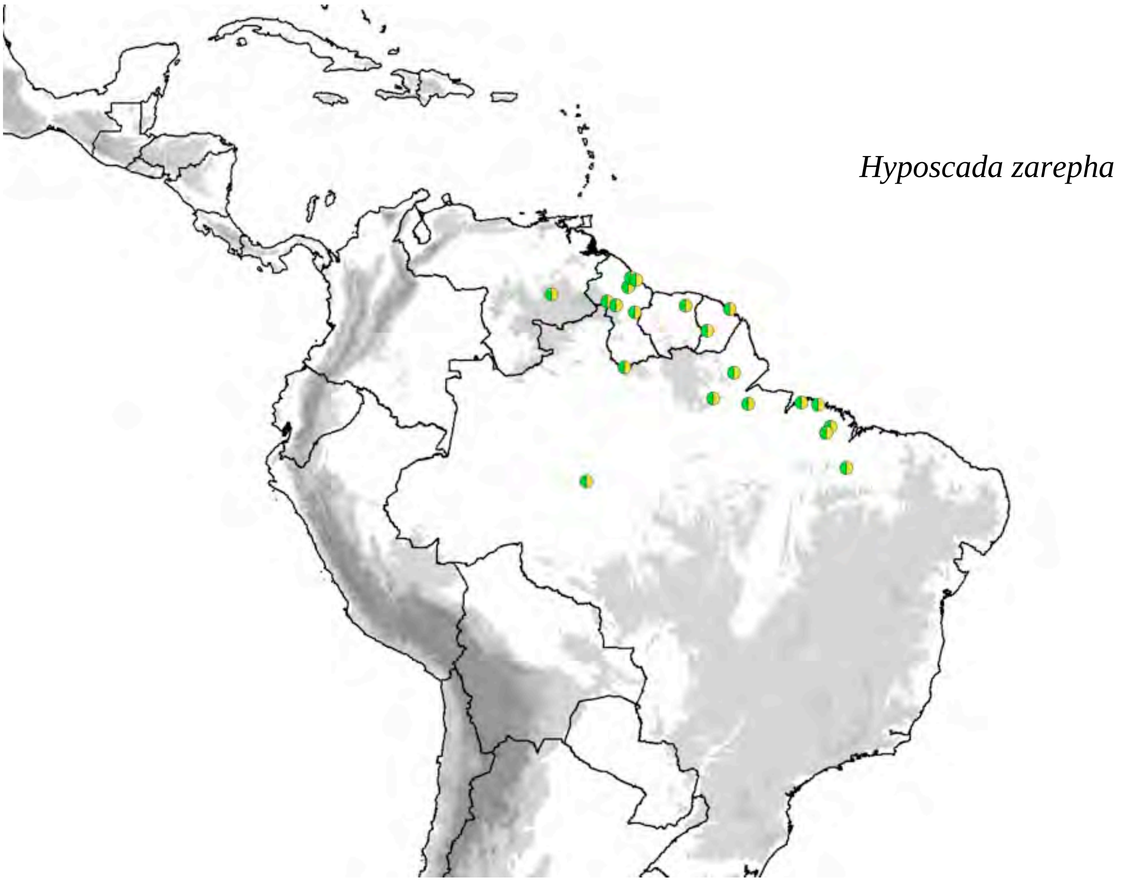
Hyposcada zarepha

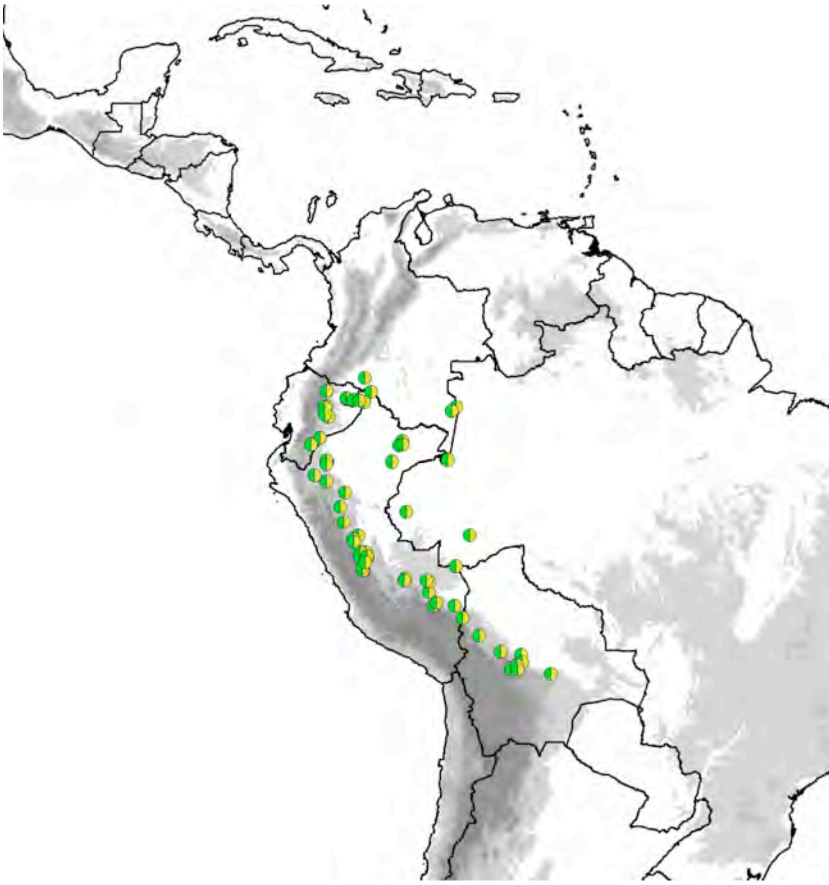

Hyposcada kena

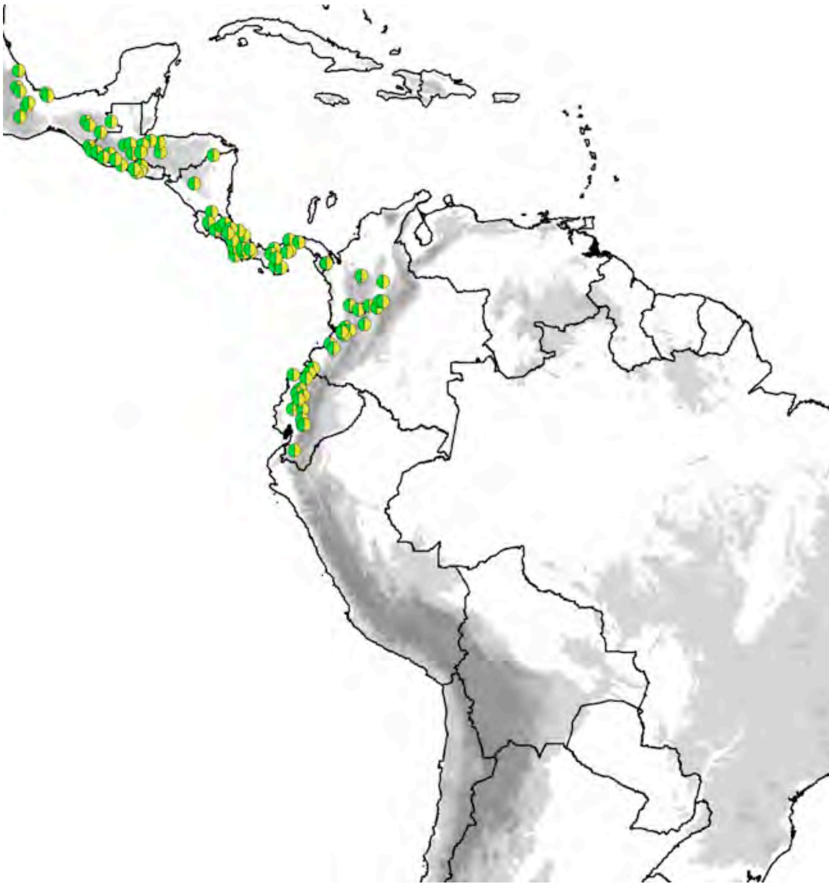

Hyposcada virginiana

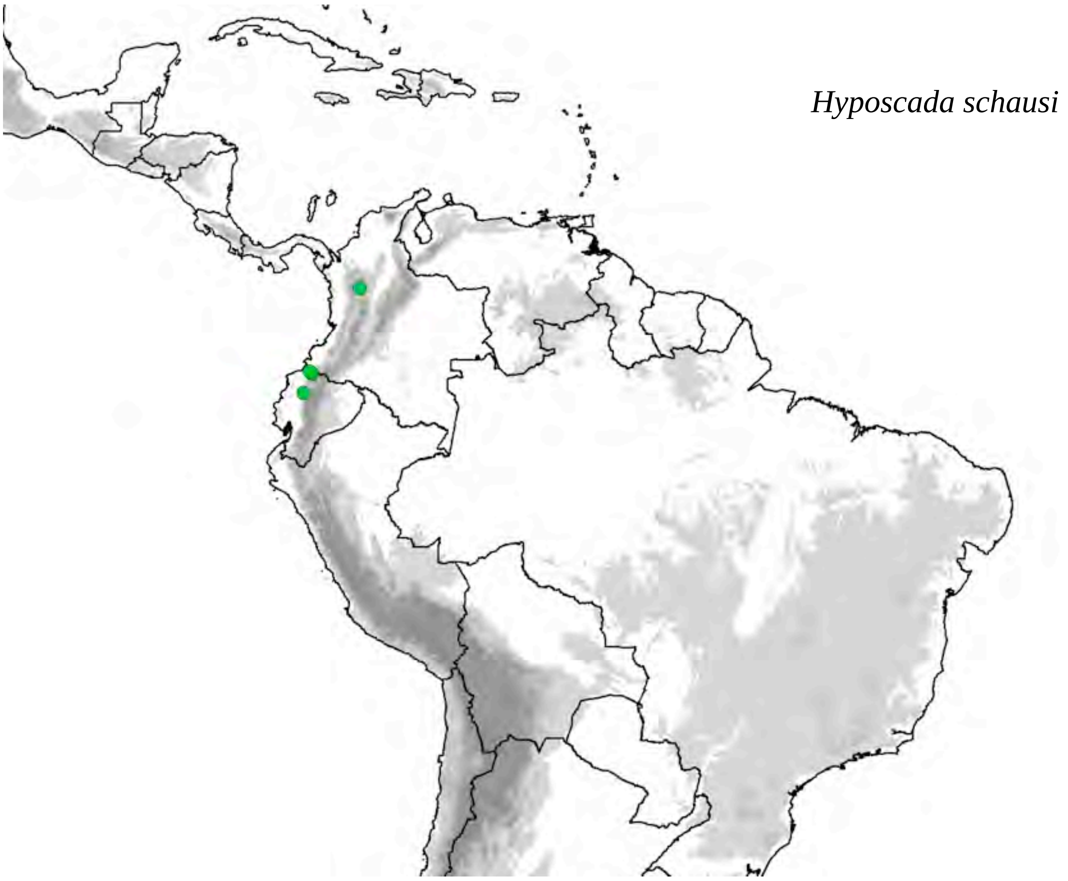
Hyposcada schausi

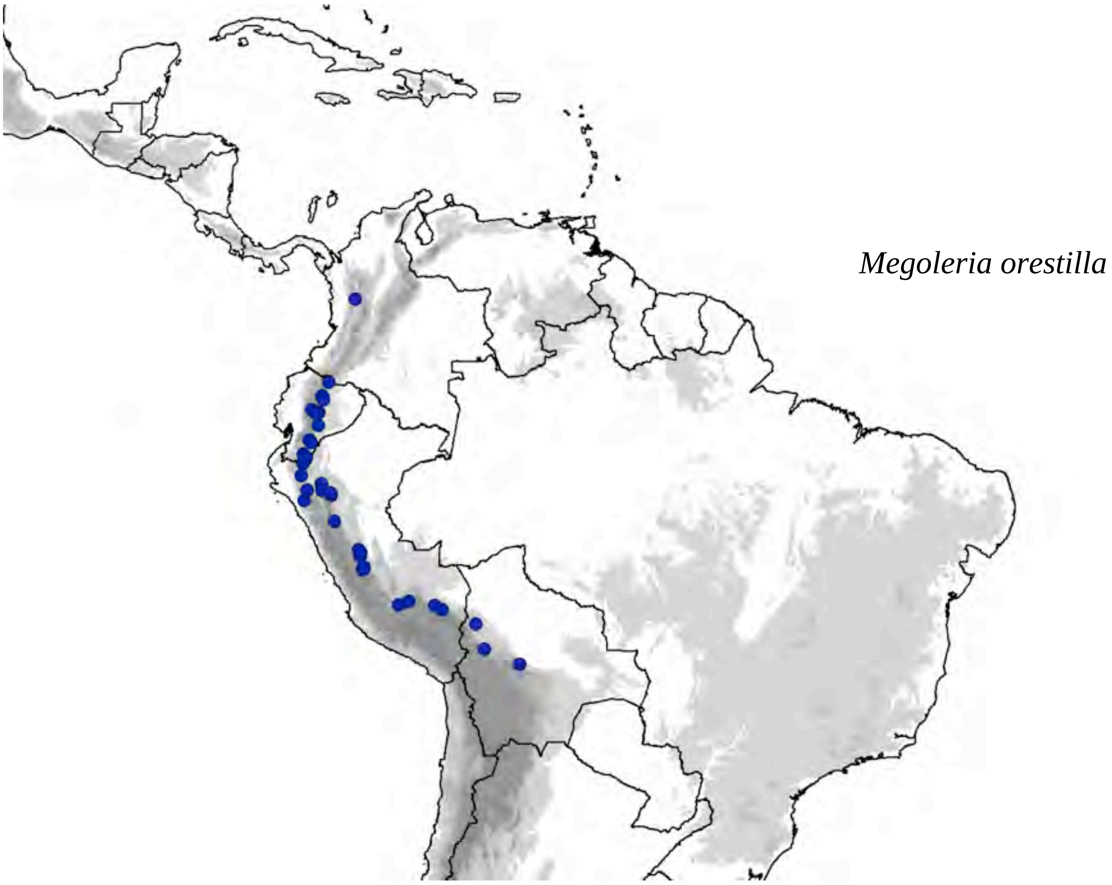
Megoleria orestilla

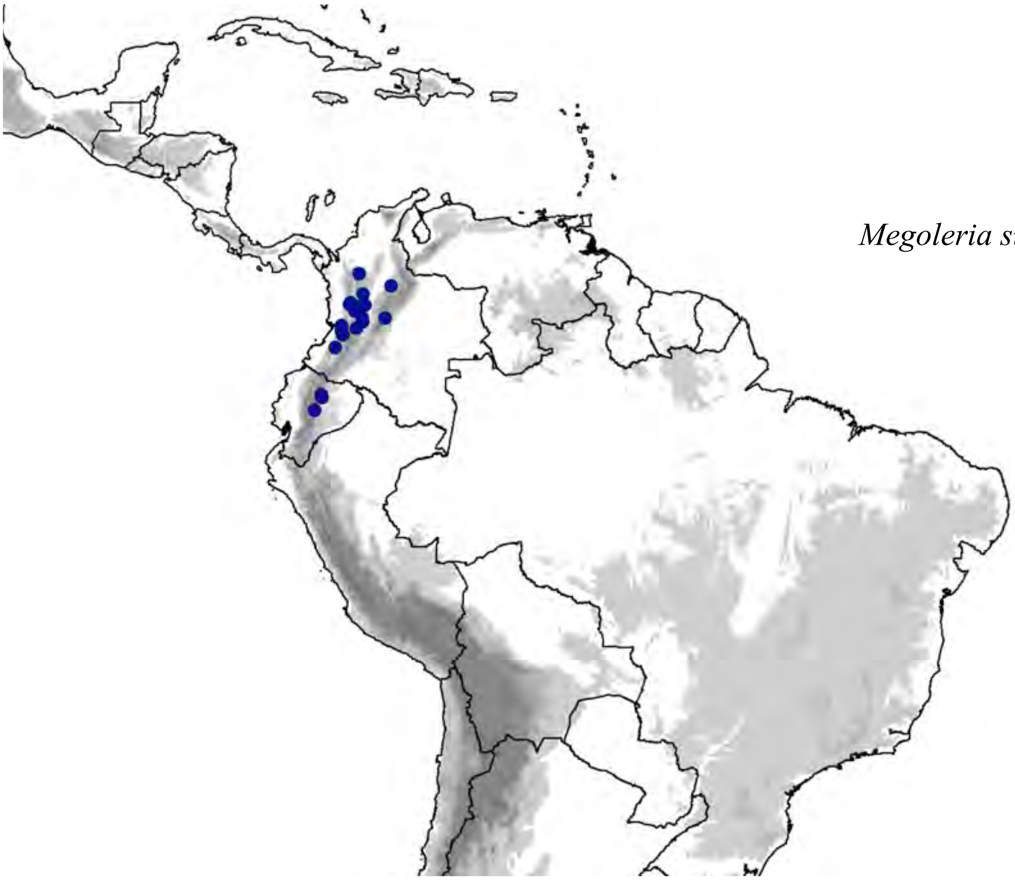

Megoleria susiana

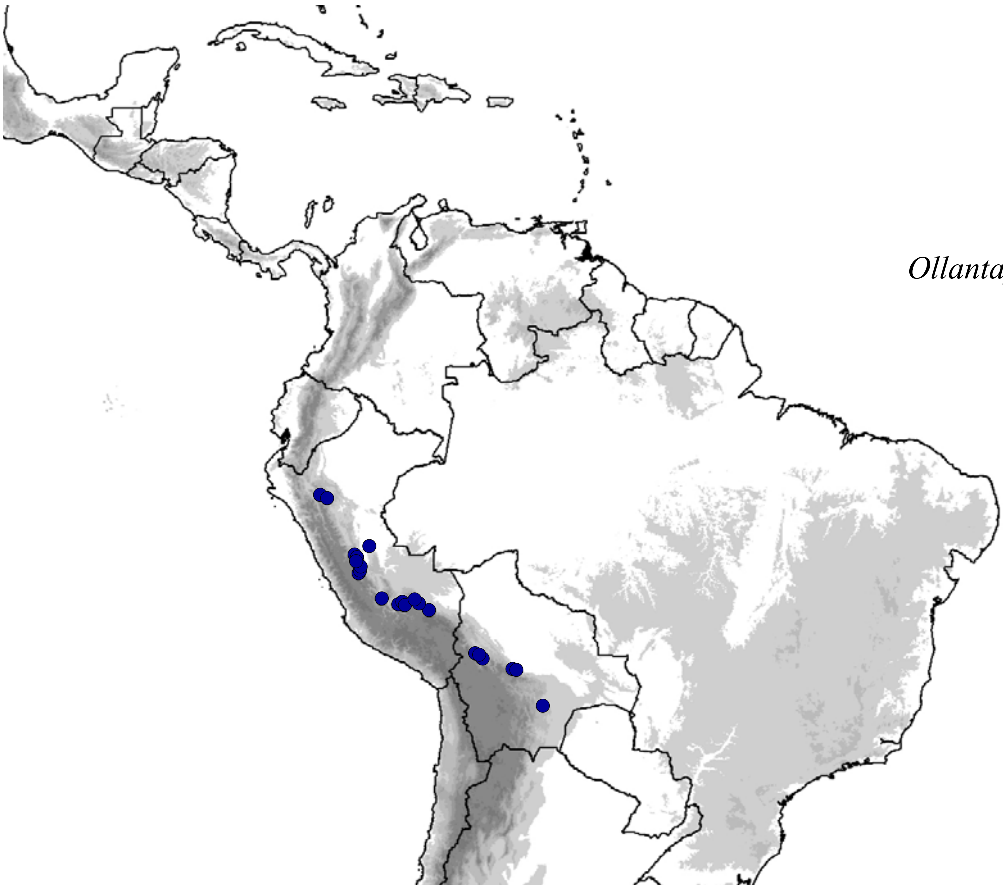

Ollantaya canilla

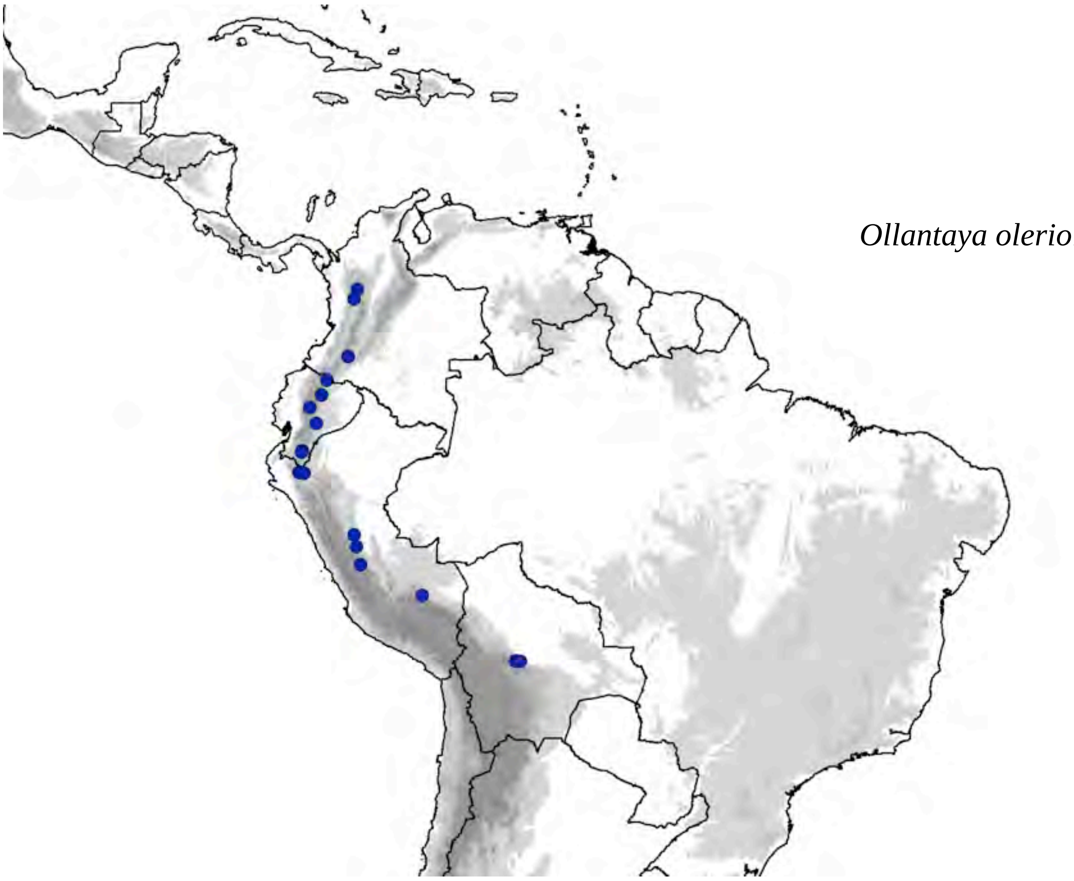

Ollantaya olerioides

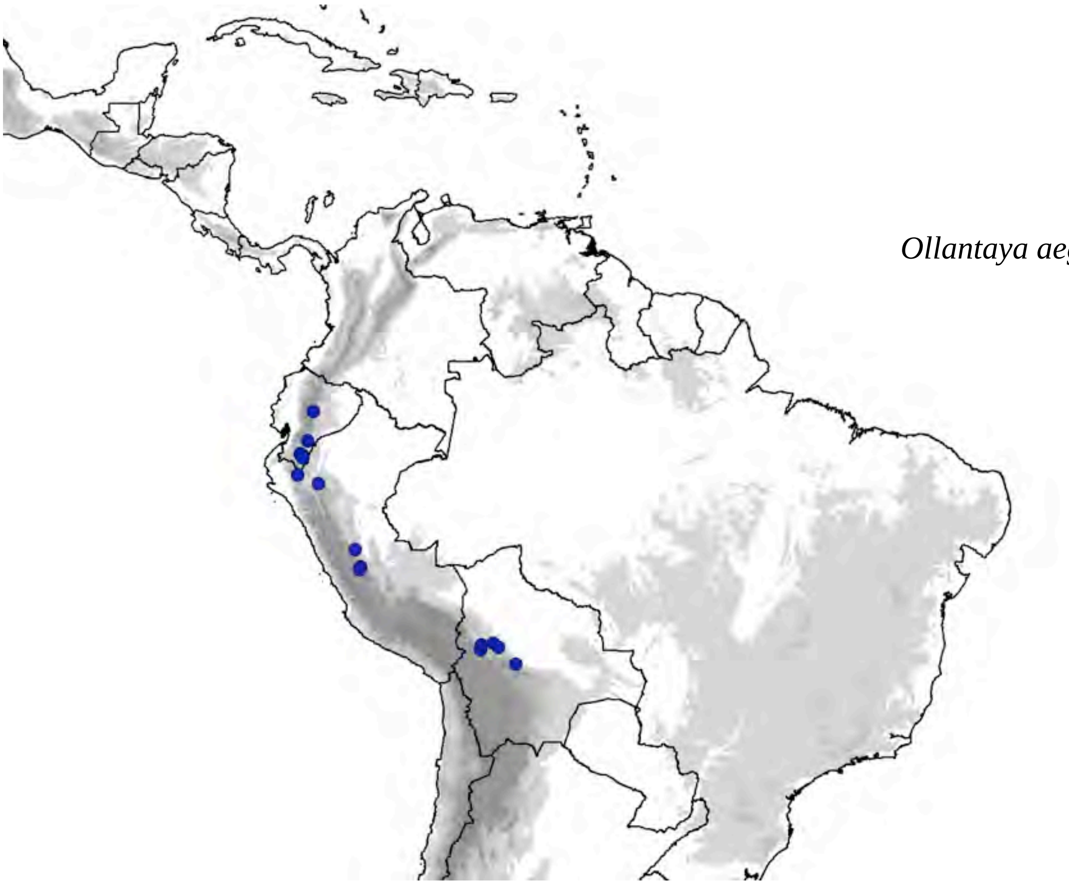

Ollantaya aegineta

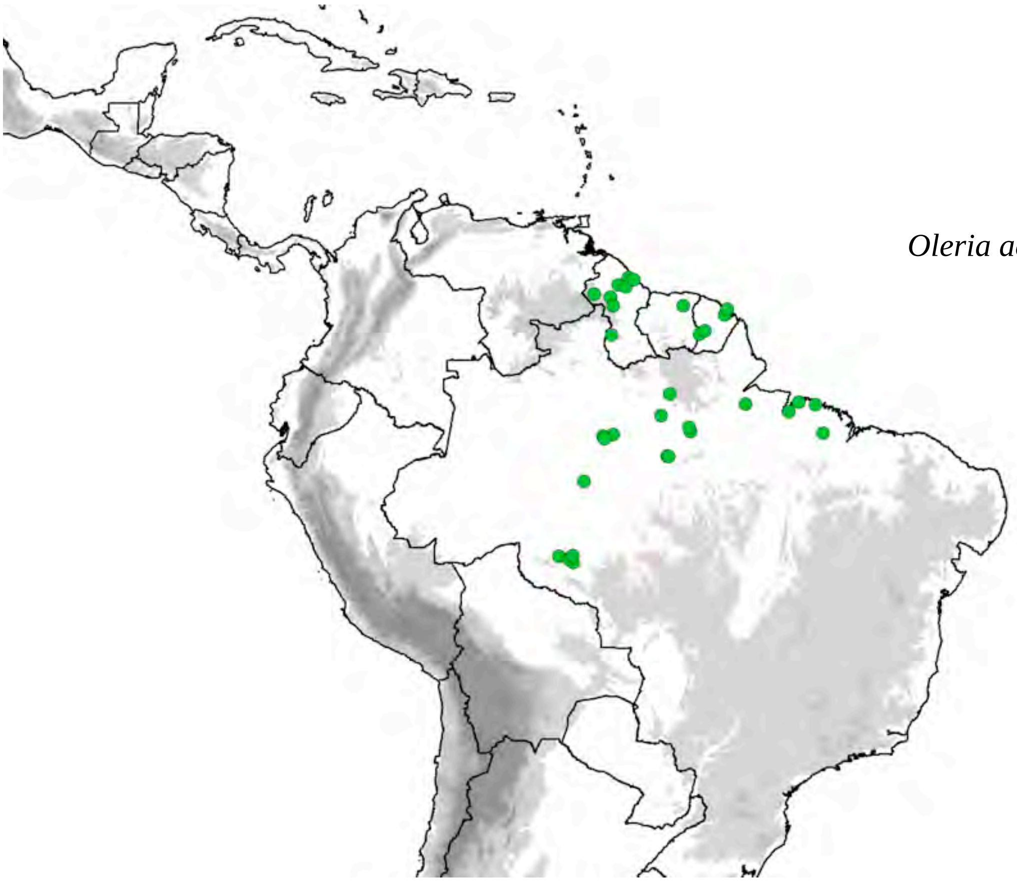

Oleria aegle

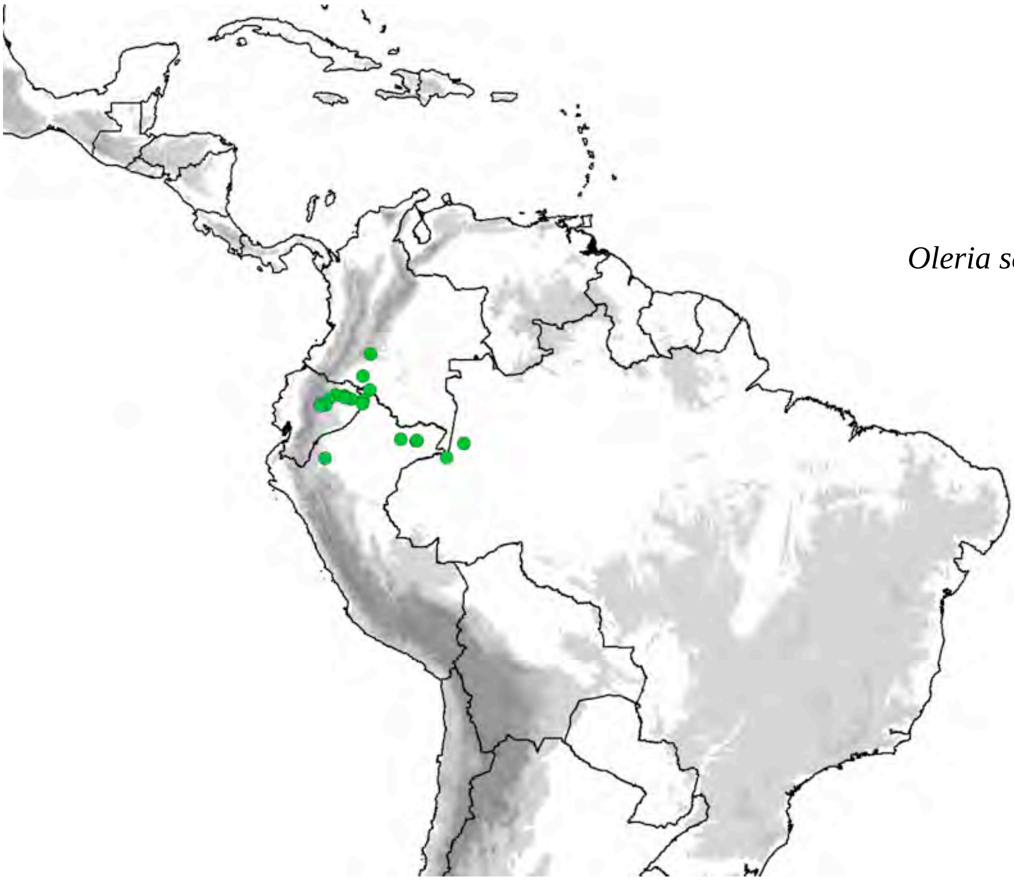

Oleria sexmaculata

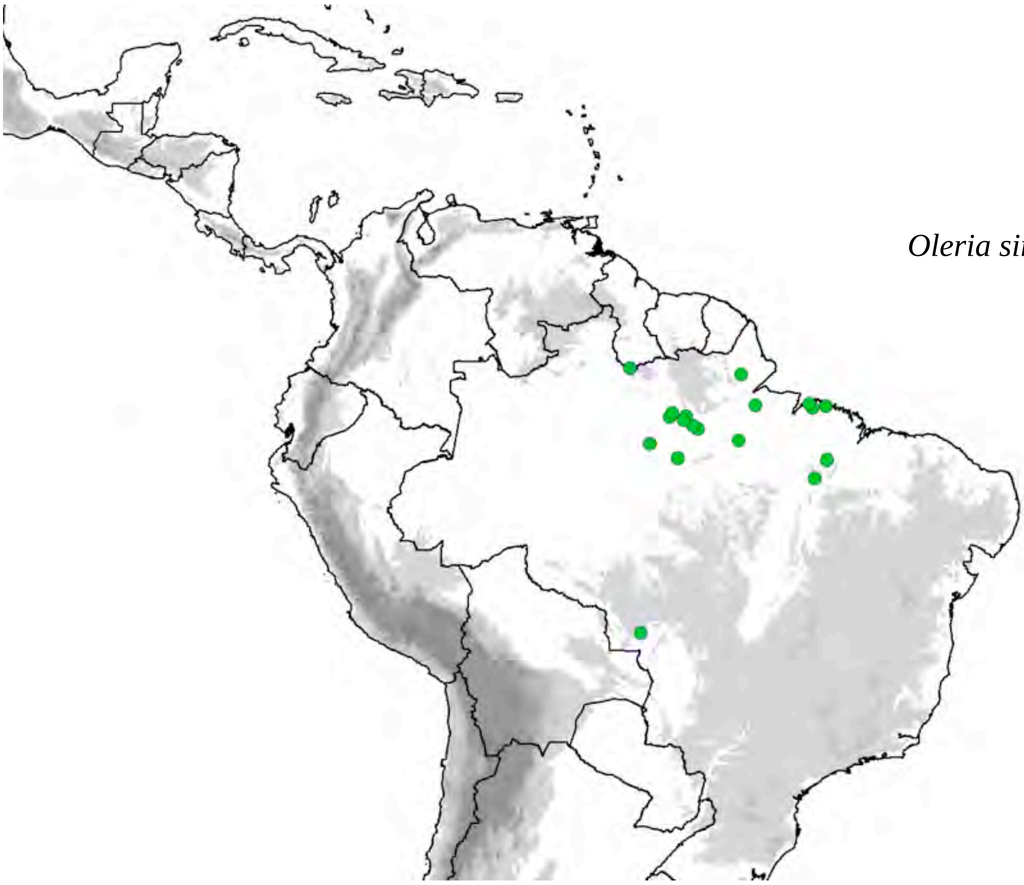

Oleria similigena

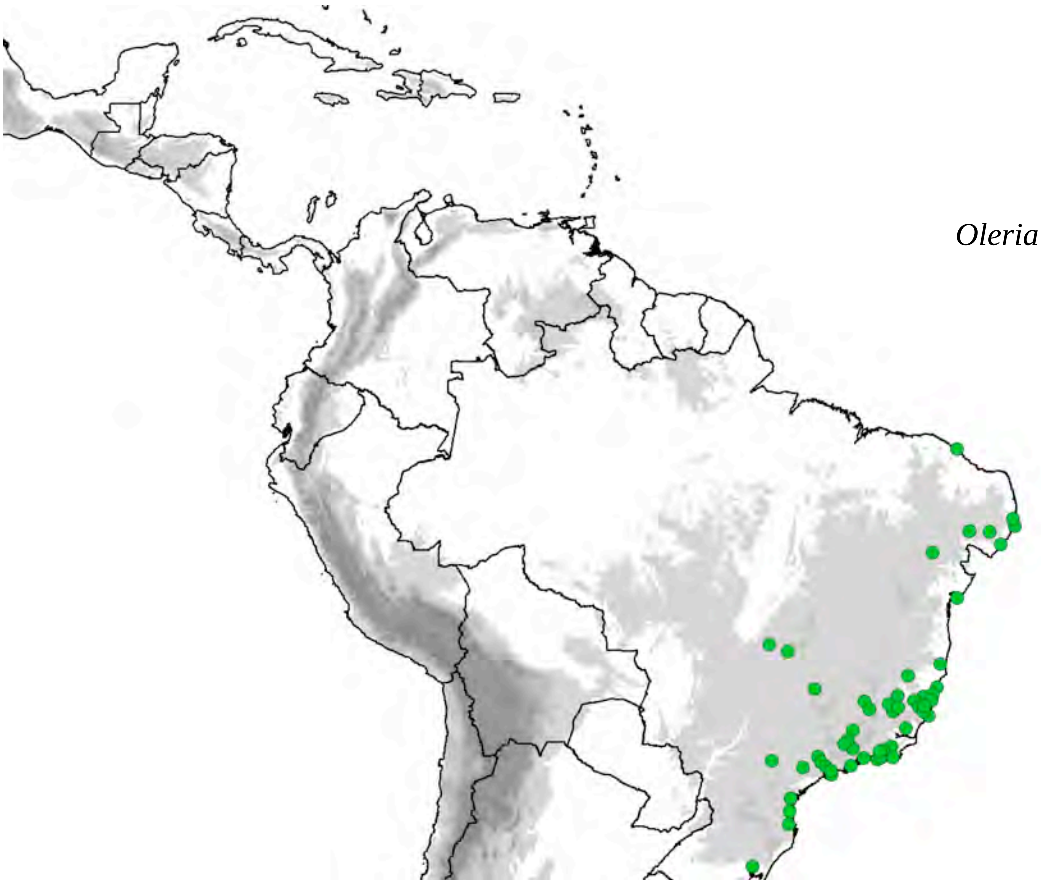

Oleria aquata

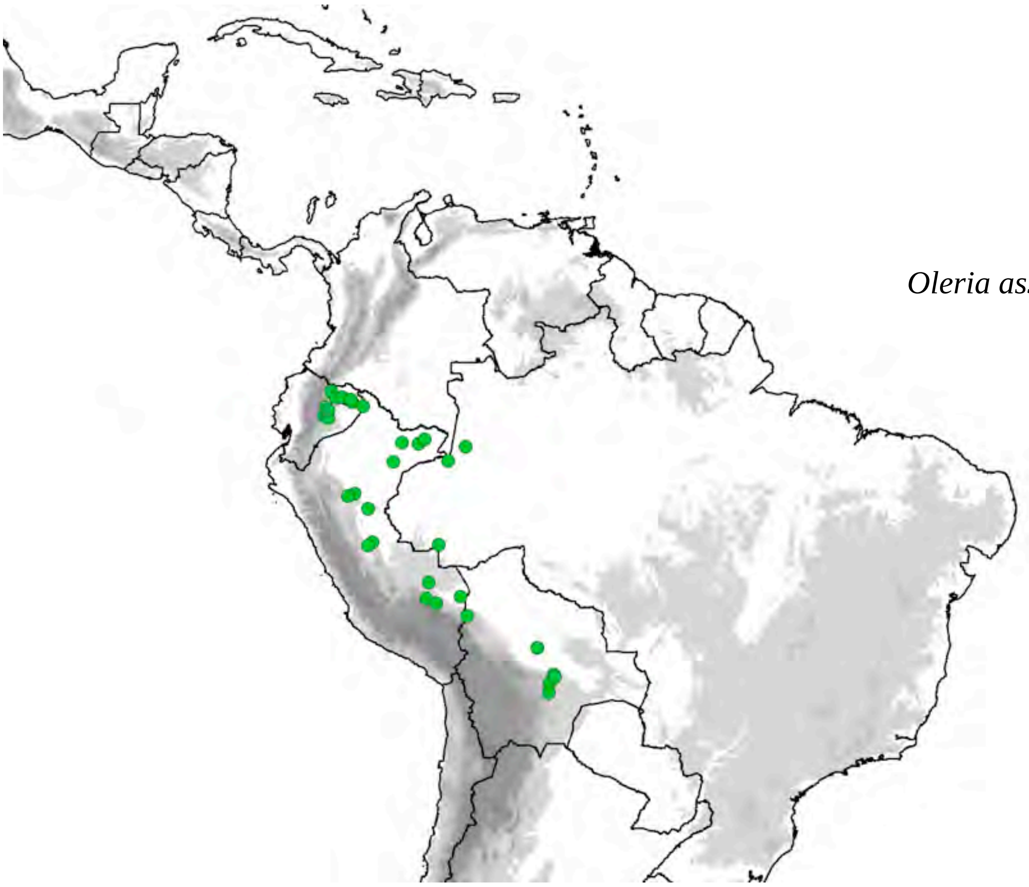

Oleria assimilis

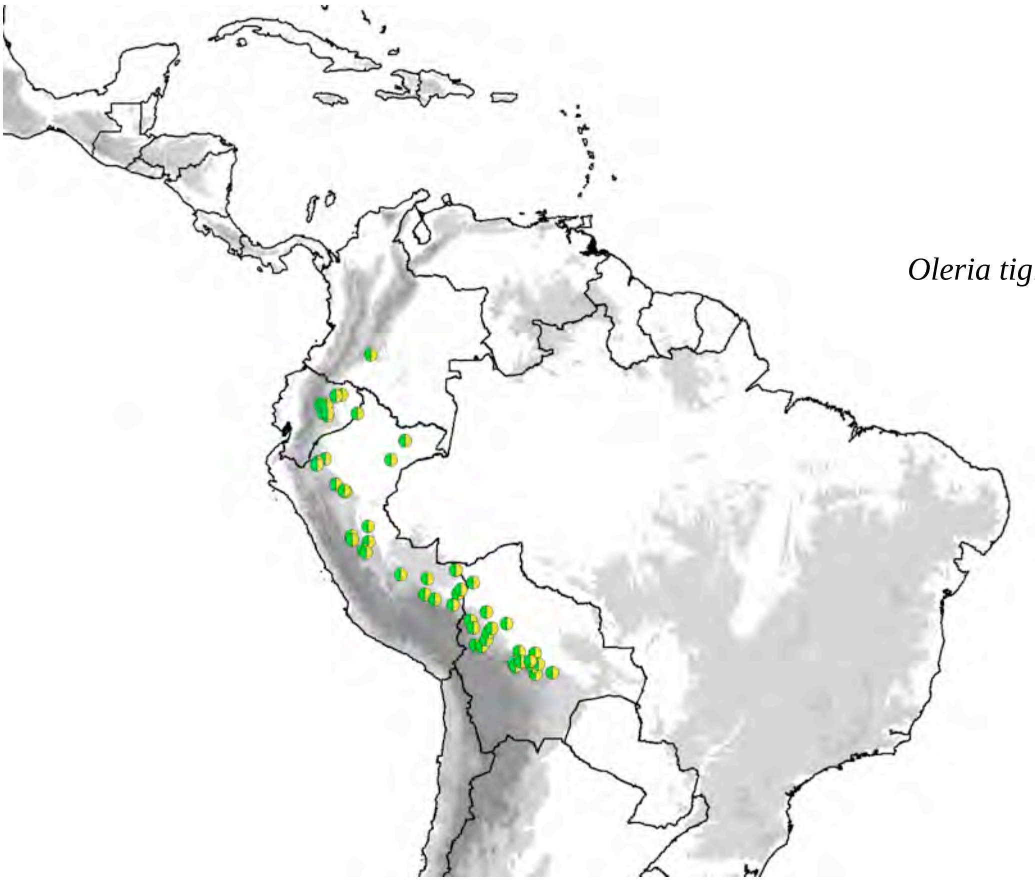

Oleria tigilla

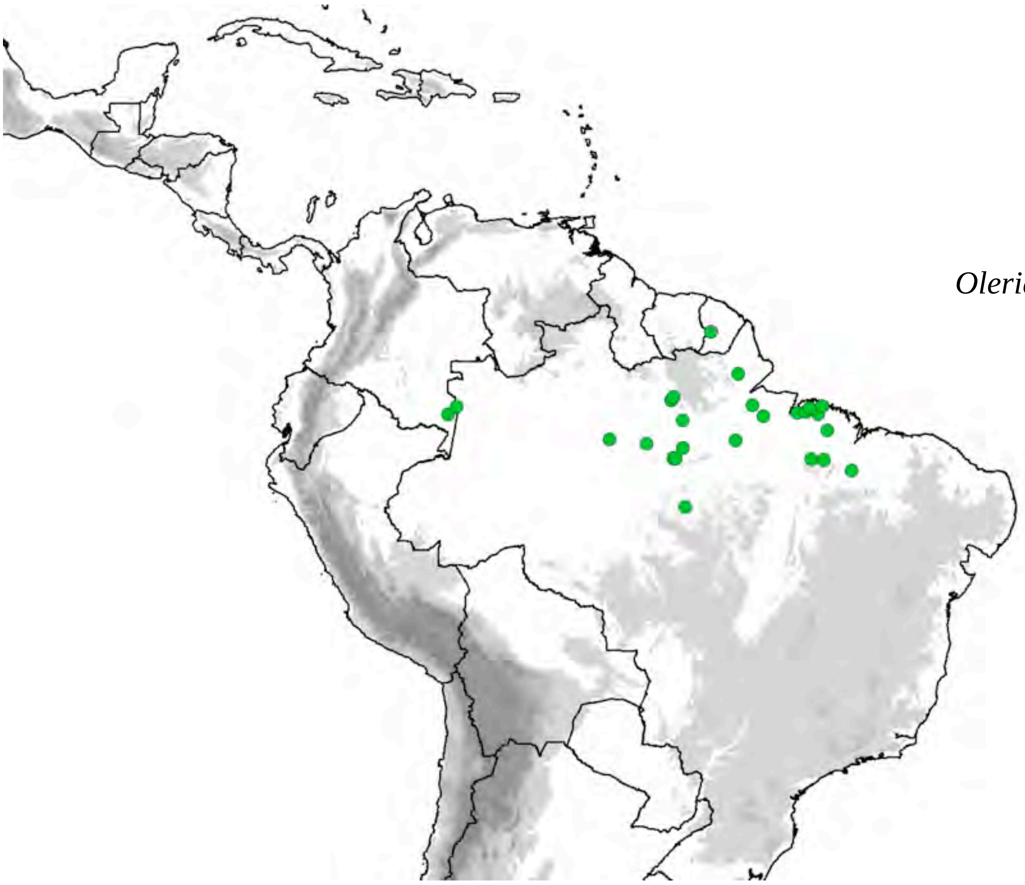

Oleria antaxis

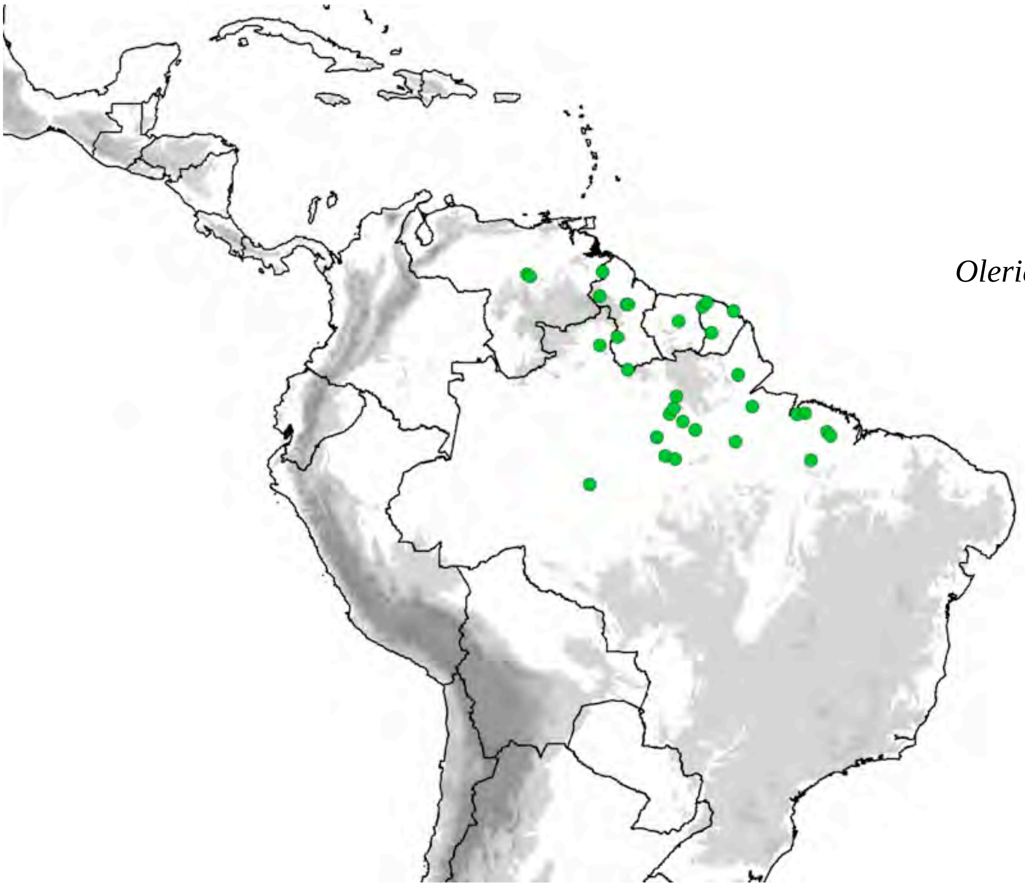

Oleria flora

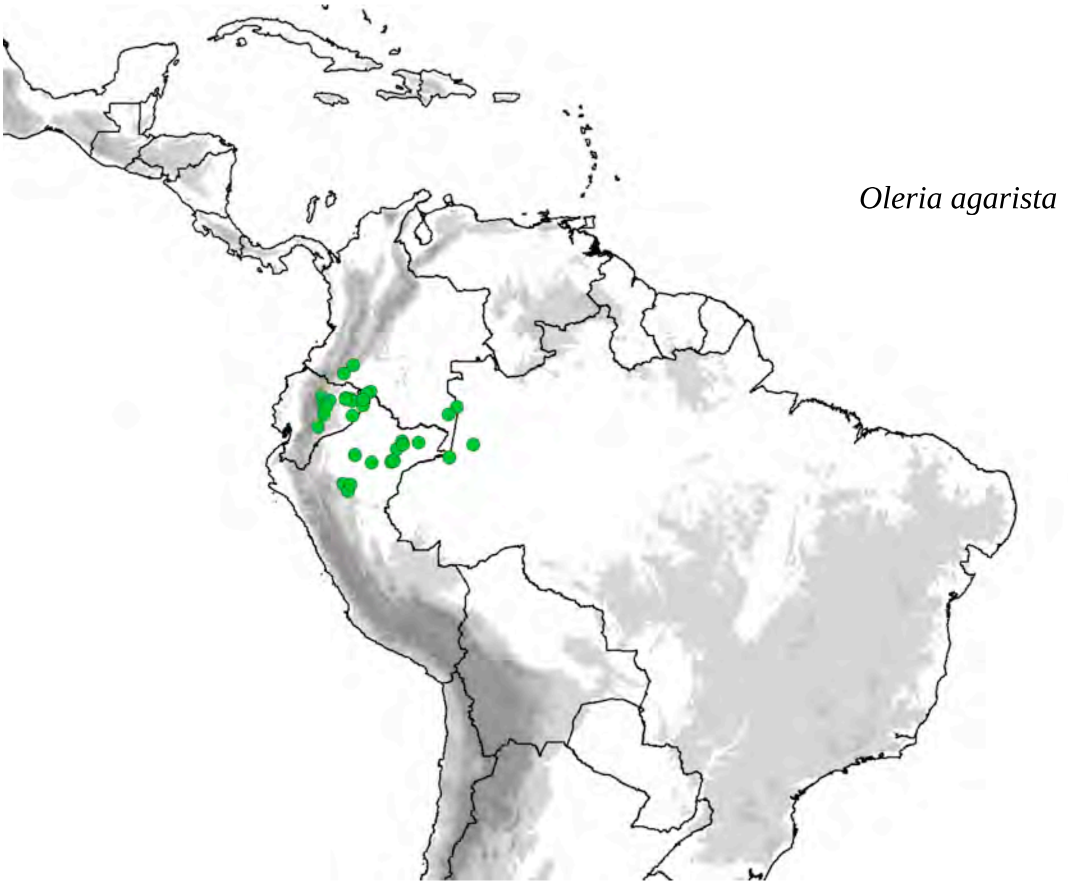
Oleria agarista

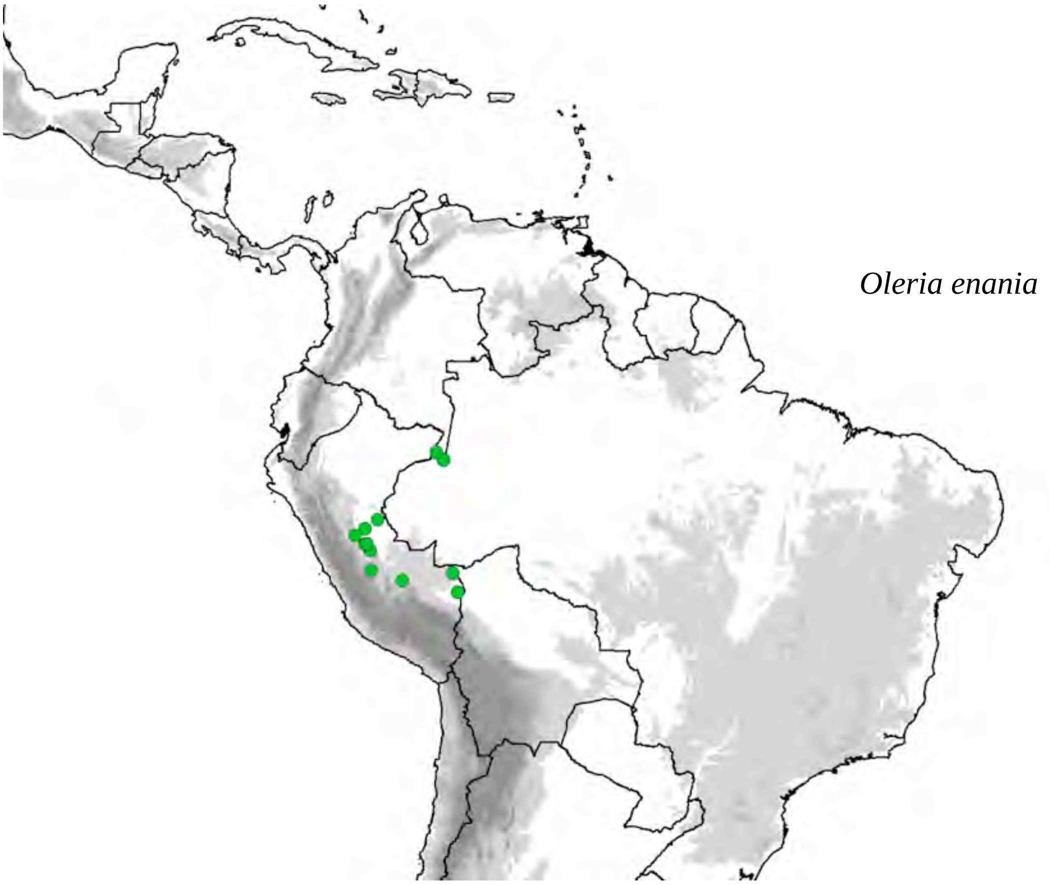
Oleria enania

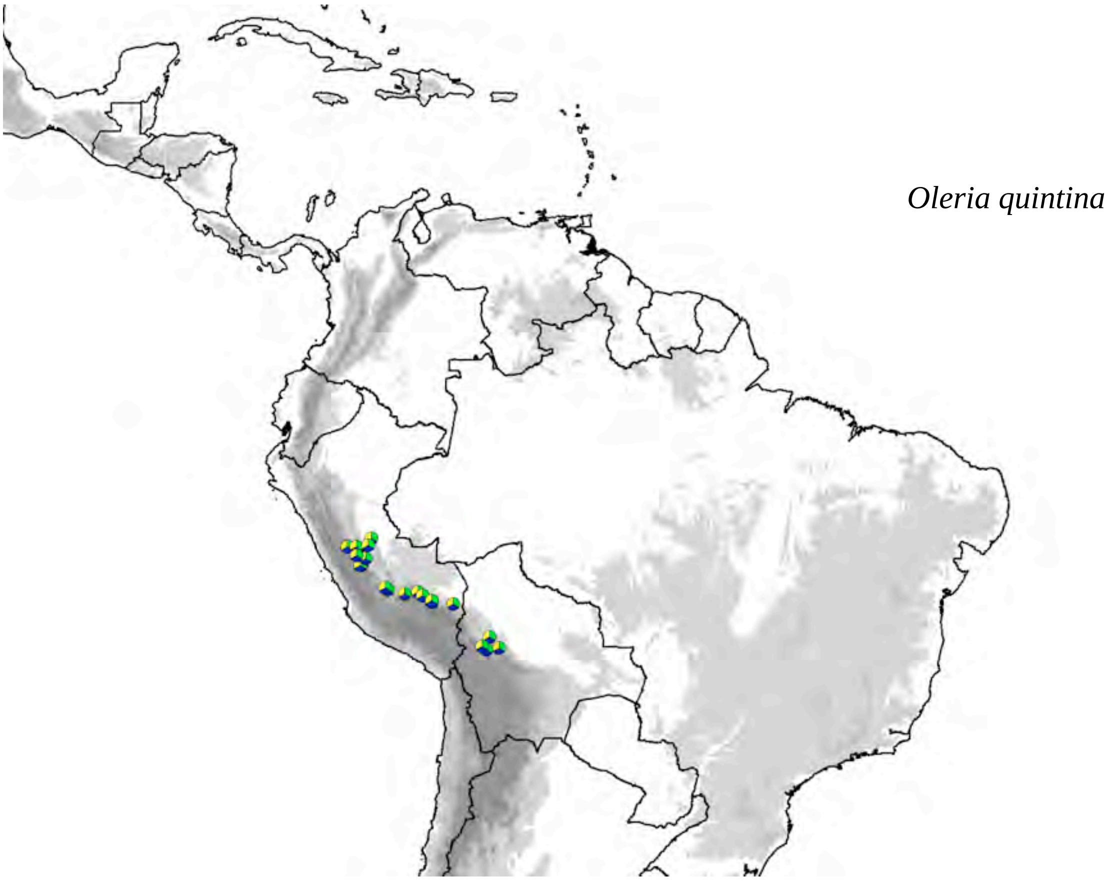
Oleria quintina

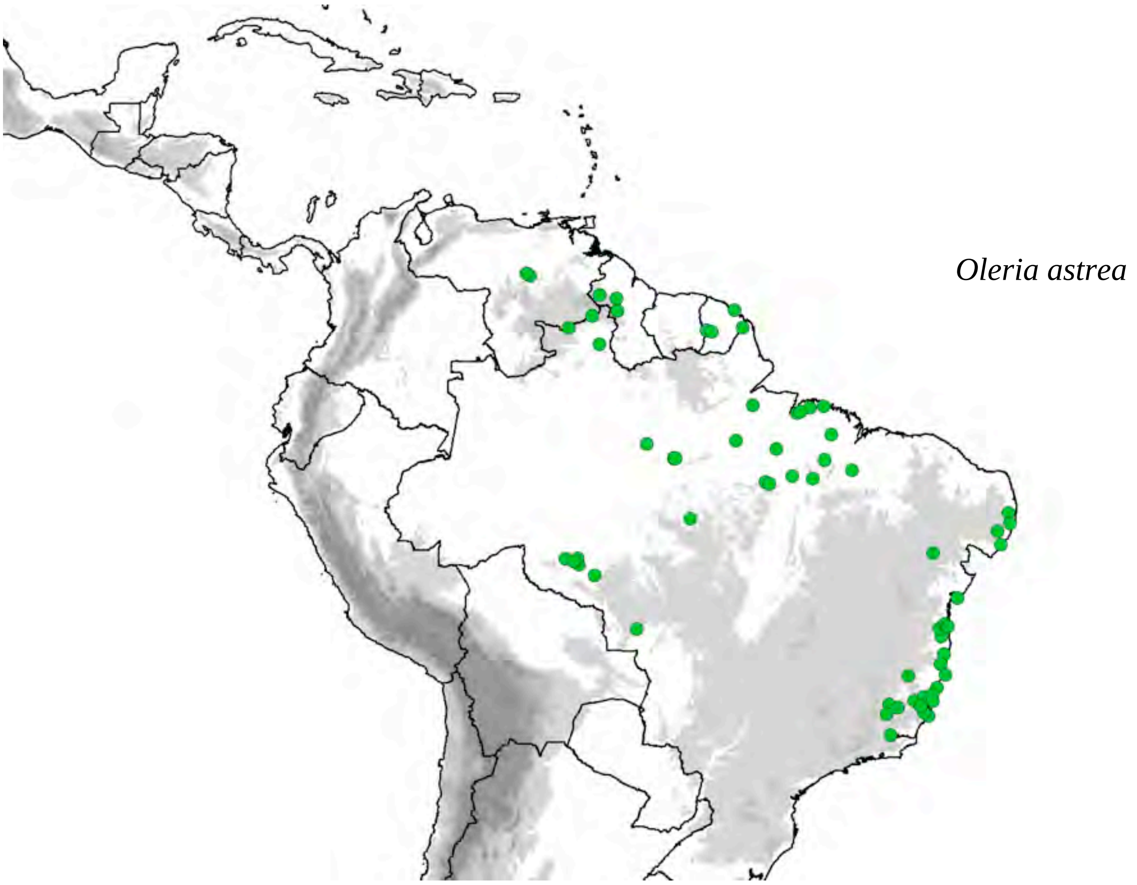
Oleria astrea

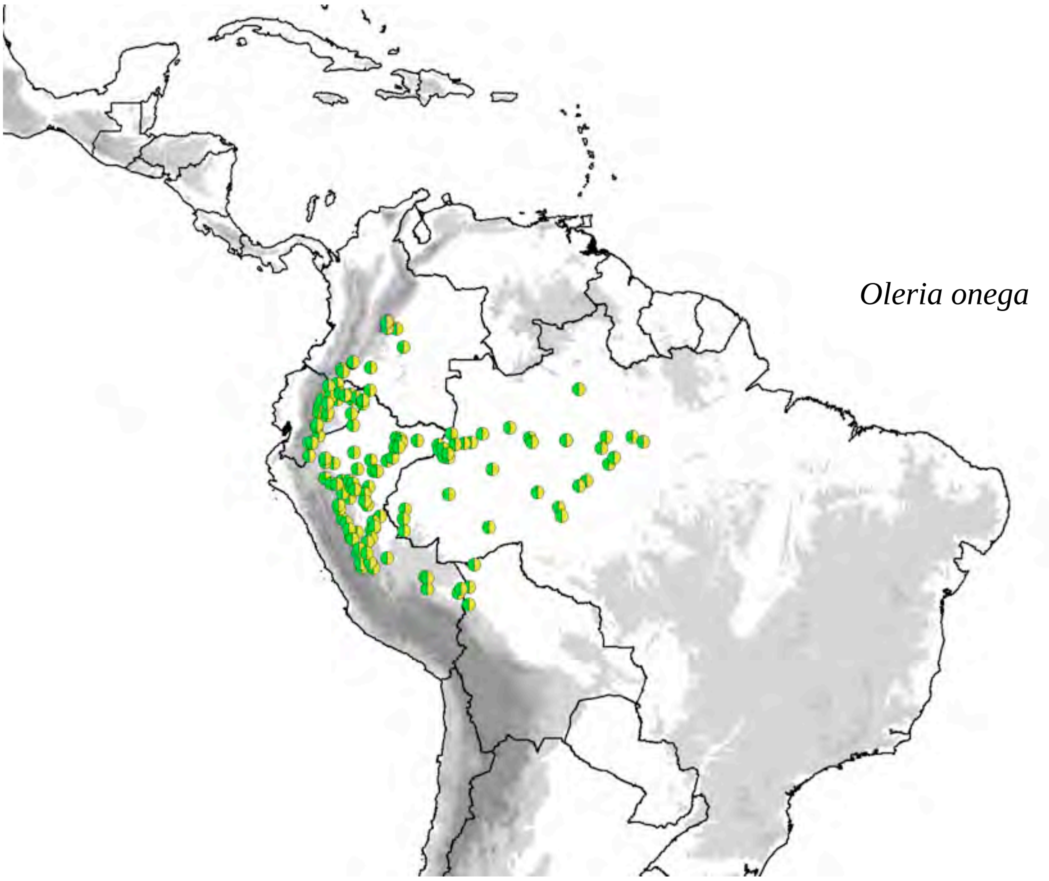

Oleria onega

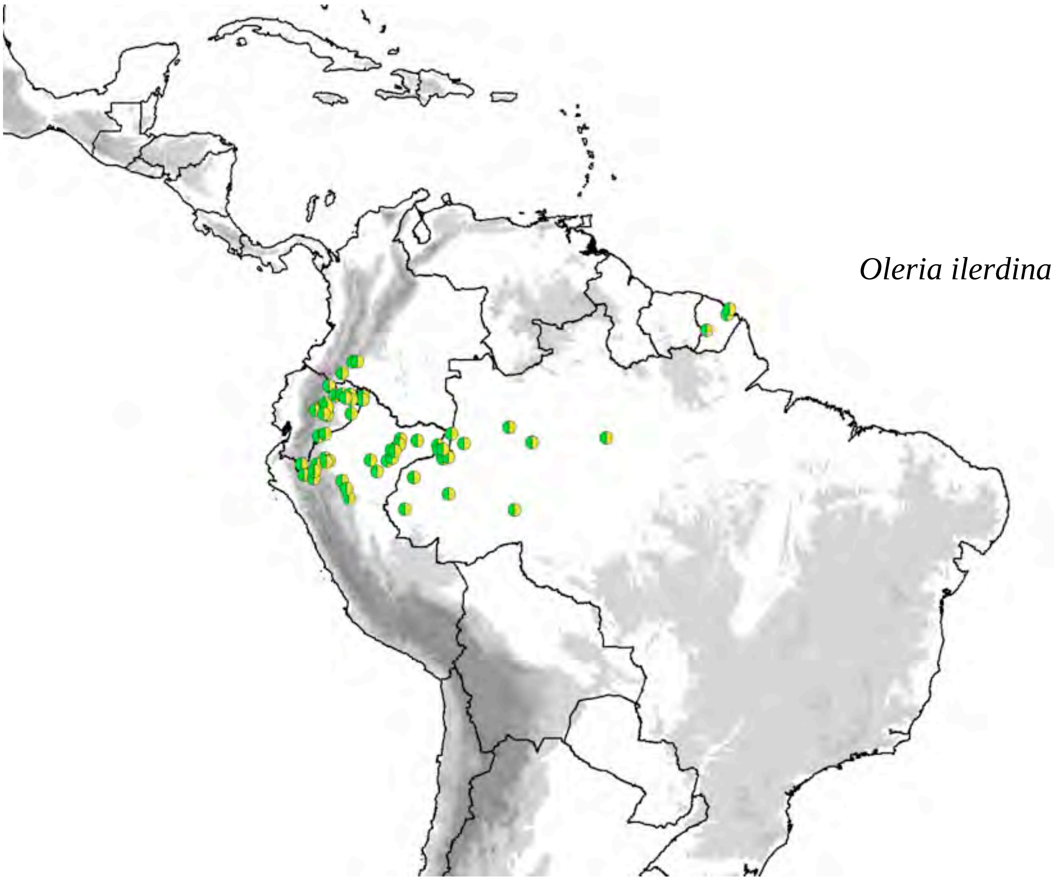

Oleria ilerdina

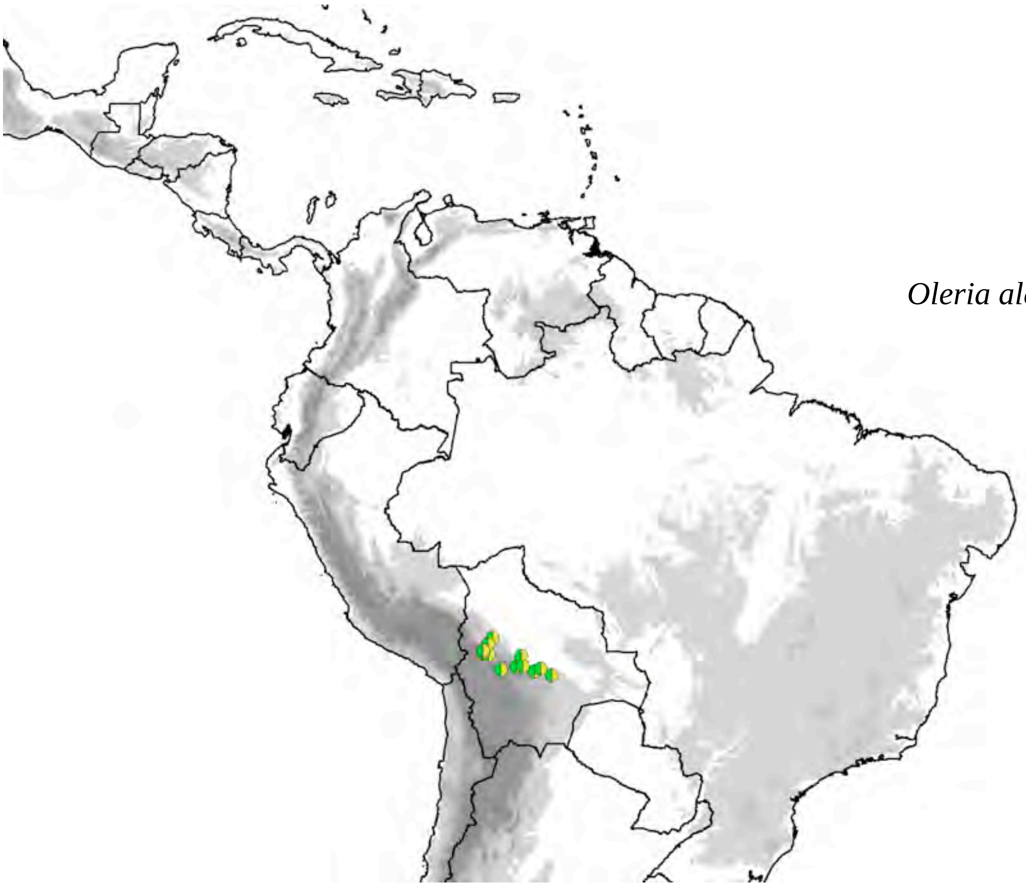

Oleria alexina

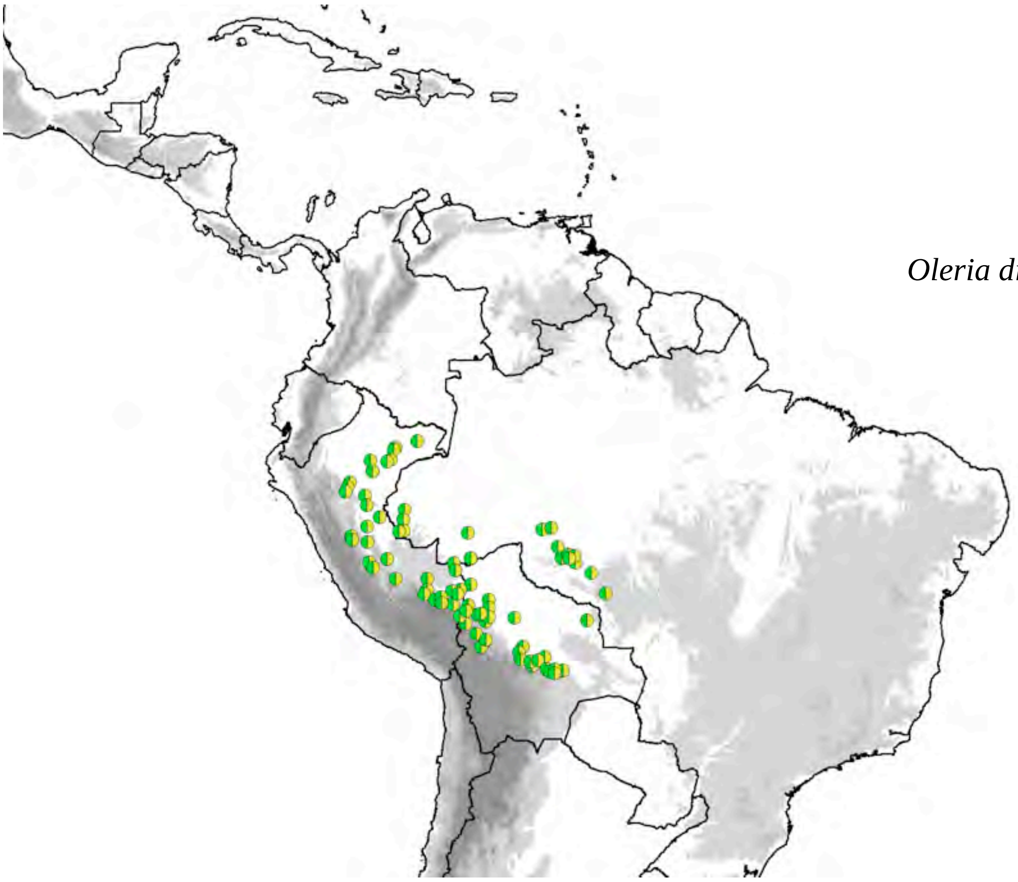

Oleria didymaea

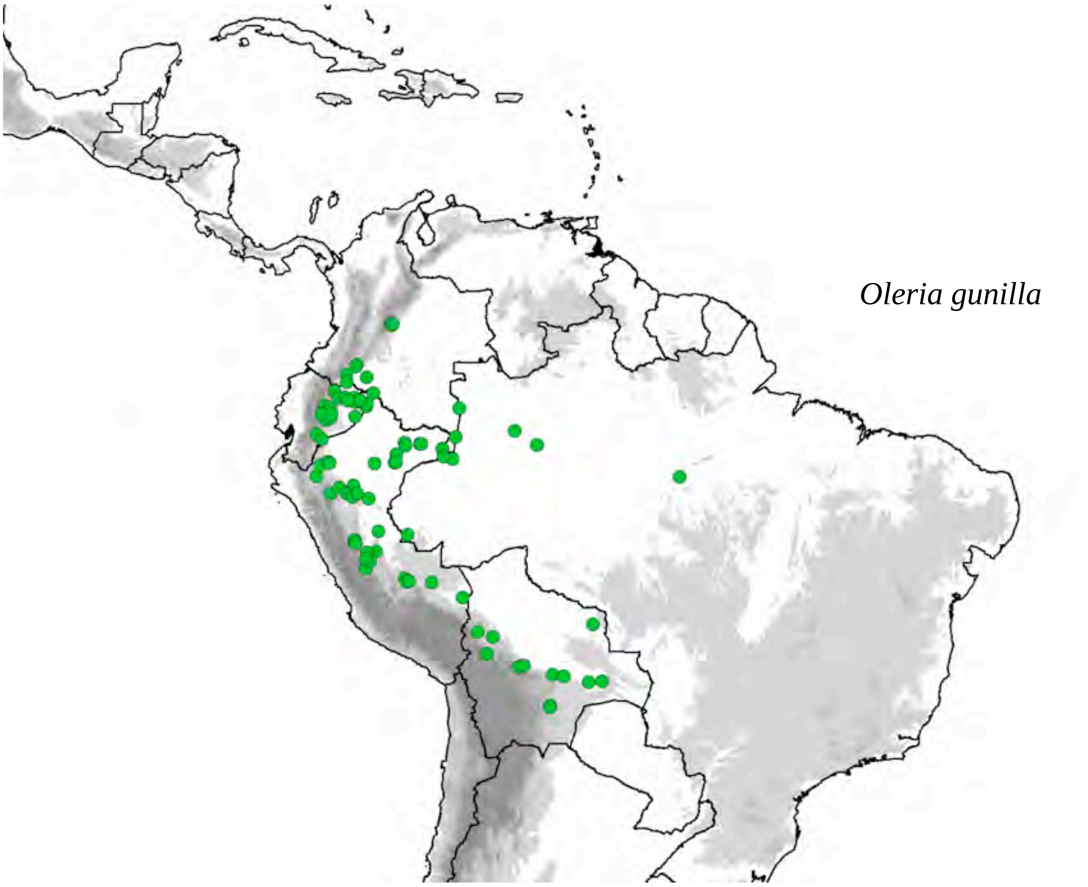
Oleria gunilla

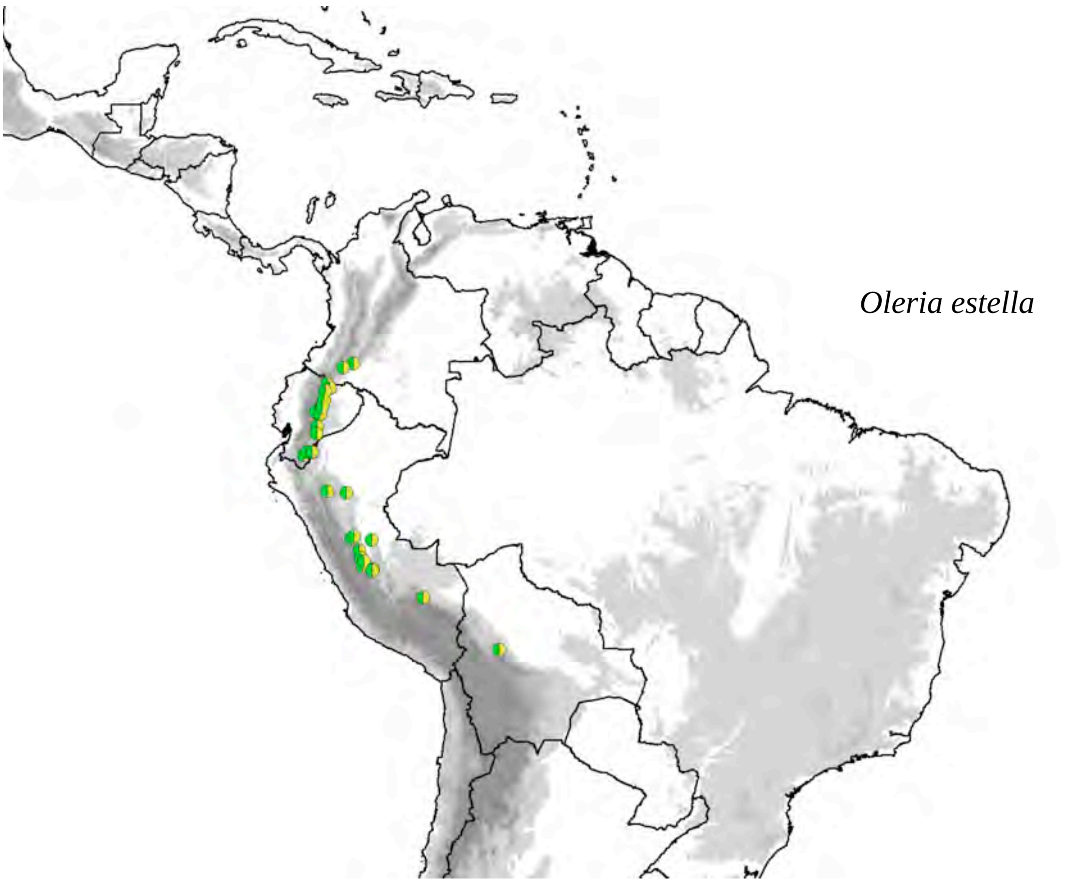
Oleria estella

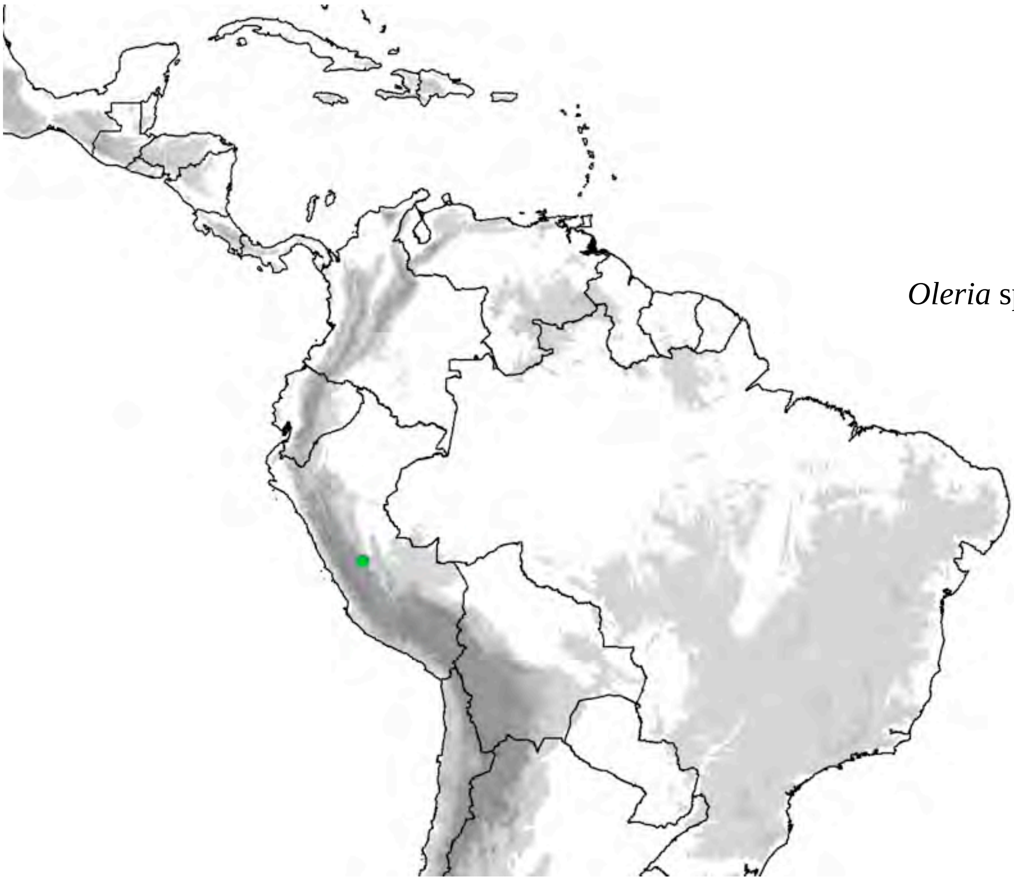

Oleria sp. nov. 1

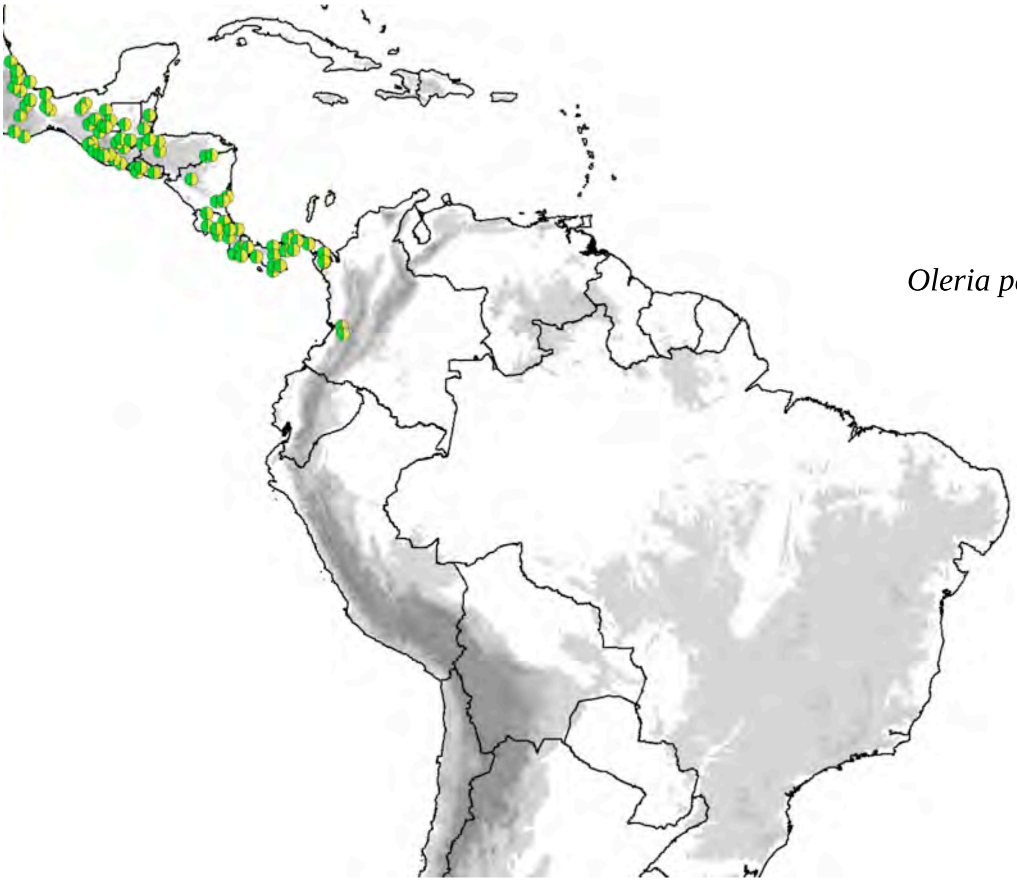

Oleria paula

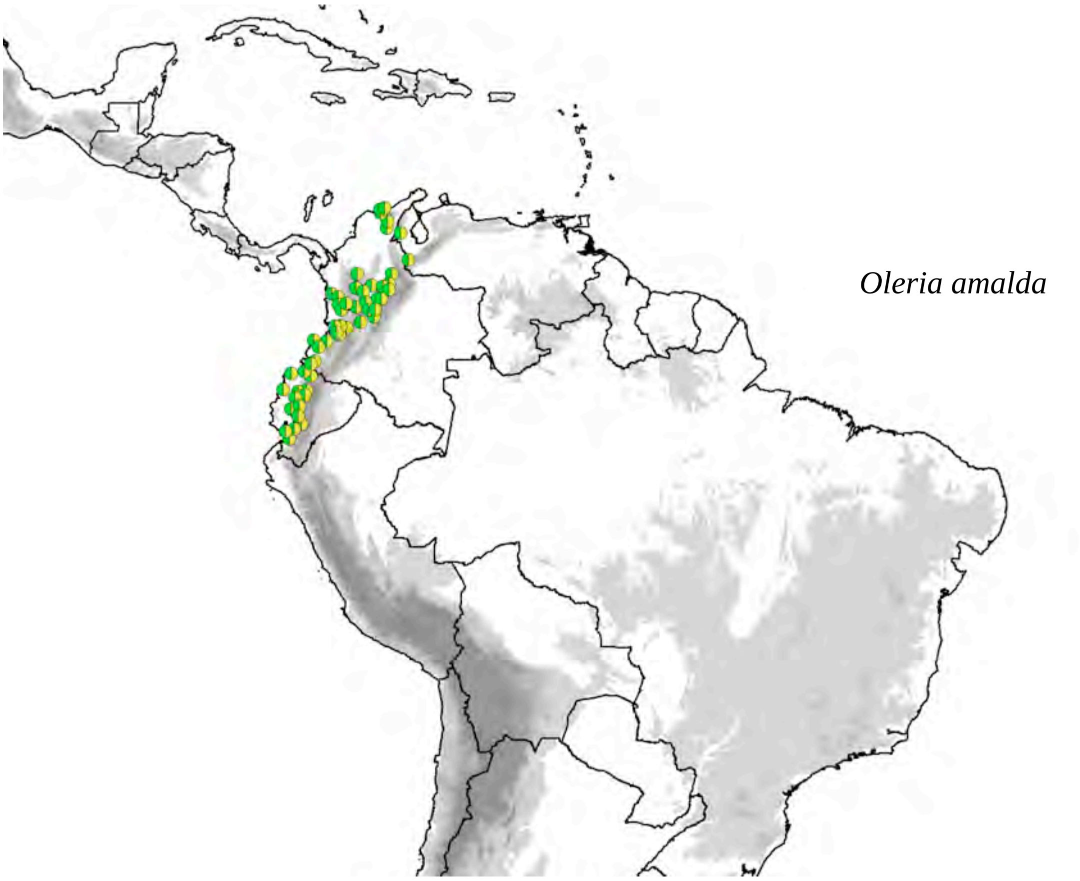

Oleria amalda

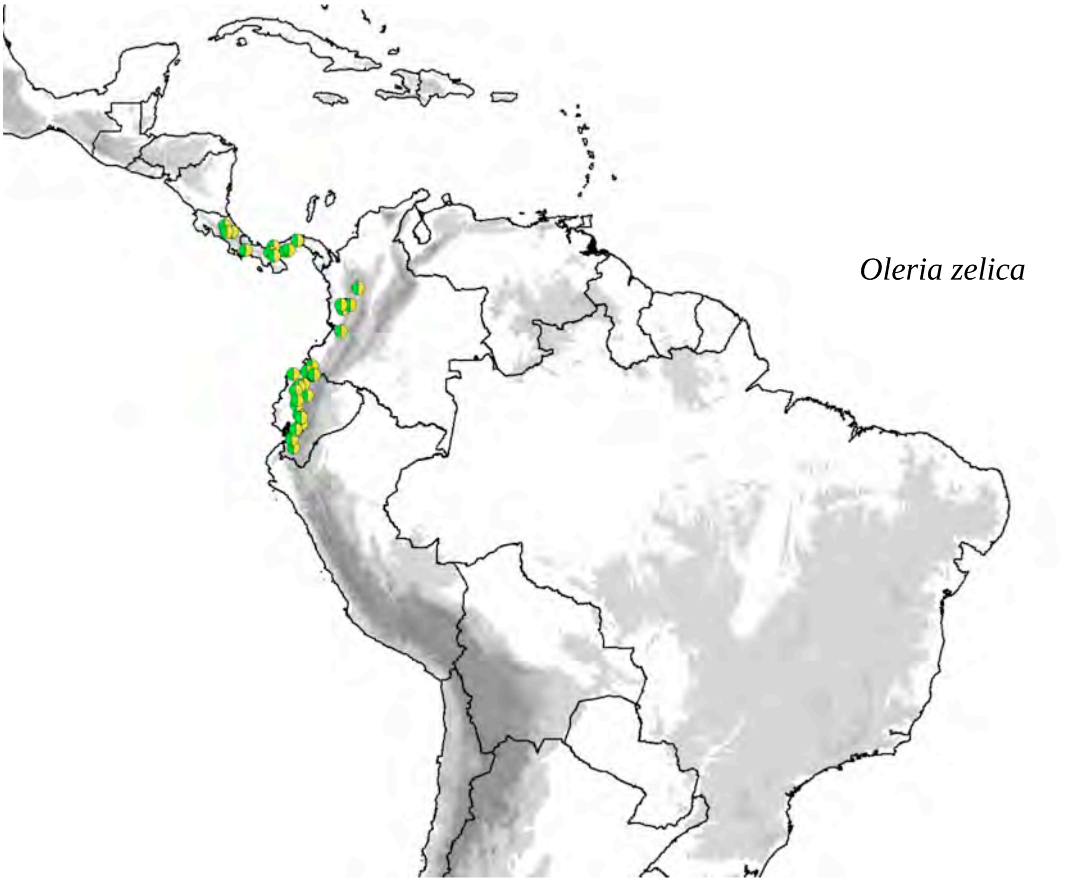

Oleria zelica

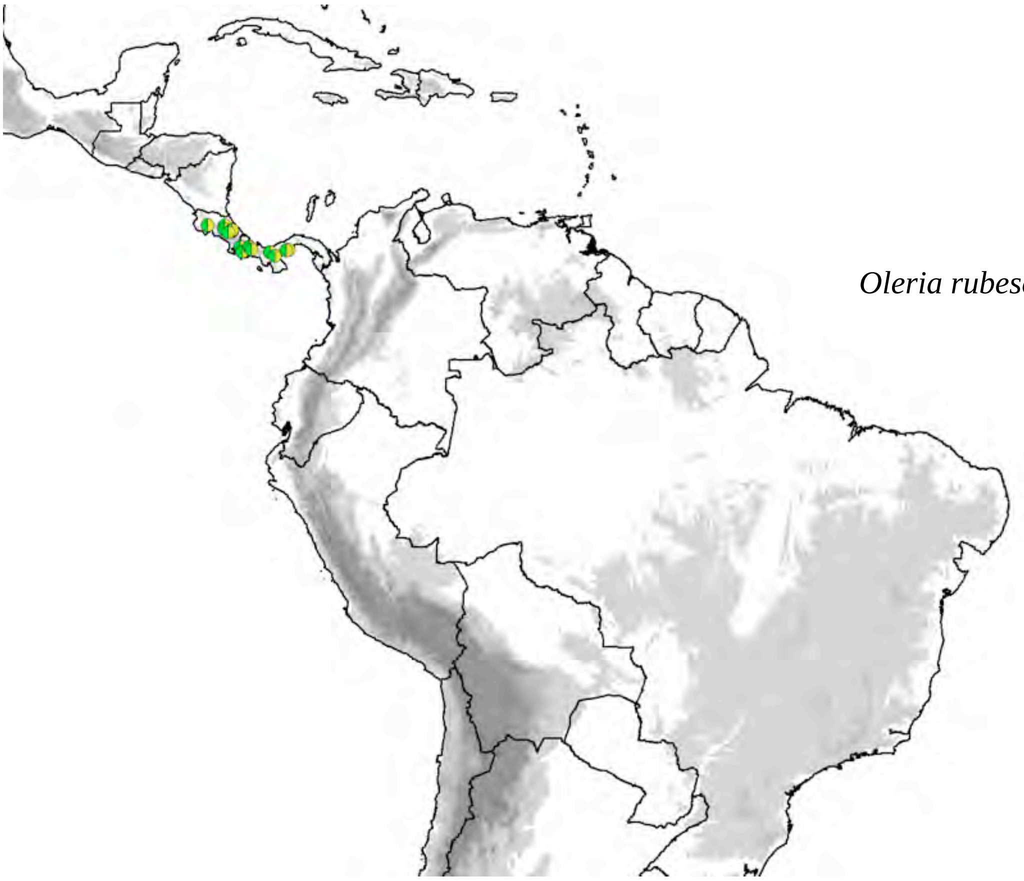

Oleria rubescens

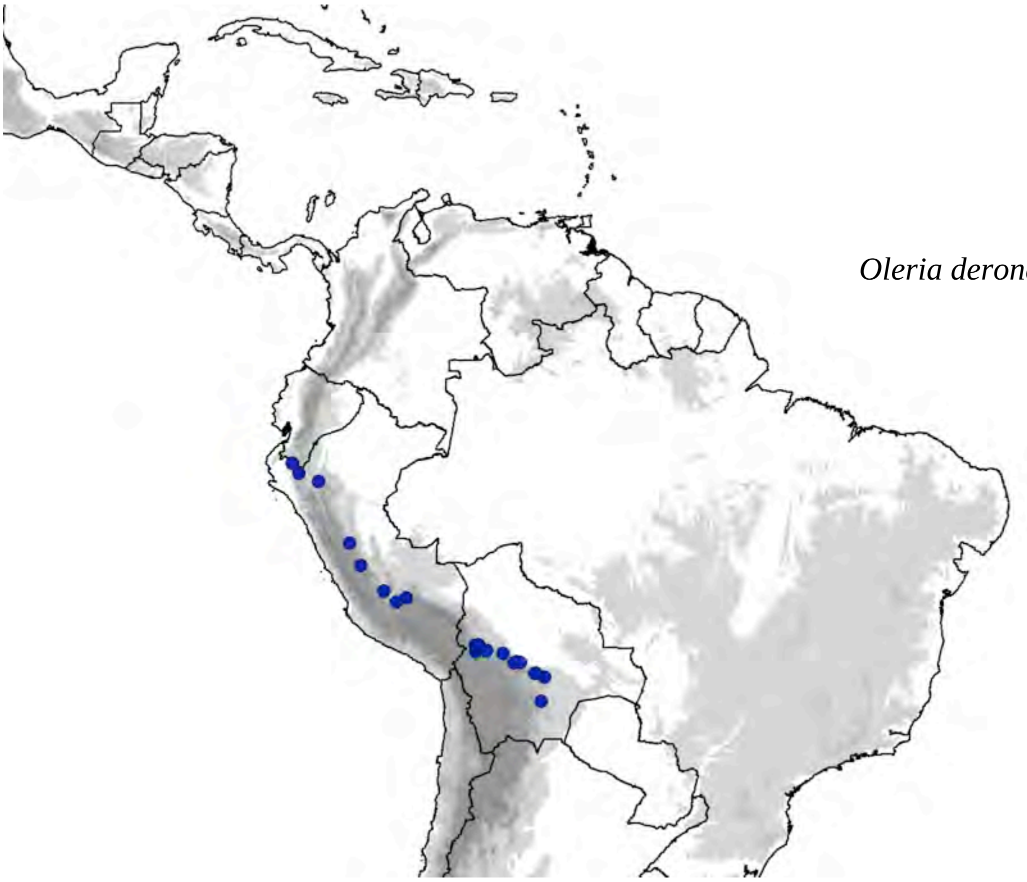

Oleria derondina

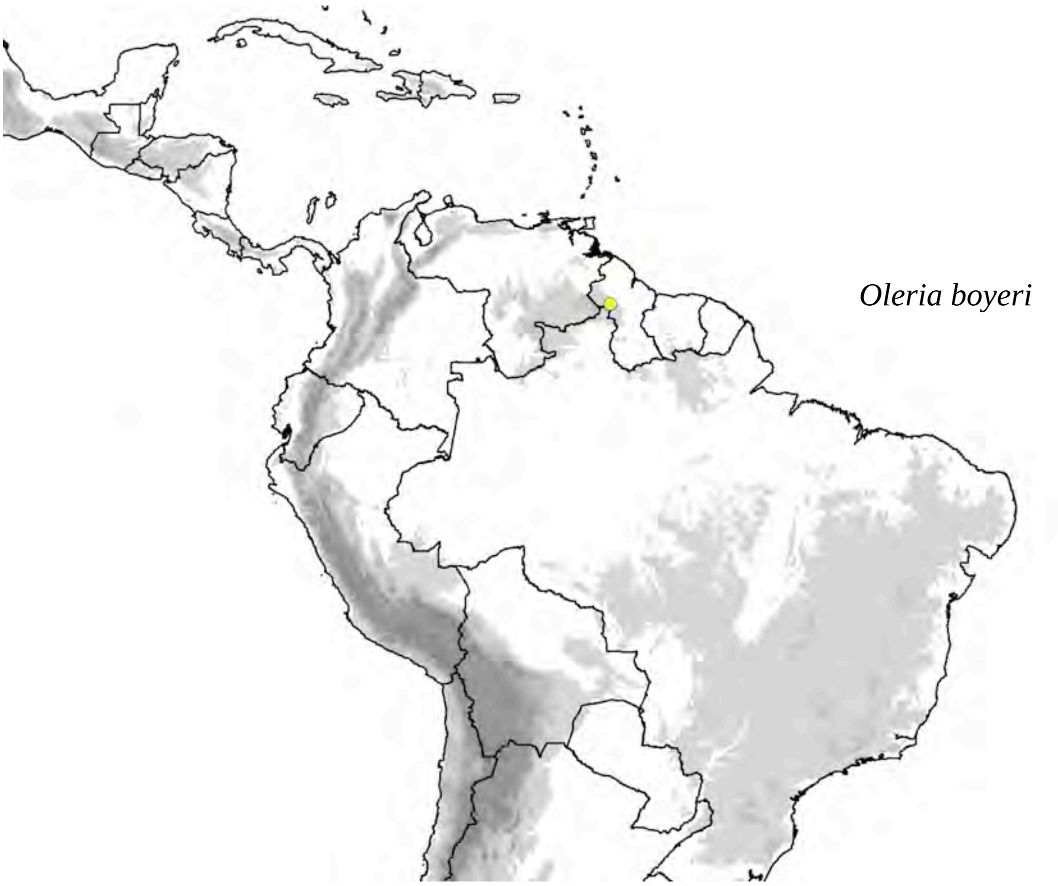
Oleria boyeri

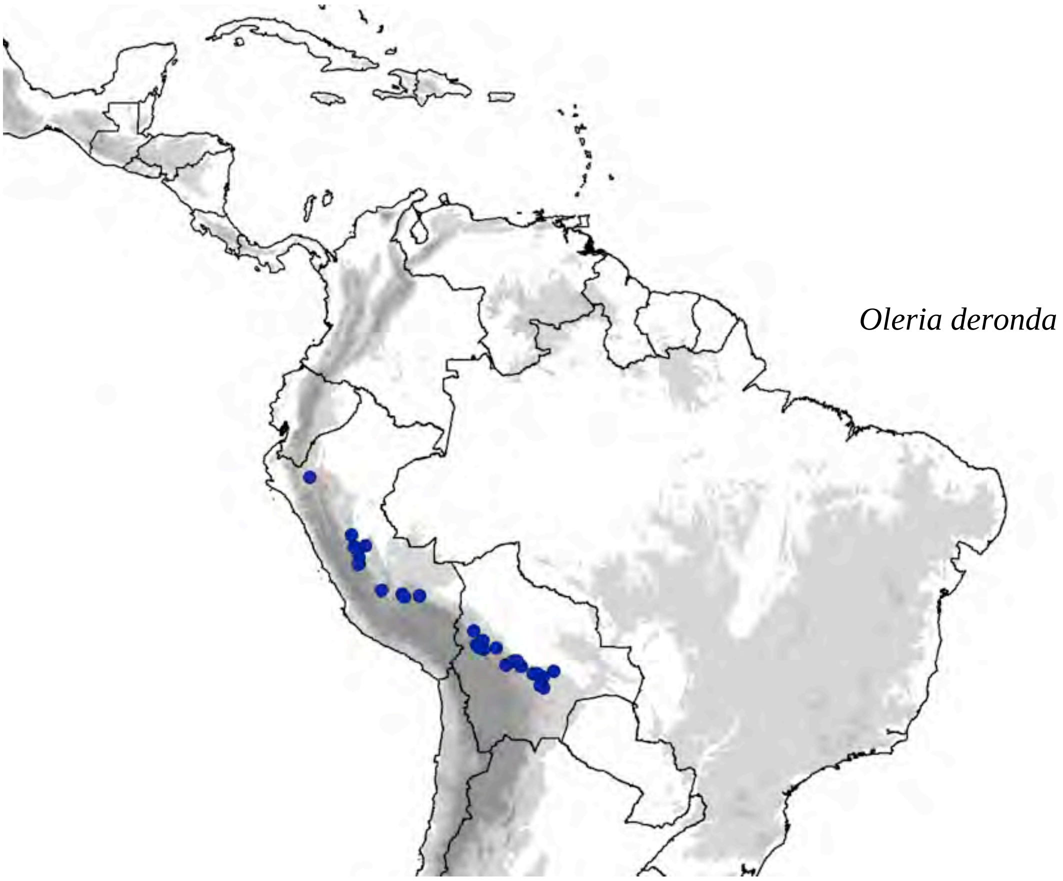
Oleria deronda

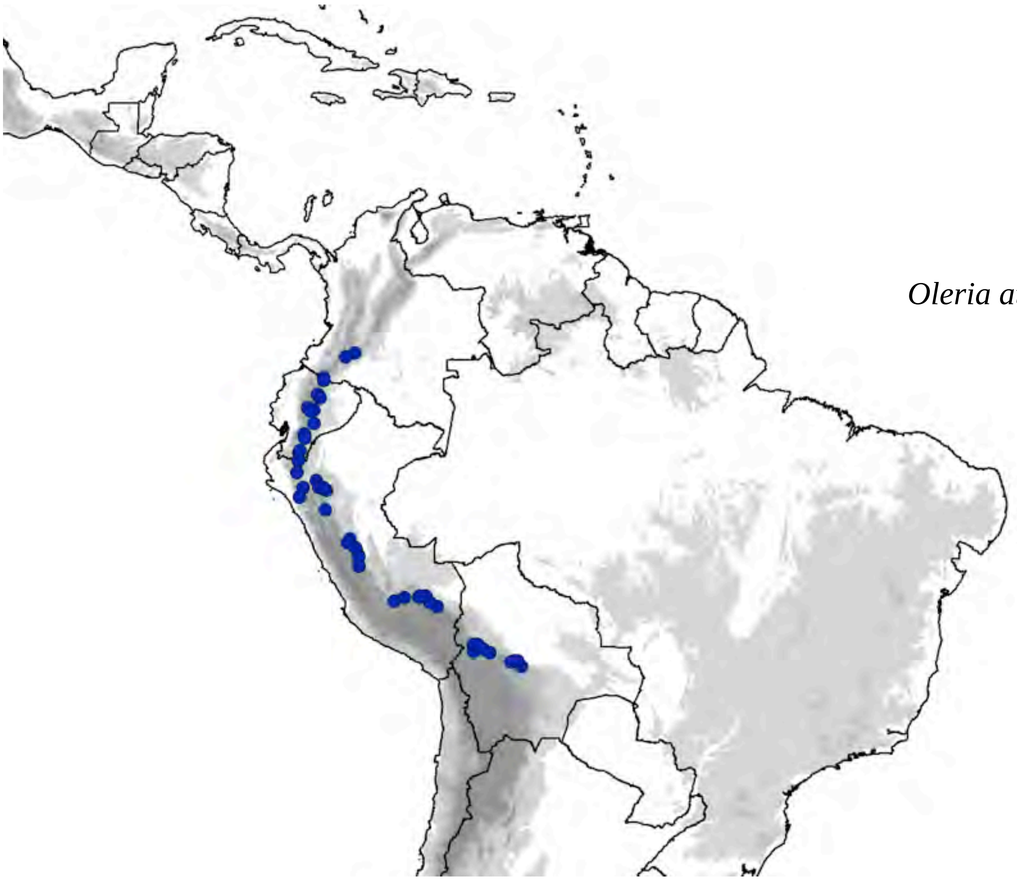

Oleria athalina

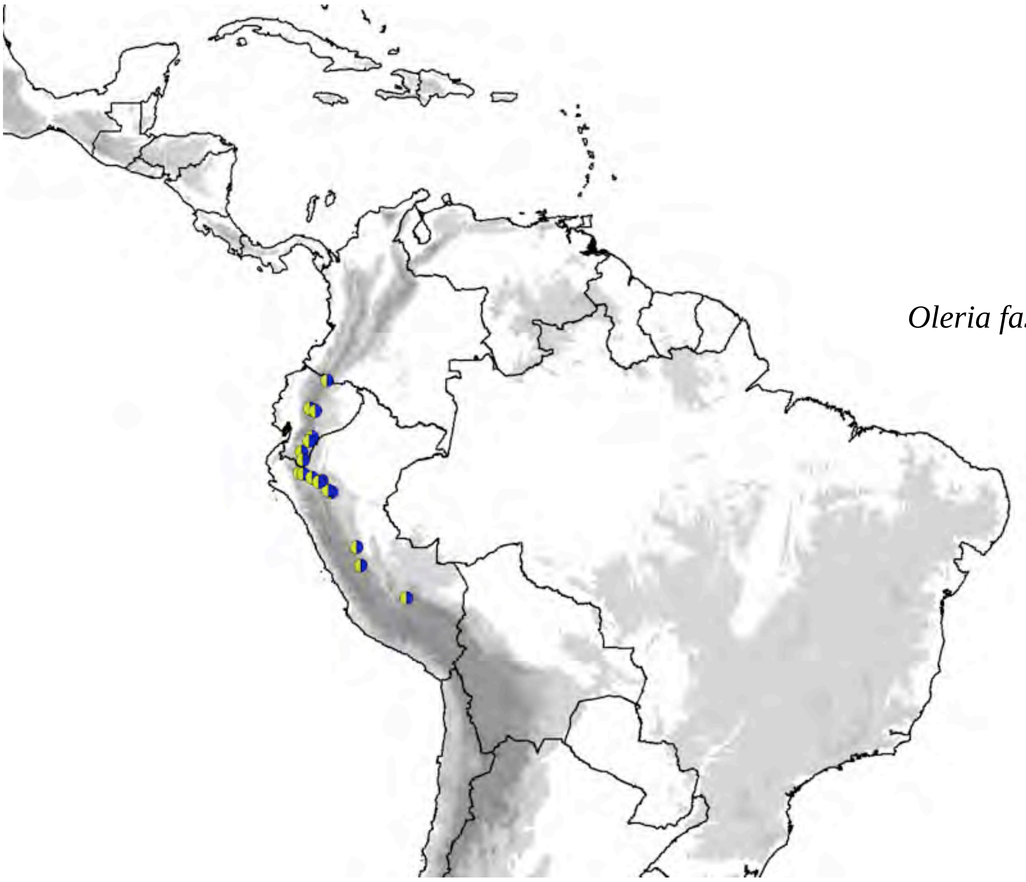

Oleria fasciata

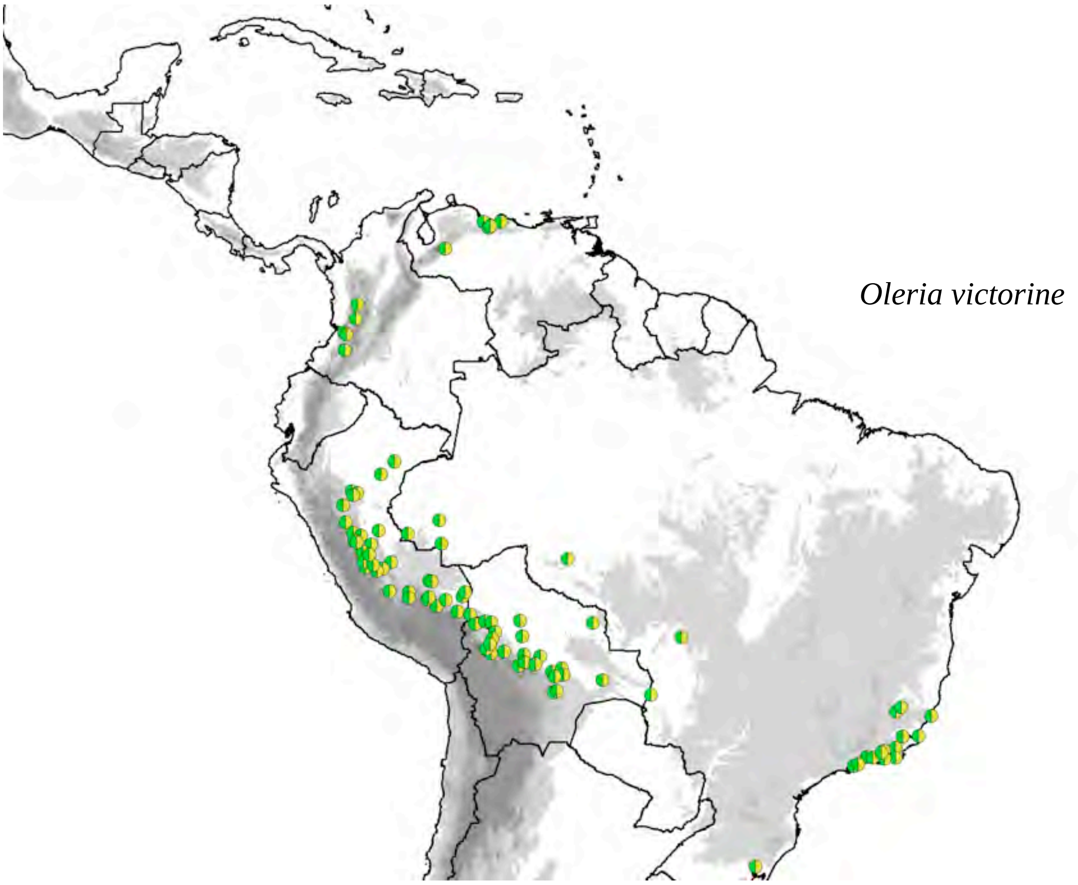

Oleria victorine

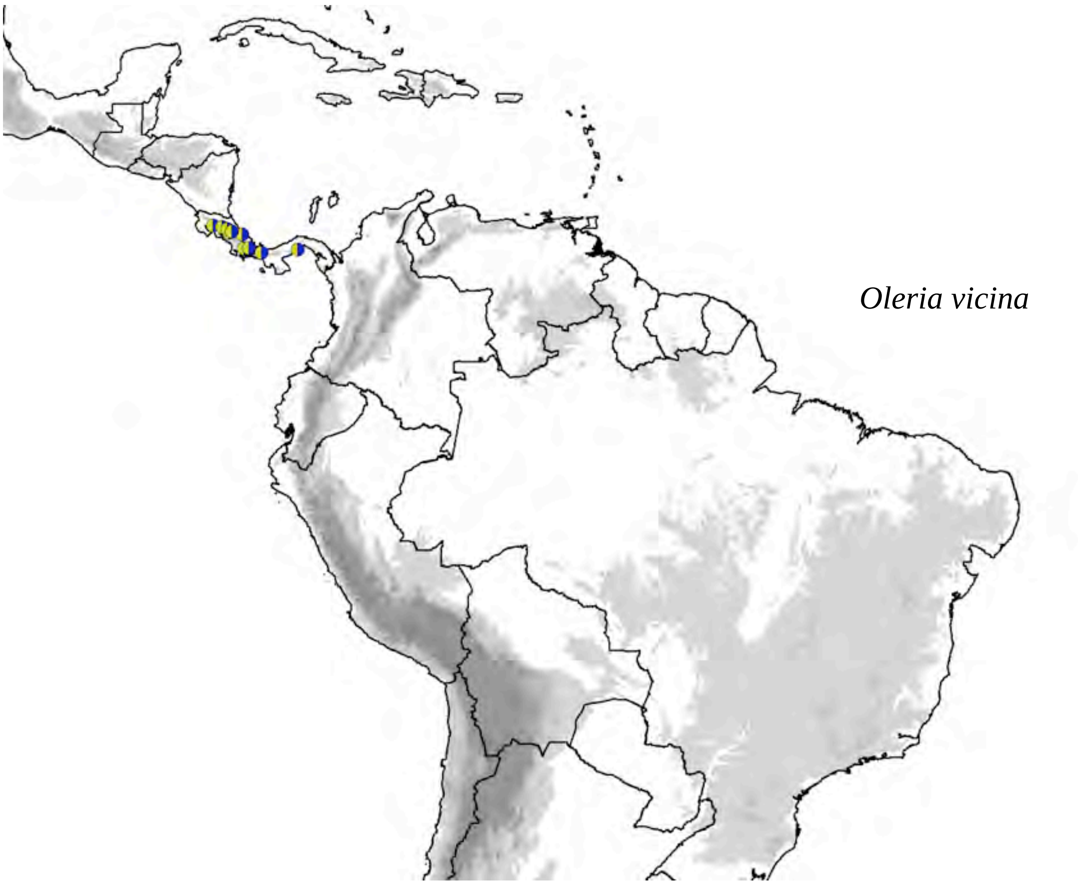

Oleria vicina

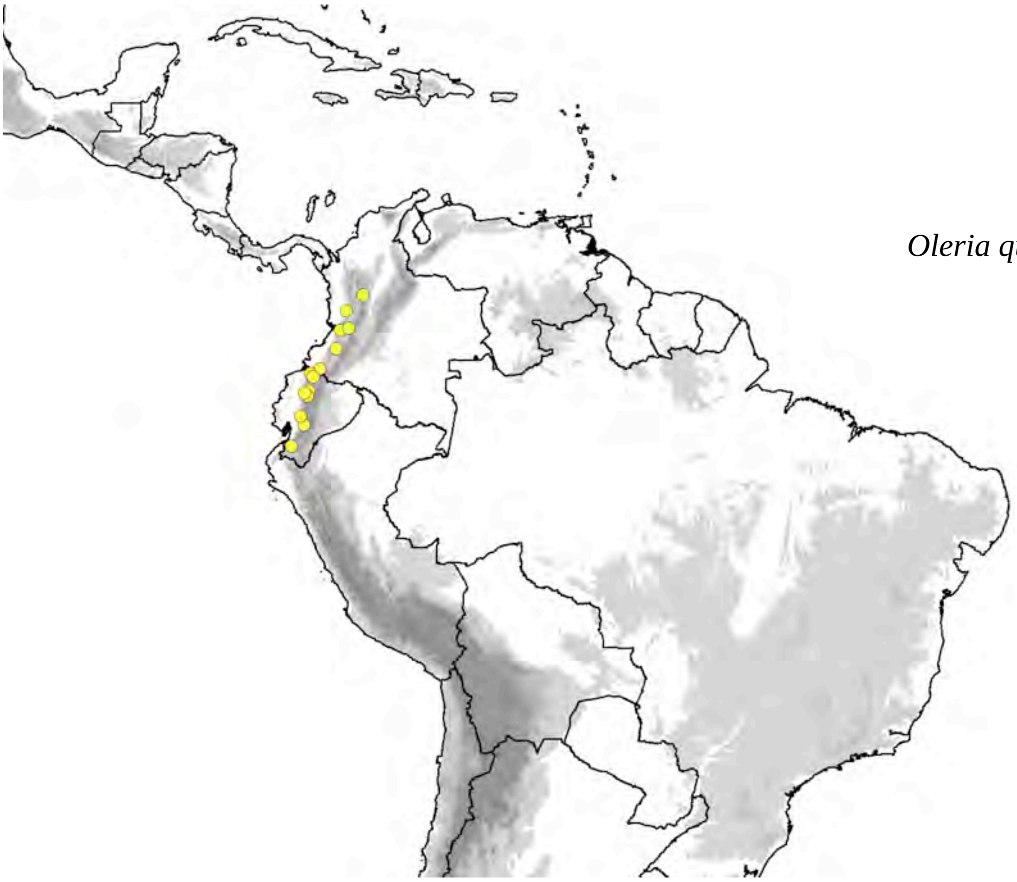

Oleria quadrata

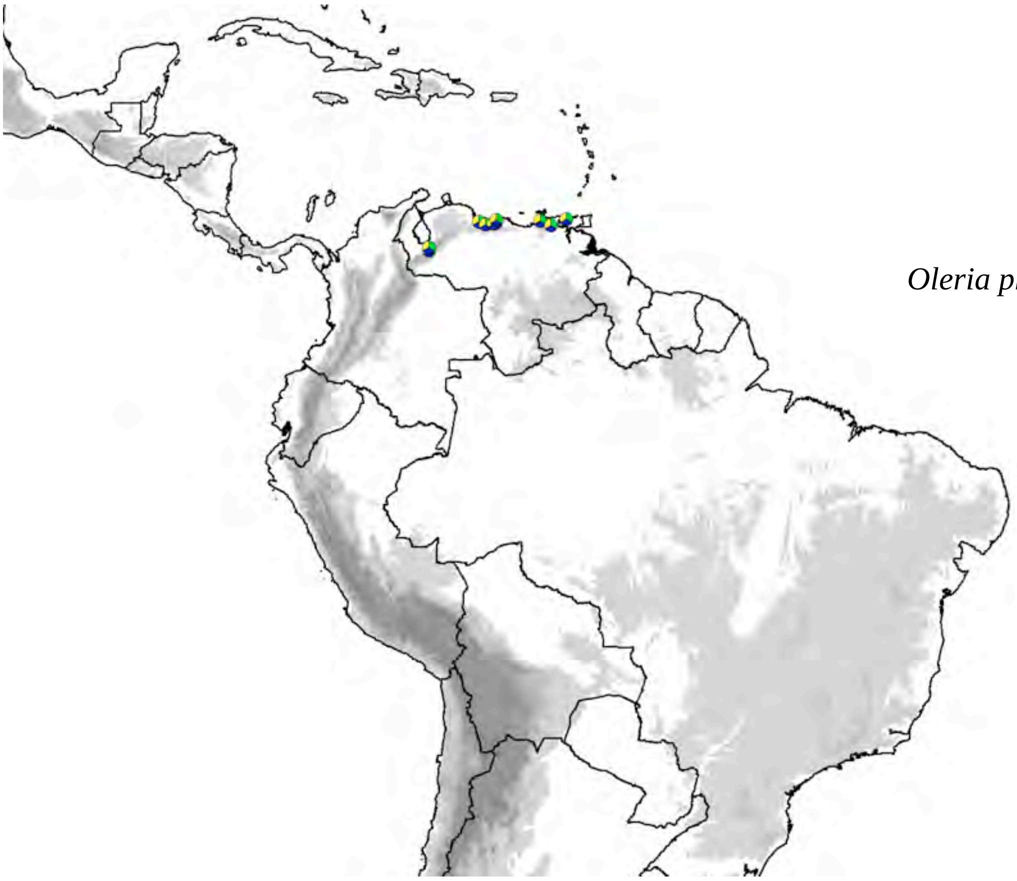

Oleria phenomoe

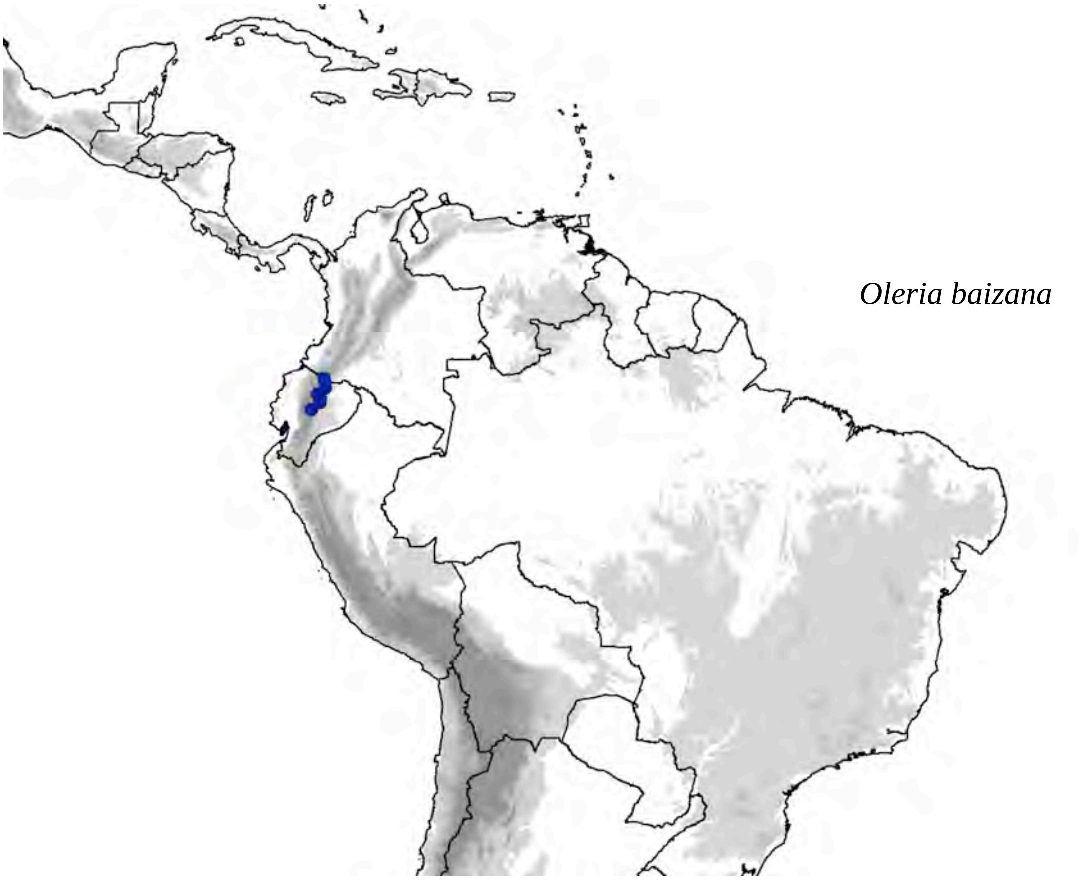

Oleria baizana

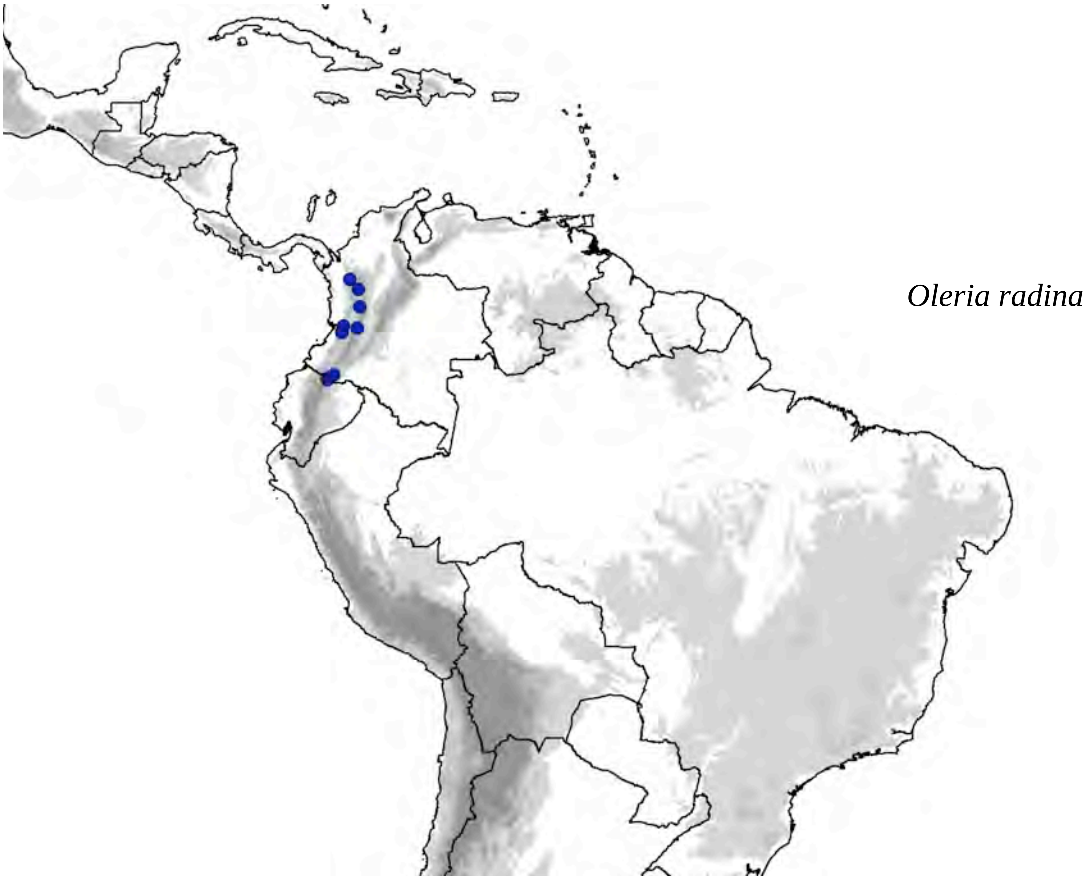

Oleria radina

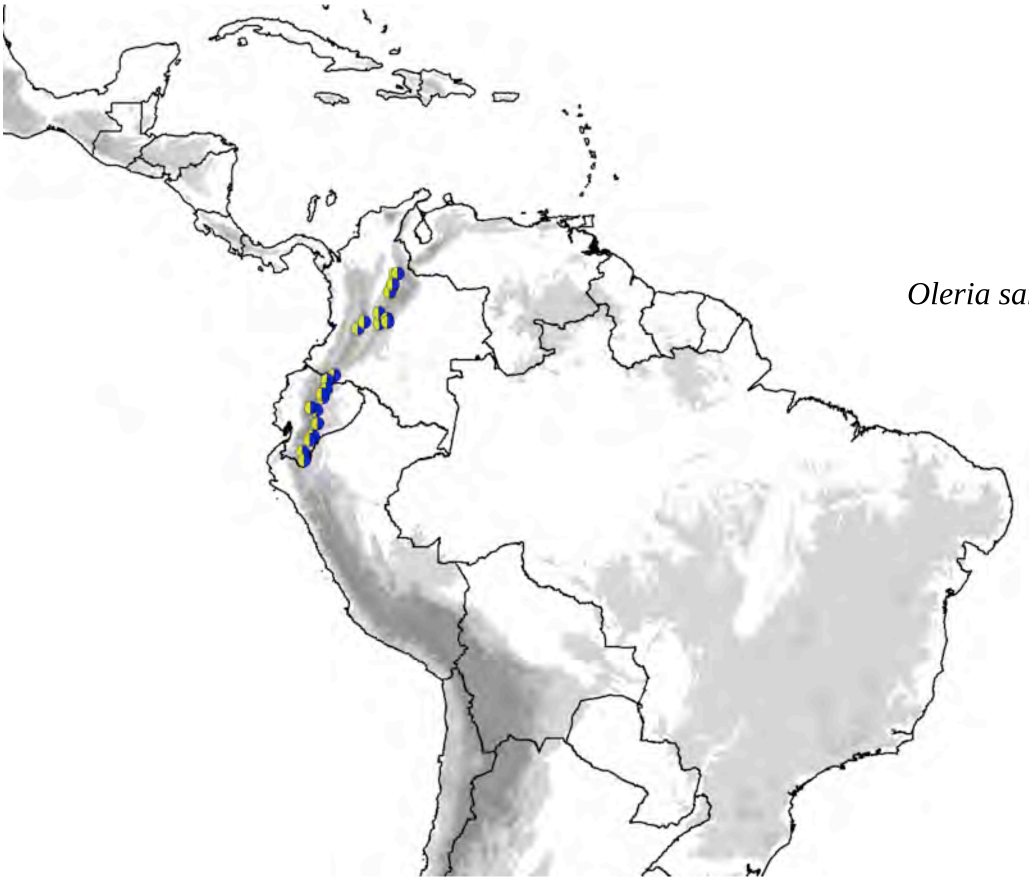

Oleria santineza

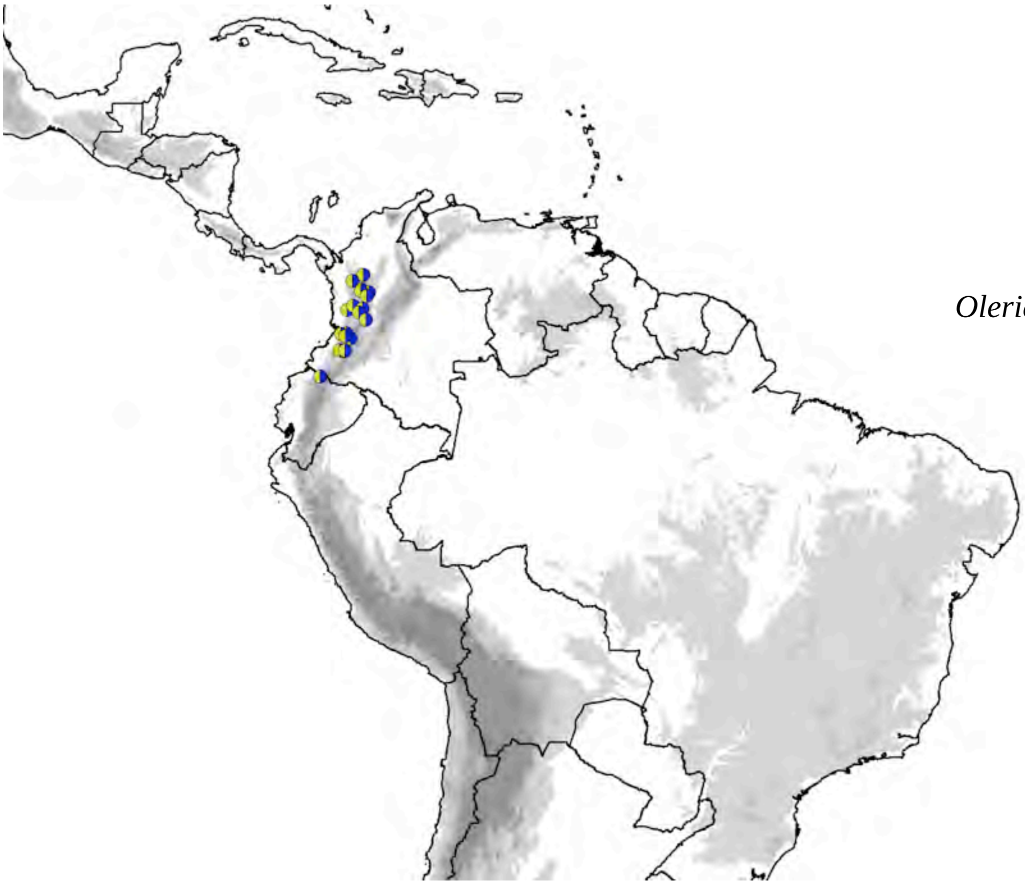

Oleria fumata

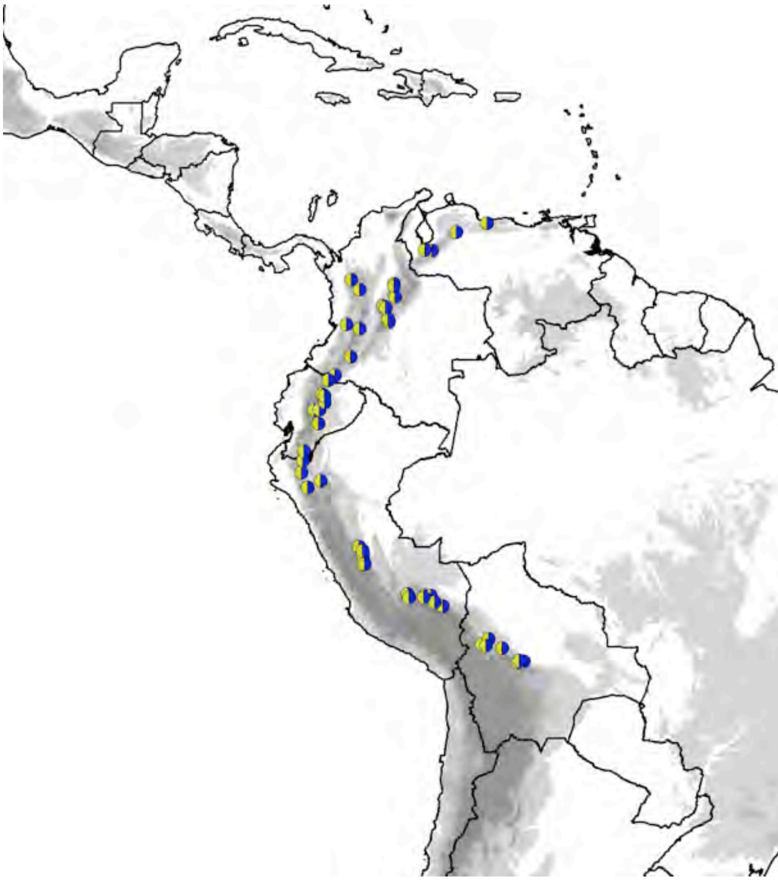

Oleria tremona

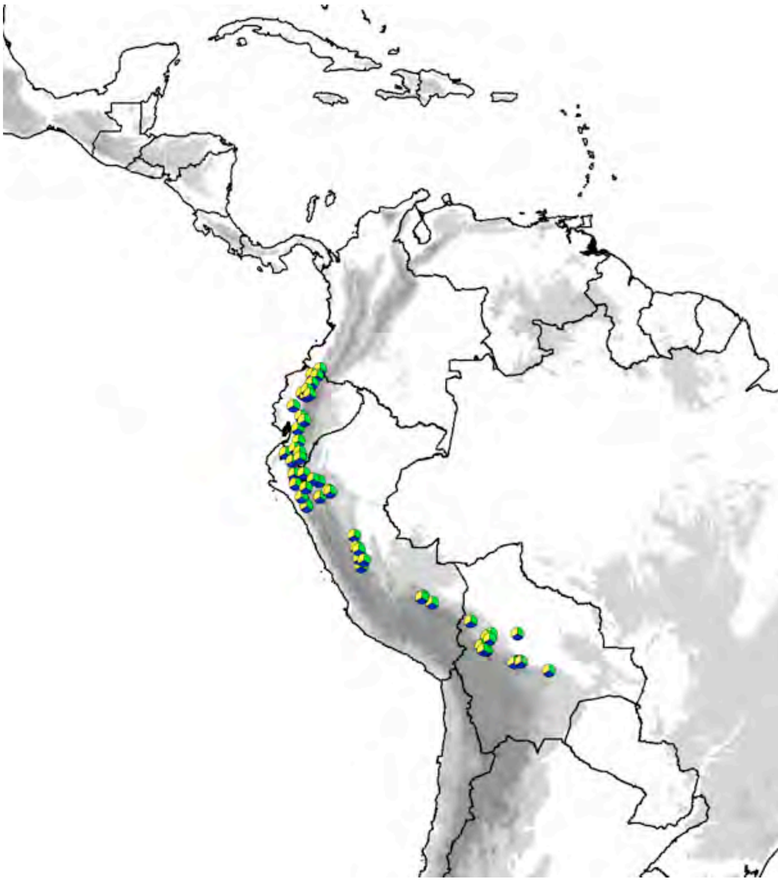

Oleria padilla

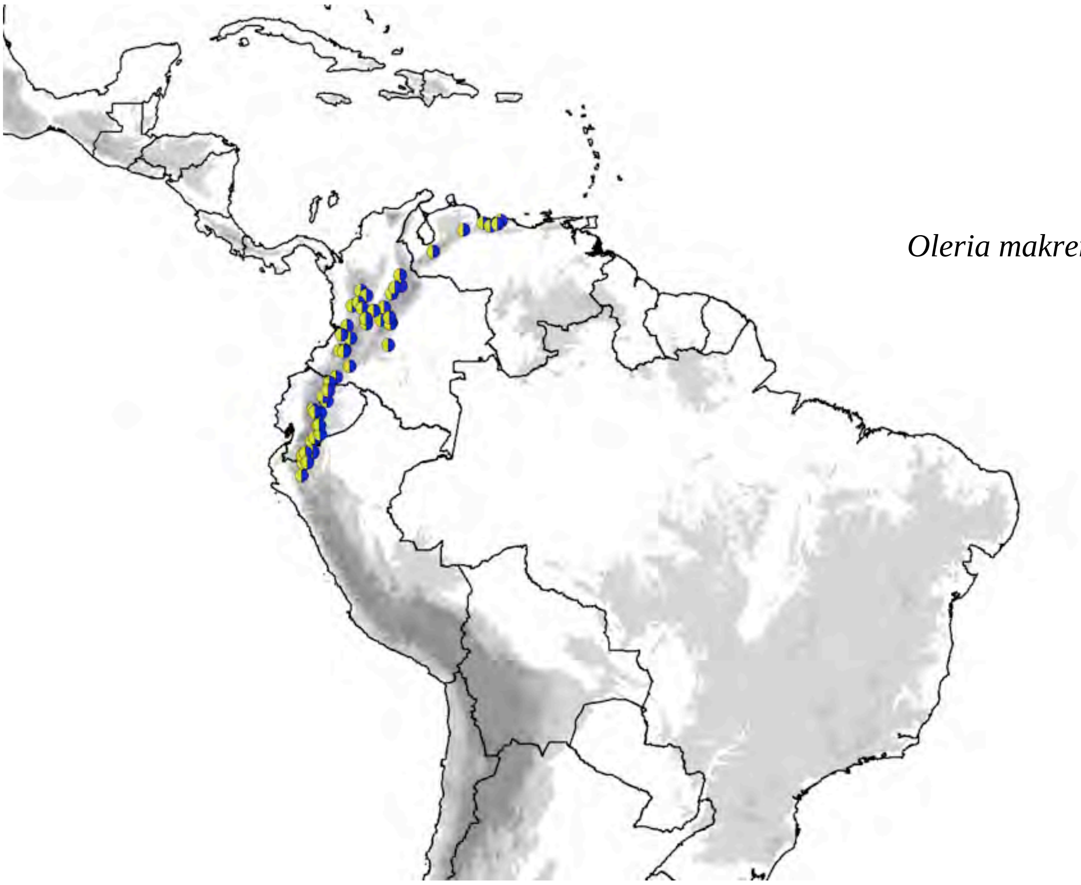

Oleria makrena

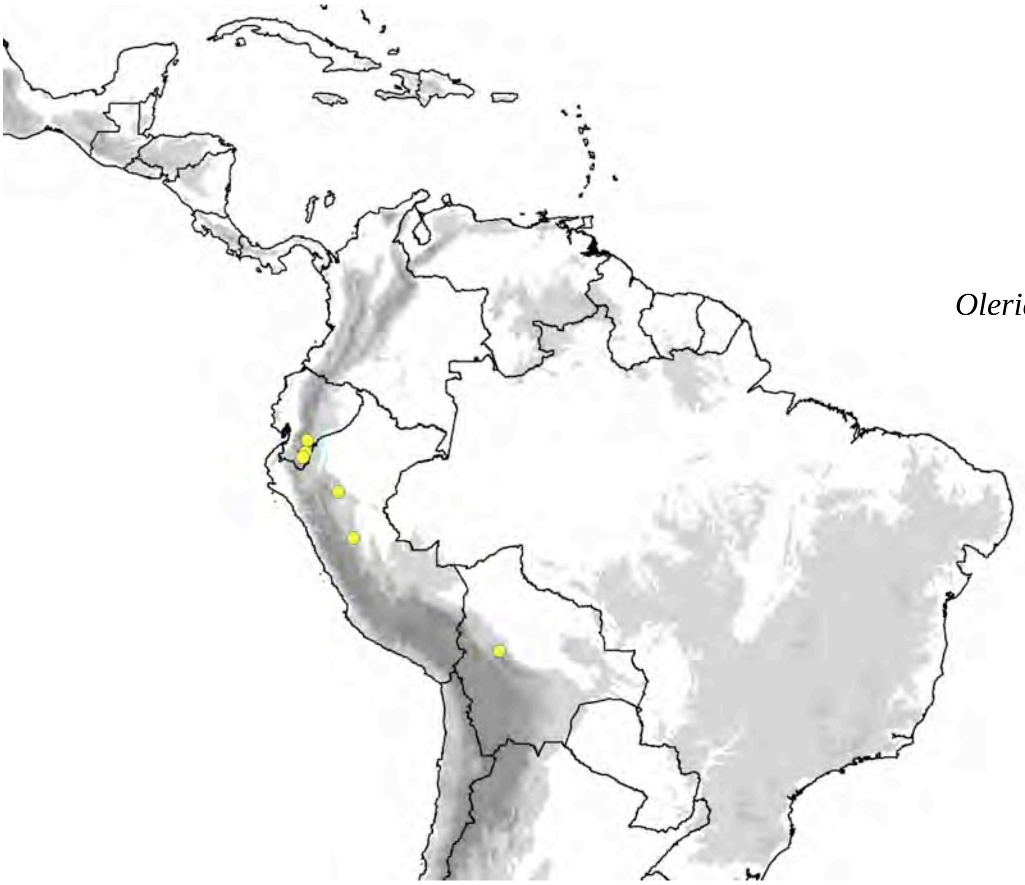

Oleria bioculata

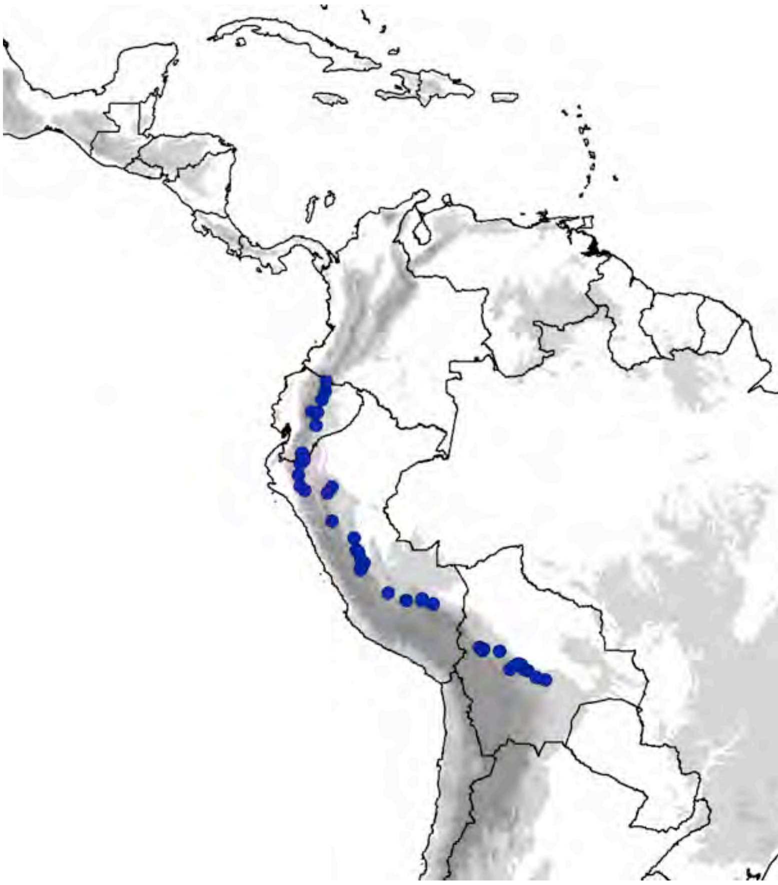

Oleria attalia

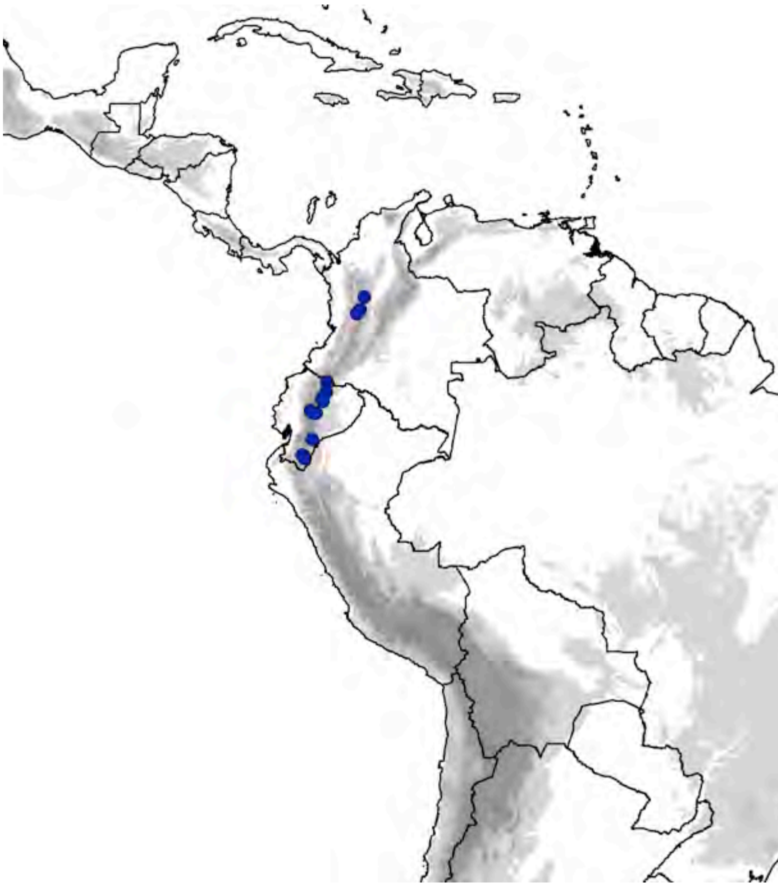

Oleria cyrene
